# Determinants of under-five mortality in Africa: evidence from a two-decade panel analysis for public health policy

**DOI:** 10.1186/s13690-026-01994-0

**Published:** 2026-06-17

**Authors:** Himanshi Rathnasekara, Ruwan Jayathilaka

**Affiliations:** SLIIT Business School, Sri Lanka Institute of Information Technology, New Kandy Road, Malabe, Sri Lanka

**Keywords:** Under-5 mortality, Africa, Fertility, Health expenditure, Sanitation, Panel regression

## Abstract

**Background:**

Child survival is a critical indicator for a nation’s health and its progress is important in attaining the Sustainable Development Goals. Understanding the regional and country-specific dynamic and interplay of various determinants of under-five child mortality is vital for the African continent, which remains one of the most vulnerable regions for child mortality globally.

**Methods:**

This study investigates the association of economic, health-related, social and demographic, environmental, and infrastructure-related factors with under-five child mortality. It integrates generalisable regional associations using a standard fixed-effects panel model and examines illustrative country-specific associations of the selected determinants through multiple linear regression, based on a balanced panel dataset of 45 countries over a 22-year period.

**Results:**

Fixed-effect analysis reveals that the diphtheria-tetanus-pertussis (DTP) immunisation and total fertility rate (TFR) are robust regional determinants of under-five mortality across specifications. While health expenditure, sanitation services, and malaria incidence show significant associations in the baseline model, these findings are sensitive to the inclusion of year fixed effects, suggesting they are influenced by broader temporal trends or common regional shocks rather than serving as stable independent factors within the study period.

**Conclusion:**

Regional analysis, which controls for unobserved country-specific heterogeneity over an extended period and is complemented by country-specific analysis, facilitates the formulation of policy implications at both national and international levels. Recommended policy measures include increasing immunisation coverage, implementing malaria control programmes, strengthening community health infrastructure, enhancing girls’ education, promoting widespread access to modern family planning, and improving sanitation services. These strategies are expected to contribute to the progress toward Sustainable Development Goal 3.2 in the African region.

**Supplementary Information:**

The online version contains supplementary material available at 10.1186/s13690-026-01994-0.

**Table:** 

Textbox 1. Contributions to literature
• Presents a two-decade panel analysis (2000–2021) across 45 African countries, identifying multi-dimensional economic, health, demographic, and environmental factors associated with under-five mortality.
• Employs a fixed-effects panel model specification to control for unobserved heterogeneity, offering generalisable regional evidence alongside country-specific insights.
• Reveals the dual role of fertility dynamics and malaria burden as persistent determinants while highlighting the diminishing returns of economic growth for child survival.
• Bridges research and policy by outlining actionable interventions, expanded immunisation, malaria control, sanitation, and reproductive-health initiatives to support SDG 3.2 progress in high-risk African nations.

## Background

### Background and its significance

Human mortality is a significant determinant in managing the population growth of a particular country [1]. The under-five mortality rate (U5MR) is defined as the number of deaths of children under five years of age per 1,000 live births. The infant mortality rate and U5MR are widely used indicators to track child health globally. There is a significant 59% drop in U5MR reported since 1990. The United Nations Sustainable Development Goals (SDG) 3.2 targets the elimination of preventable child deaths or the reduction of U5MR to at least 25 deaths per 1000 live births by 2030 [2]. However, according to the predictions, 59 countries are expected not to meet the required SDG target for reducing child mortality by 2030.

### Current situation in Africa

**Fig. 1:**
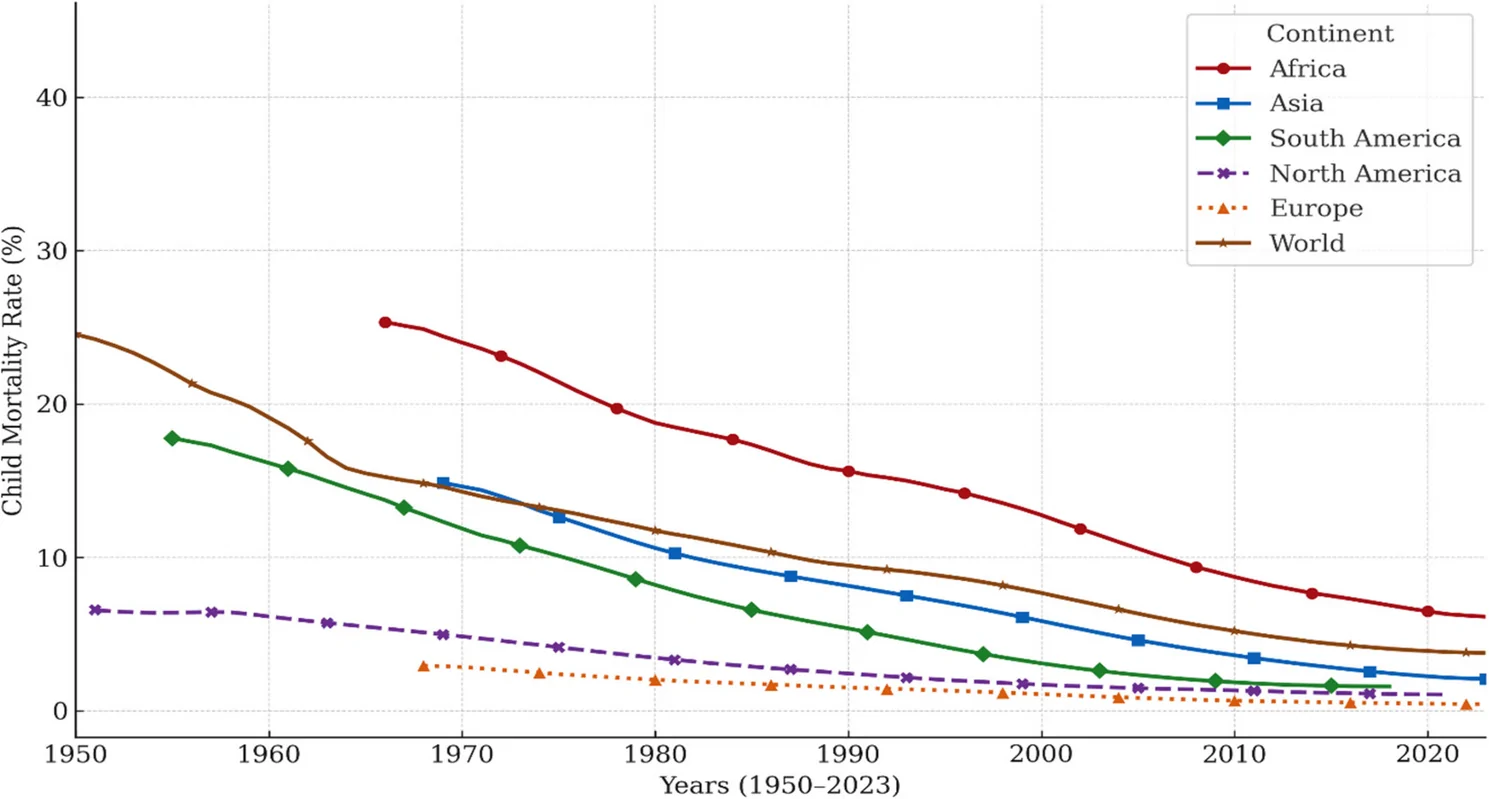
Comparison of global child mortality rates across different continents and the world average (1950–2023). Source: Authors’ illustration based on the data published in Our World in Data [8]. Note: Data represents the probability of a child dying before reaching age five

The average Under-5 Mortality Rate (U5MR) in Africa is 61.8 per 1000 live births. However, this rate exhibits significant disparities across the African continent [3]. Although the expansion of healthcare services has substantially reduced childhood mortality in Africa over the past 65 years, it is projected that efforts will need to continue until approximately the year 2050, to lower the current rate to 25 deaths per 1000 live births and attain the standards observed in contemporary Asia. Furthermore, Sub-Saharan Africa (SSA) has been identified as the epicentre of the persisting child mortality crisis, as most countries in the SSA region face a critical situation in realising the SDGs by 2030. If the current trend continues, it has been estimated that nearly 60% of child deaths in the world will be reported from SSA within the decade of 2020–2030 [4]. Moreover, as high child mortality can be seen in South Asia (SA) and SSA before 28 days and after 6 months, they show two or three times higher neonatal deaths than the rest of the world [5]. As statistics revealed in 2022 and 2023, SA and SSA have recorded more than 80% of U5MR, whilst the highest neonatal mortality rate has been recorded from SSA. The possibility of a newborn dying in SSA within the first month of birth is 14 times higher than in regions like New Zealand and Australia [6]. It reflects the silent crisis that a child’s survival depends on where they are born. Consequently, this tragedy needs to be addressed by the African continent. The child mortality rate in different continents worldwide is shown in Fig. 1, indicating the status of Africa, despite the global decline. Furthermore, there is an urgent need to address the issue by increasing investment in the girl child [7], particularly in education.

### Policies and global effects

The Integrated Management of Childhood Illnesses strategy was implemented by the World Health Organisation (WHO) in collaboration with the United Nations International Children’s Emergency Fund (UNICEF) in 1990 to reduce child mortality. Actions to be taken at the family, community, and institutional levels have also been established, including promoting health education and improving access to healthcare services. This approach was effective and efficient in reducing child mortality [9]. This implies the steps taken to control the U5MR on a global scale.

### Objective of the study

This study aims to investigate the association between multidimensional determinants and the U5MR in the African continent over two decades, leveraging this to identify the regional associations of key determinants and country-specific factors. The study focuses on multiple areas in terms of per capita Gross Domestic Product (GDP), Diphtheria, Pertussis & Tetanus vaccination (DTP), Incidence of Malaria (IOM), Current Health Expenditure (CHE), Adolescence Fertility Rate (AFR), Total Fertility Rate (TFR) and Basic Sanitation Services (BSS). By controlling for unobserved country-specific and time-invariant heterogeneity, this study aims to present the average and generalisable associations of key determinants at the regional level and explore country-specific associations.

### Contribution of the study

The results of this study contribute to existing knowledge in three respects. First, this research presents a framework for considering factors affecting multidimensional coverage over an extended 22-year period. Since it spans over two decades, it represents the actual dynamic pattern of the considered variables. Therefore, the results aim to obtain more reliable outcomes.

Second, there is a significant gap in existing knowledge regarding the independent association of each factor on U5MR; this study identifies generalisable regional associations through panel regression. This can be realised through this analytical model specification, as it controls the unobserved, time-invariant heterogeneities that are authentic for the considered countries, such as their cultural norms, traditional practices and myths. Hence, the results of this study contribute to the existing knowledge by reducing bias and providing associative results.

Third, the persistent heterogeneity in the declining nature of U5MR across the African continent at different rates implies the importance of implementing country-specific policy interventions not only on a short-term basis but also in the long term. This country-specific approach through Multiple Linear Regression (MLR) will be employed to support to the high-risk, susceptible countries in the African region in contributing to the progress toward SDG 3.2 through the implementation of suggested policies. Apart from the ministries of relevant countries, the results of this study assist the international organisations, like the WHO and UNICEF, by directing them to necessary actions to eradicate the current tragedy of child deaths in the world.

The paper begins with a critical review of existing literature, followed by a description of data collection and methodology. The results are then presented and discussed in detail. Finally, the conclusion presents key findings and suggests policy implementations while effectively acknowledging the study’s limitations.

## Literature review

### Conceptual background and theoretical framework

The study combines several theoretical frameworks to assess the multi-dimensional determinants of under-five mortality. DTP and CHE are based on the public health intervention model, implying a direct negative association with child mortality through the facilitation of quality healthcare. Economic development follows the Preston curve, expressing a non-linear relationship where initial investments in poor countries yield considerable gains, while returns diminish in wealthier nations. To statistically validate these diminishing returns, the model incorporates a squared formation of GDP. Complying with the theory of inequalities, significant wealth disparities among households are recognised to influence both the physical and mental health of family members [10].

**Fig. 2:**
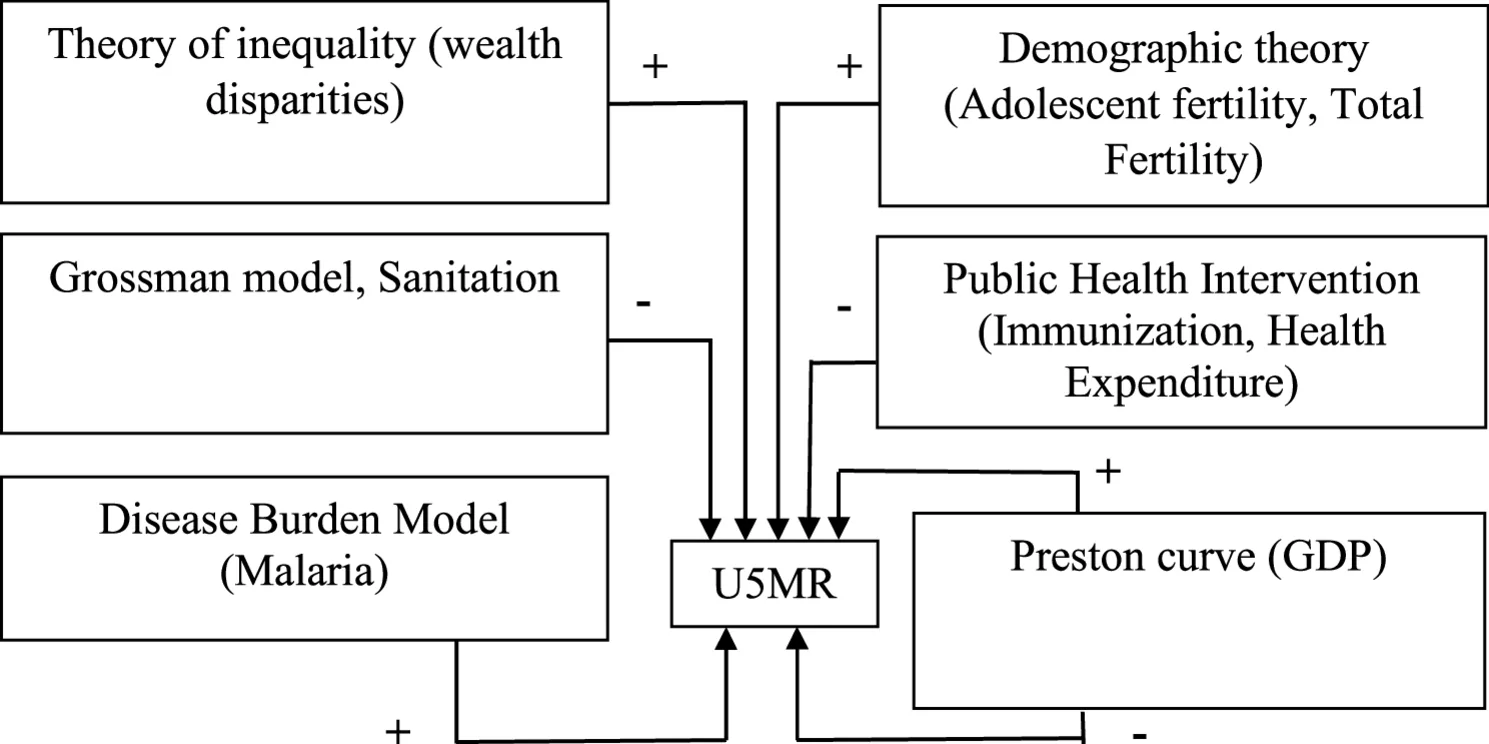
Theoretical framework of the study. Source: Authors’ illustration

IOM follows the disease burden model, affecting child survival through direct clinical consequences of cerebral malaria, anaemia [11] and indirect aspects like malnutrition. In accordance with the Grossman health production framework, a natural logarithm transformation is applied to BSS to capture potential non-linearity and diminishing marginal returns of infrastructure investments. Further, AFR and TFR reflect a nation’s socioeconomic and demographic profile. Since AFR exhibits both high and low associations on U5MR [12], both linear and squared terms have been used to capture the non-linear biological and social risks for adolescent pregnancies. The theoretical framework of the study is shown in Fig. 2.

The probability of a child born in a specific year or period dying before reaching their fifth birthday is defined as child mortality [2]. Even though child mortality shows considerable global reduction over time, it remains a critical global health challenge, mainly for African countries. When considering the African continent, only eight countries have reached the maximum reduction level of U5MR, which is 25 deaths per 1,000 live births, out of the 54 countries. Libya, Tunisia, Egypt, Morocco and Algeria from North Africa; Seychelles and Mauritius from East Africa; and Cabo Verde from West Africa are those eight countries and Sao Tome and Principe, South Africa, Rwanda, Botswana, Namibia and a few other East African countries are somewhat close to approaching the target, while all the other countries are far away from the objective [3]. Western Nigeria, Niger, Togo, Burkina Faso, and the Middle Eastern regions of Cameroon, Chad, and the Central African Republic can be considered as hotspots. Various determinants, such as the proportion of children immunised against DTP, IOM, GDP, CHE, AFR, TFR and BSS.

### Economic development and child mortality

Economic development, measured via GDP per capita, serves as a determinant of child survival due to its association with nutrition and healthcare accessibility. This relationship is theoretically grounded in the Preston curve, which proposes that income growth significantly reduces mortality in developing nations and these benefits show a diminishing return as nations become wealthier. Within the African region, empirical evidence shows that lower wealthy quintiles suffer from significantly higher child mortality [13]. Further, women from the poorest backgrounds frequently receive no Antenatal Care (ANC) compared to their more affluent counterparts [10, 14].

Increased national wealth is expected to have a statistically significant negative association with U5MR. However, a simple linear model of GDP is insufficient to effectively explain this relationship and a significant empirical gap exists in addressing the non-linear, lagged association of wealth in the African continent. This study addresses the gap by incorporating a delayed transmission mechanism, utilising a lagged quadratic specification (t-1) to account for the time required for national wealth to be manifest as improvements in health infrastructure for child survival outcomes.

- H1: Lagged GDP is associated with a statistically significant reduction in U5MR while its squared term reflects a diminishing marginal return.

### Immunisation and child mortality

DTP immunisation coverage is grounded on the public health intervention model, suggesting that vaccines are primary healthcare tools that reduce the risk of child mortality and morbidity by preventing critical illnesses and complications [15]. The spread of infectious diseases is reduced by facilitating herd immunity. Immunisation is expected to have a statistically significant negative association with under-five mortality [9]. This has been empirically observed in several contexts, such as Tunisia, where high coverage of vaccination provided a practical pathway for reducing U5MR [3].

However, deaths from preventable diseases remain unequal across the African continent [16]. Further, a significant challenge exists for the under-vaccination trend associated with multi-dose schedules [17]. Hence, facilitating immunisation programmes are important for the attainment of SDG 3.2 [18]. A critical empirical gap remains in assessing the effectiveness of DTP in reducing mortality disparities across both regional and country-specific settings. This study addresses the gap by revealing the regional association of DTP vaccination and national-level variations by providing an evidence base for targeted fund allocation.

- H2: Increased DTP coverage is associated with a statistically significant reduction in U5MR.

### Malaria burden and child mortality

Malaria incidence is a key determinant of under-five mortality [3], based on the disease burden model reflecting clinical complications such as cerebral malaria and severe anaemia directly affect child survival. Increased malaria incidence is expected to have a statistically significant positive association with U5MR. Evidence from the president’s malaria initiative in SSA demonstrates that targeted national-level precautionary steps can facilitate an accelerated decline in child mortality rates [19]. Furthermore, the implementation of post-discharge malaria chemoprevention and the integration of technological monitoring systems for larviciding [20, 21] underscore the strategic financial and health importance of investing in disease control [22] within highly endemic nations.

However, a significant empirical gap remains in identifying the association of malaria at the national level rather than depending on regional averages. Because the disease burden exhibits diversified trends across the African continent, due to nation-specific vulnerabilities. This study addresses this gap by assessing the regional association of the malaria burden with country-specific estimations to inform evidence-based interventions and local policy implications.

- H3: Increased IOM is associated with a statistically significant increase in U5MR.

### Health expenditure and governance

National investment in health as a share of GDP is referred as current health expenditure and is considered as a key indicator within the health production function. It is grounded in the public health intervention model and a statistically significant negative association with under-five mortality is expected [9, 23] by facilitating access to quality healthcare. Empirical evidence shows an inverse relationship between health expenditure and child or maternal mortality rates across various contexts. Targeted funding for healthcare services and education in Ghana is associated with considerable child survival gains [24].

However, the efficacy of health investments is usually moderated by the quality of national governance. While health expenditure is a key factor in the reduction of child deaths, corruption-free governance and institutional quality are recognised as crucial determinants for maximising its benefit. In the Nigerian context, control of corruption is considered as a key factors for contributing to the progress toward the SDGs by 2030 [25]. Furthermore, implementing programmes to improve air quality as a solution to several health issues in children [26] or address socio-economic inequalities in ANC [14] requires efficient governance to ensure the effective delivery of services. A critical empirical gap exists in evaluating the country-specific association of health expenditure and the delayed transmission mechanism of health investments. This study addresses this gap by utilising a lagged specification for CHE to account for the time interval required for budget allocations to manifest as tangible improvements in health infrastructure and paediatric outcomes.

- H4: Increased lagged CHE is associated with a statistically significant reduction in U5MR.

### Fertility dynamics and child mortality

Adolescent and total fertility rates serve as critical determinants of under-five mortality, based on demographic risk theories. These dynamics are expected to indicate a statistically significant positive association with U5MR due to the biological immaturity and socioeconomic constraints. Maternal age below 18 years is linked to significantly higher child mortality risks [18, 27] compared to the regional median delivery age of 26 years [28]. Western Africa shows susceptibility to these adolescent pregnancy risks [13, 29]. Early pregnancies often induce neonatal complications and congenital diseases because the mother is biologically immature. High parity (four or five prior children) [27] and birth intervals less than two years are linked with depletion of maternal nutrients and affecting birth weight and infant survival. Further, multiparous mothers experience specific challenges, rather than those faced by primiparous mothers [30, 31].

Evidence from Sierra Leone [32] and Tunisia [3] suggests that improvements in reproductive health, specifically through family planning and increased contraceptive prevalence, facilitate child survival by avoiding high-risk births [3, 33]. Hence, special attention needs to be given to vulnerable pregnancies and no ANC visits [34] to reduce the U5MR. However, a simple linear model is insufficient to capture these relationships, as biological and social constraints increase the risk non-linearly at higher levels of adolescent childbearing. A significant empirical gap exists in identifying national-level heterogeneity. This study addresses this gap by utilising a non-linear quadratic specification for AFR to capture the non-linear biological and social risks with adolescent pregnancies.

- H5: Increased fertility dynamics (AFR and TFR) are associated with a statistically significant increase in U5MR.

### Sanitation and environmental determinants

Basic sanitation services represent a fundamental environmental determinant for child survival, based on the Grossman health production framework linked with improvements in infrastructure to control the spread of infectious pathogens. Sustainable access to safe drinking water and basic sanitation is expected to exhibit a statistically significant negative association with the infant mortality rate [9]. The collective improvement of accessibility for clean water and sanitation with nutritional gains has led Tunisia to become a cold spot for child mortality within the African region [3], while unimproved sanitation and water cause child mortality in SSA [35].

However, existing knowledge indicates that children in areas with improved water sources may still face high mortality risks due to the negligence of quality testing, reflecting that simply providing access is insufficient [27]. Since there is a rising trend of diarrhoeal deaths among children under the age of five [36], responsible authorities need to prioritise the supply of clean water, sanitation, and hygiene infrastructure as a primary preventative measure against both water-borne and vector-borne diseases. A critical empirical gap exists in assessing the specific association of BSS on both a regional and national basis to ensure high returns on infrastructure investment. This study addresses this gap by utilising a non-linear logarithmic transformation of basic sanitation data to capture diminishing marginal associations across the considered 45 African nations, facilitating a contribution to the progress toward the SDGs.

- H6: Increased access to BSS is associated with a statistically significant reduction in U5MR.

### Research gap

Most prior studies on U5MR have focused on isolated factors, examining a limited set of variables over short timeframes. Consequently, a significant empirical gap exists in developing a multi-dimensional, holistic framework that accounts for the synergistic associations of economic, health-related, social, demographic, and infrastructural factors over an extended period. This study addresses this gap by utilising a comprehensive dataset spanning two decades (2000–2021) to capture long-term associations of considered key determinants.

Methodologically, this study controls for unobserved, time-invariant heterogeneity such as cultural norms and geographical characteristics which can lead to biased estimates. This study introduces methodological innovation via a Fixed-Effect (FE) panel model to identify the associations of specific determinants from these confounding factors. Furthermore, while the current literature rarely accounts for the delay transmission mechanism of national investments, this research incorporates lagged economic variables to measure the time required for budget allocations to manifest as health outcomes.

This study utilises Driscoll-Kraay robust estimates and year fixed-effects to ensure results are flexible to cross-sectional shocks and serial correlation. As regional averages often mask structural diversity, this study addresses a critical gap by providing descriptive, country-specific illustrations for 45 African nations. This approach facilitates the formulation of evidence-based, nation-specific policy interventions.

## Data and methodology

### Data sources and coverage

The study employed secondary data collected from the World Development Indicators (WDI) during the period 2000–2021. The balanced panel dataset is presented in Appendix 1. The rationale used for data collection is the availability of the published data in WDI. Missing data points were addressed by excluding countries with substantial data gaps. However, for very few remaining gaps within the selected 45 nations, standard linear interpolation was applied to maintain the integrity of the time-series. To assess potential selection bias, the comparison of included and excluded countries is presented in Appendix 2 reporting summary statistics of key variables. The results indicate a modest difference between the two groups without a clear systematic pattern. For instance, the included countries exhibit slightly higher average U5MR while excluded countries show somewhat higher GDP. These differences are relatively small, suggesting that the use of a balanced panel does not introduce substantial selection bias in the analysis. Furthermore, the consistency of regression results between the balanced and full sample supports the robustness of the findings and suggests that sample selection does not materially affect the estimated relationships and enhances transparency. To address concerns regarding sample selection bias, the complete 54- country dataset is provided in Appendix 3.

Moreover, this study was reported in accordance with the Strengthening the Reporting of Observational Studies in Epidemiology (STROBE) guidelines and thereby, transparency and completeness of the reporting was enhanced.

### Variable description and measurements

A description of each variable along with unit measures presented in Table 1. WDI codes for each variable are presented in Appendix 4.

**Table 1:** Operational definitions, measurement units, transformations and expected directional associations for variables used in the study of under-five mortality across 45 African countries (2000–2021)

Variable (WDI series Code)	Unit of Measure	Transformation	Expected sign
Dependent variable			
Under-five child mortality (U5MR) (SH.DYN.MORT)	per 1,000 live births	Level	
Independent variables			
GDP per capita (GDP) ( NY.GDP.PCAP.CD)	Current US $	Lag & Lag Squared	-
Immunisation for Diphtheria, Tetanus, Pertussid (DTP) ( SH.IMM.IDPT)	Share of one-year-olds vaccinated against diphtheria, tetanus and pertussis	Level	-
Incidence of Malaria (IOM) ( SH.MLR.INCD.P3)	The number of new cases of malaria in a year per 1,000 population at risk	Level	+
Current Health Expenditure (CHE) ( SH.XPD.CHEX.GD.ZS)	Current health expenditure % of GDP	Lag	-
Adolescent Fertility Rate (AFR) ( SP.ADO.TFRT)	number of births per 1,000 women ages 15–19	Level & squared	+
Total Fertility Rate (TFR) ( SP.DYN.TFRT.IN)	Total births per woman	Level	+
Basic Sanitation Services (BSS) ( SH.STA.BASS.ZS)	People using at least basic sanitation services (% of population)	Natural log (ln)	-

Source: Authors’ compilation

### Panel data structure

The balanced panel data set, spanning 22 years from 2000 to 2021 and focusing on 45 African regional countries, was used for the unit data analysis. This combines wide cross-sectional as well as time-series dimensions, facilitating a total of 990 country-year observations. This dual perspective uncovers the dynamics of considered determinants over two decades and is ultimately beneficial for suggesting practical policy implications.

### Economic model

Panel regression was applied as the analytical technique for the regional analysis, while MLR was utilised for the country-specific analysis in this study. This dual approach was employed because generalised regional associations may not apply to all nations equally due to structural heterogeneity and the lack of cross-sectional dimension at the individual country level. 

Equation 1 examined the associations of GDP, DTP, IOM, CHE, AFR, TFR, BSS on U5MR for African sample, while Eq. 2 represents the corresponding country-specific specification. In both models, i denotes the country and t denotes the year. The term $\:{v}_{i}\:$ captures the unobserved country-specific fixed effects,  represents the time fixed effect and $\varepsilon_{it}$ is the idiosyncratic error (disturbance term). U5MR is expressed at level (deaths per 1000 live births) while BSS enters the model in logarithmic form (level-log specification). To capture the delayed effect, the mitigate potential simultaneity bias lag values of GDP and CHE are used.     1

2 $$\begin{aligned}U5MR_{t}&=\beta_{0}+\beta_{1}GDP_{t-1}+\beta_{2}DTP_{t}+\beta_{3}IOM_{t}\\&+\beta_{4}CHE_{t-1}+\beta_{5}AFR_{t}+\beta_{6}{In}\left(BSS_{t}\right)\\&+\beta_{7}TFR_{t}+\beta_{8}GDP^2_{t-1}+\beta_{9}AFR^2_{t}+\varepsilon_{t}\end{aligned}$$

Equation 2 represents the country-specific specification of the empirical model. The equation is estimated separately for each country using a time-series of observations over the period 2000–2021, allowing the coefficients of the estimator variables to vary across countries. This approach enables the identification of country-level heterogeneity in the determinants of U5MR across the African continent.

The panel regression model facilitates the consistent estimation of the associations between the considered determinants. Furthermore, it offers significant benefits over cross-sectional or time-series analysis, particularly through its ability to control for unobserved heterogeneity [37]. Typically, cross-sectional analysis captures data at a single point in time, and thereby it bears the risk of omitting variables. Furthermore, failing to consider immeasurable and time-invariant factors, such as cultural, geographical, and historical norms, leads to biased estimates through cross-sectional analysis. The FE model of panel regression successfully overcomes these challenges by using within-country variation over time to estimate the links of the associating factors [38]. Hence, this panel model isolates the association of variables that change over time, leading to more reliable results. Thereby, practical policy implications can be suggested. Through a more informative data pool of multi-country and multi-year panel models, collinearity issues are reduced among the variables [37], implying that it is the most efficient and powerful analytical model for the analysis.

### Diagnostic and robustness test

The stationarity properties of the panel variables were examined using the Levin-Lin-Chu (LLC) unit root test. Multicollinearity was assessed using both the correlation matrix and variance inflation factor (VIF). To determine the appropriate estimator, the Hausman specification test was employed to compare FE and RE models. Statistically significant Hausman test statistics support the FE estimator with the rejection of the null hypothesis, indicating that unobserved country-specific associations are correlated with the regressors. Accordingly, the FE approach was adopted, as it controls for time-invariant country heterogeneity and mitigates omitted variable bias arising from unobserved structural differences across countries.

The fixed effect regression estimates were reported using heteroscedasticity robust standard errors clustered at the country level which account for potential serial correlation and heteroscedasticity within countries across time. Clustering at the country level is recommended for the panel dataset where observations within the same country may exhibit correlated disturbance. To further ensure the robustness of the findings, additional sensitivity analyses were conducted. First, the model was re-estimated using Driscoll-Kraay standard errors, which are robust to heteroscedasticity, serial correlation and sectional dependency across panels. Second, year fixed effects were included to control for common time-specific shocks affecting all countries, such as global health initiatives or macroeconomic fluctuations. While the primary specifications focus on country-level fixed-effects to control for unobserved heterogeneity, year fixed-effects are included separately as a robustness check. The results remain broadly consistent across these alternatives, specification confirming the stability of the estimated relationship.

Moreover, descriptive summary statistics were compiled and MLR analyses were conducted. The software Stata 17 was used for the study, while Python was used for the graphical presentation of the heat map.

Given the relatively short time dimension of the country-level regressions, the country-specific models were estimated using heteroscedasticity and autocorrelation consistent HAC standard errors, based on the Newey-West estimator, to account for potential serial correlation and small sample uncertainty. The application of HAC standard errors in Eq. 2 is specifically intended to mitigate the risks associated with small sample uncertainty. The limited time dimension (T = 22) and the large number of country-specific regressions at the individual country level is intended to be illustrative and descriptive rather than inferential, as multiple testing may increase the likelihood of superior statistical significance. It restricts the statistical power and strength of the conclusions compared to the regional panel analysis. Hence, results need to be interpreted with caution due to potential multiple testing issues and small sample uncertainty.

### Methodological workflow

**Fig. 3:**
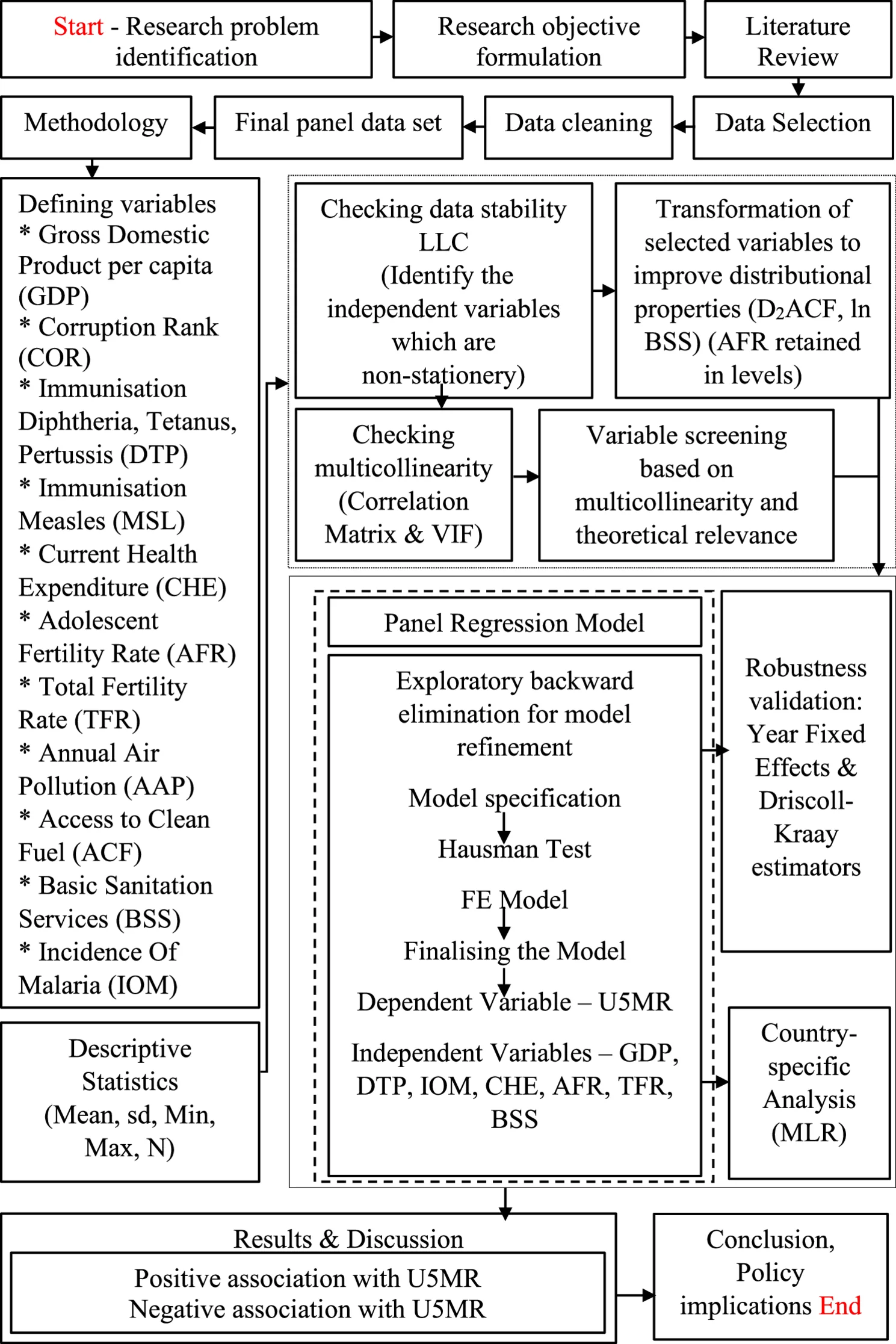
Methodological workflow for the analysis of under-five mortality determinants across 45 African nations (2000–2021). Note: U5MR: Under-five mortality rate; LLC: Levin-Lin-Chu unit root test; VIF: Variance Inflation Factor; MLR: Multiple Linear Regression; FE: Fixed-Effects model; GDP: Gross Domestic Product per capita; DTP: Diphtheria-Tetanus-Pertussis immunisation; IOM: Incidence of Malaria; CHE: Current Health Expenditure; AFR: Adolescent Fertility Rate; TFR: Total Fertility Rate; BSS: Basic Sanitation Services; COR: Corruption Rank; MSL: Measles immunization; AAP: Annual Air Pollution; ACF: Access to Clean Fuel; D2ACF: second difference of the Access to Clean Fuels and technologies for cooking. Source: Authors’ compilation

Descriptive statistics were tested for all the considered variables (results presented in Appendix 5). The panel unit root test (LLC) indicates (results are presented in Appendix 6) that most variables are stationary in level, while AFR and BSS exhibit non-stationarity. No transformation was applied to AFR and it is retained in level form due to its strong theoretical relevance and its role in capturing non-linear demographic associations through the inclusion of a squared term. Variables such as GDP and BSS are included in lagged and logarithmic forms respectively, to apply delayed transmission mechanism and improve distributional properties. Multicollinearity was tested using the correlation matrix and VIF. The results of these tests are presented in Appendix 7. VIF results indicate relatively higher values for the AFR and GDP variables, particularly due to the inclusion of squared terms. This is expected as quadratic specifications introduced inherent multicollinearity. These variables are retained based on strong theoretical consideration as they capture a non-linear relationship in demographic dynamics and economic development. While multicollinearity may increase standard errors and reduce coefficient precision, it does not introduce biases into the estimate. Therefore, results are interpreted with appropriate coefficients. A stepwise backward elimination procedure was employed as an exploratory model refinement approach. Given the limitation of the stepwise selection, the full candidate-set model, including all theoretically relevant variables, is also reported ensuring transparency and allow comparison between specifications. Following that, the Hausman test was conducted with the final set of variables, and the FE model was selected, with the rejection of the null hypothesis and results were obtained using heteroscedasticity robust standard errors clustered at the country level. Results of these tests are presented in Appendix 8. Parallelly, MLR was conducted to analyse the country-specific association of each variable. A flow chart of the methodology is presented in Fig. 3.

## Results

### Descriptive statistics and trends

**Fig. 4:**
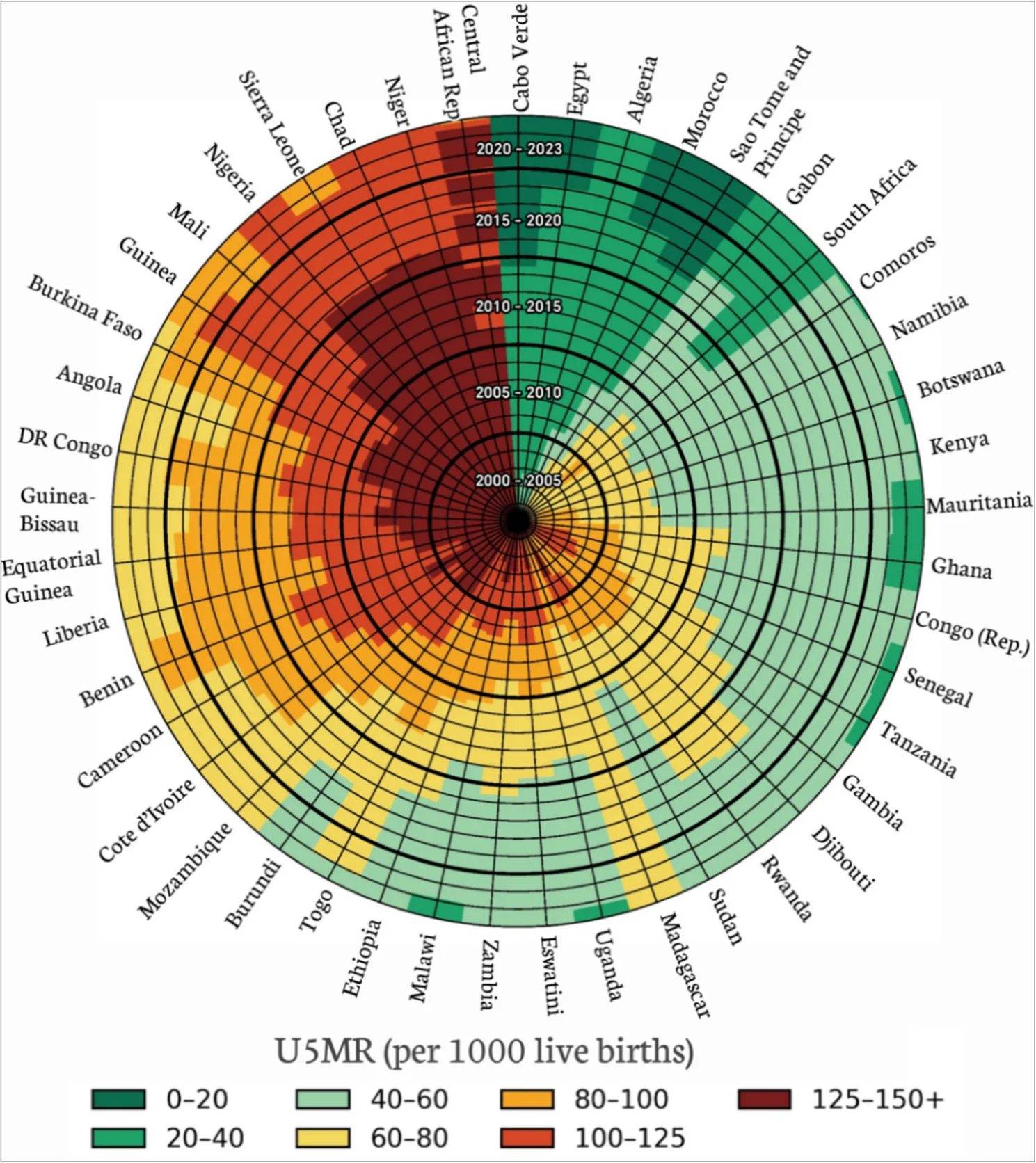
Changes in under-five mortality rates (per 1,000 live births) across 45 African countries (2000–2023). Note: Polar heat map of Under-five mortality rate (U5MR) per 1000 live births in 45 African Regional countries during the period 2000-2023. Each ring represents a calendar year. Countries are arranged clockwise according to the average mortality over the period. The colour scale represents the severity of U5MR. Dark green represents the lowest, and dark red represents the highest. Source: Authors’ illustration

The highest U5MR is exhibited in the Central African Republic, Sierra Leone, and Niger, while the lowest is recorded in Cabo Verde, Algeria, and Egypt. The highest rate for immunisation of DTP records is from Morocco and Egypt, while the lowest record is from Chad and Central Africa, suggesting the link between quality healthcare on U5MR. Similarly, the lowest rate for IOM is exhibited in Egypt. Equatorial Guinea shows the highest GDP per capita, in contrast with the lowest CHE, with a considerable U5MR of 112. This can be attributed to a governance failure, as it ranks as the 3rd most corrupt country. Niger has recorded the highest rate for both TFR and AFR of 7.37 and 175.54, respectively. The highest rate for BSS records is in Egypt, while the lowest value records are from Ethiopia. The challenges for child survival in a particular country are interlinked, as most countries with high mortality are affected by multiple variables, such as low coverage of DTP immunisation, high fertility, high malaria burden, and vice versa. In Nigeria, for example, the country has low coverage of DTP immunisation, high fertility rates, and a high malaria burden. In contrast, in Cabo Verde, the situation is reversed. Changes in U5MR in the African region from 2000 to 2023 are illustrated in Fig. 4.

The country-specific regressions highlighted heterogeneity across countries. However, these results should be interpreted as descriptive patterns rather than formal statistical inference. The most consistent association is for BSS being negative. Burundi and Cameroon show a considerable negative association of BSS with U5MR, exhibiting coefficients of -1139.74 and --653.68, respectively. Focusing on the poorest country, such as Burundi, shows a significant association between the reduction in U5MR and increased wealth, while Algeria, a middle-income country, exhibits a diminishing return. This implies the relationship between the Preston curve and GDP.

### Panel regression results (regional analysis)

The comprehensive results for the regional fixed-effect model are detailed in Table 2. The final stepwise model utilises 945 observations and incorporates heteroscedasticity robust standard errors clustered at the country level. The inclusion of the full model ensures that results are not driven solely by the selection procedure and provides a robustness benchmark for interpretation.

**Table 2:** Regional determinants of under-five mortality in 45 African countries; results from the baseline fixed-effects panel regression model (2000–2021)

Variable	Coefficient	Robust std. error	*P* value
lag GDP	-0.0071^***^	0.0018	0.000
	(-3.93)		
DTP	-0.4540^***^	0.1086	0.000
	(-4.18)		
IOM	0.0518^**^	0.0248	0.043
	(2.09)		
lag CHE	-0.7744	0.5994	0.203
	(-1.29)		
AFR	-0.7524^**^	0.3378	0.031
	(-2.23)		
TFR	29.7570^***^	6.8200	0.000
	(4.36)		
ln BSS	-19.0198^***^	6.8000	0.008
	(-2.80)		
lag GDP^2^	0.3120 × 10^− 6***^	9.1700 × 10^− 8^	0.001
	(4.46)		
AFR^2^	0.0022*	3.1600	0.001
	(3.40)		
constant	95.8578**	39.7220	0.020
	(2.41)		
*R*^2^ Within	0.6670		
*R*^2^ Between	0.6186		
*R*^2^ Overall	0.5959		
*N*	945		

***p < 0.01, **p < 0.05, *p < 0.1 and *t* statistics in parentheses

*GDP *Gross Domestic Product per capita (lagged), *DTP *Diphtheria-Tetanus-Pertussis immunisation, *IOM *Incidence of Malaria, *CHE *Current Health Expenditure (lagged), *AFR *Adolescent Fertility Rate, *TFR *Total Fertility Rate, *ln BSS *Natural log of Basic Sanitation Services access

Source: Authors’ compilation

Using panel regression analysis, this section aims to establish the association of GDP, DTP, IOM, CHE, AFR, TFR and BSS on U5MR. According to the result of the FE model shown in Table 2, lag GDP, immunisation of DTP and BSS exhibit a negative association with U5MR with coefficients of -0.0071 (*p* = 0.000), -0.4540 (*p* = 0.000) and − 19.0198 (*p* = 0.008). In contrast with IOM, TFR and higher values of AFR show a statistically significant positive association with U5MR with coefficients of 0.0518 (*p* = 0.043), 29.7570 (*p* = 0.000) and 0.0022 (*p* = 0.001) at a statistically significant confidence level. TFR has shown a highly statistically significant positive relationship with U5MR. The quadratic terms of GDP and AFR reveal non-linear associations with child mortality. The model fit statistics (*R*^2^ within: 0.6938) confirm that the selected holistic framework effectively captures the associated factors for U5MR in African nations. The preferred specification is derived through an explanatory refinement process. However, the full model results are also presented in Appendix 8 to ensure transparency and robustness of interpretation.

A potential concern in cross-country mortality analysis is persistence in baseline outcome. In our model, country fixed effects are included to control for unobserved time-invariant heterogeneity. Importantly, the estimated coefficients reflect within-country temporal effects and therefore capture whether improvement in GDP, health spending, vaccination coverage and sanitation is associated with reductions in U5MR overtime even in countries with structurally high initial mortality.

The FE regression estimates were reported using heteroscedasticity robust standard errors clustered at the country level, which account for potential serial correlation and heteroscedasticity within the countries over time. Table 3 shows the comparison of main FE results with year fixed effects and Driscoll-Kraay robustness checks. The results are included in Appendix 9.

**Table 3:** Robustness validation for determinants of under-five mortality in Africa: Comparison of fixed-effects models with year effects and Driscoll-Kraay standard errors (2000–2021)

Model	FE clustered SE	FE + Year FE	FE Driscoll - Kraay
lag GDP	-0.0071	0.0022	-0.0071
DTP	-0.4540	-0.2648	-0.4540
IOM	0.0518	0.0266	0.0518
lag CHE	-0.7744	-0.3203	-0.7744
AFR	-0.7524	-0.9756	-0.7524
TFR	29.7570	21.0511	29.7570
ln BSS	-19.0198	-4.9179	-19.0198
lag GDP^2^	0.3120 × 10^− 6^	-0.0990 × ^10−6^	0.3120 × 10^− 6^
AFR^2^	0.0022	0.0030	0.0022

The robustness checks that estimated associations remain broadly stable across alternative standard errors specifications

Source: Authors’ compilation

Robustness analysis including year fixed effects confirms that only DTP immunisation and TFR remain robustly significant determinants. The loss of statistical significance for malaria incidence, sanitation and economic indicators (lag GDP and CHE) indicates that these associations are highly specification-sensitive and should be interpreted with caution as their associations may be partially captured by broader temporal dynamics in regional development. To further ensure the robustness of the findings, the model was re-estimated using the full sample of 54 African nations (*N* = 976). The results obtained from the full sample are consistent with the primary 45-country balanced panel, confirming that the identified associations are not sensitive to the exclusion of nations with missing data points.

Although the FE model has identified significant relationships between the independent variables and U5MR on a continent-wise basis, as it is not applicable to all countries, a country-specific analysis of each selected independent variable is presented in Appendix 10.

## Discussion

### Economic development and inequality

Since GDP per capita shows a non-linear association on U5MR, the lag version of both GDP and its squared version have been considered in the analysis. Both lagged versions of GDP and squared GDP were statistically significant, with coefficients of -0.0071 and 0.3120 × 10^− 6^ respectively. It suggests that the association with U5MR depends on the level of economic development of a nation. At lower economic stages, a $1,000 increase in GDP per capita in the previous year is associated with a reduction of approximately seven deaths. This finding is consistent with the results of [39] in the South Asian context and [13] in the African context. In contrast, as results revealed from the study, this benefit diminishes for the countries having a higher economic development, validating the application of the Preston curve and the Grossman model. It confirms that while initial income growth is crucial, increased wealth in stable African nations results in a diminishing pattern of return regarding child mortality reduction. It reflects the necessity of health equity as suggested by [10], uncovering the associations of wealth inequality in child mortality in Togo. The lagged GDP in the final economic model in the study reflects the delayed transmission mechanism, confirming that GDP requires a time gap to benefit in the healthcare setting of a country.

Complying with the average negative association of lag GDP per capita on U5MR, the MLR result showed that in Burundi, a $1000 increase of GDP in the previous year is associated with a decrease of 337 child deaths in the current year. The underlying cause for this relationship would be the probability of funding each additional dollar for essential health services in the country more effectively than in a wealthier nation. Similar trends were observed in other countries, such as Niger, Rwanda and Malawi, indicating a high return to growth. Further, when countries are in the trend of increasing their GDP from a low level, they can allocate more funds for health, such as basic immunisation programmes, and increase their expenditure on health. However, the sensitivity of the GDP coefficient to year fixed effects suggests that the economic growth association is closely tied to specific temporal periods of regional expansion.

### Health system and public health interventions

Regional FE analysis demonstrates that DTP immunisation has a robust negative association with U5MR (coefficient of -0.454, *p* < 0.001). This implies that a 1% point increase in DTP coverage is associated with 0.45 fewer child deaths per 1000 live births. This finding is further validated by the Driscoll-Kraay robust estimator, where the coefficient and significance remain unchanged, suggesting the importance of a country’s primary health system. Several countries follow this trend. This finding aligns with the previous literature of [15, 18], which emphasised the importance of facilitating immunisation programmes and ensuring that all children receive the essential vaccinations to contribute to SDG 3.2. In Rwanda, it has been demonstrated that a 1% increase in DTP vaccination is associated with a decrease of approximately 0.9 child deaths per 1000 live births. This association reflects the effectiveness of the immunisation programme against fatal diseases in children. Vaccination against DTP is a significant approach in child health, as diseases like whooping cough are detrimental to children. This measurement is not just a statistic, and it can be considered a characteristic of functional primary healthcare. In such a healthcare setting, which can successfully implement an immunisation programme for three doses of a vaccine over a period of several months, it demonstrates the capability of providing close monitoring of the child’s health, including growth, nutrition, and other essential health services. This trend is followed by countries such as Malawi, São Tomé and Principe, and Guinea, which demonstrate high effectiveness in child survival.

Algeria, which has a low average U5MR of 29.5, maintains a comparatively high average DTP immunisation rate of 90.7%, in contrast to Angola, which has a high U5MR of 125 and a relatively low immunisation rate of 50.3%. Consistent with the common regional results, existing knowledge of [3] also confirmed that immunisation coverage is an important key determinant in U5MR.

In the African region, a 1% increase in health expenditure as a share of GDP in the previous year is linked with a reduction of U5MR by nearly 0.8 deaths per 1,000 live births in the current year. However, it did not reach statistical significance (*p* = 0.203) as an independent predictor in the final regional specification. This suggests that simply increasing the share of GDP spent on health may not be sufficient for child survival without continuous improvements in governance. Aligning with this finding, existing literature of [40] confirmed the relationship between U5MR and institutional quality in SSA countries. Gambia has demonstrated a return on investment for CHE, indicating a 1% increase in lagged CHE associated with the reduction of 6.64 deaths per 1000 live births. As a country with substantial capacity in health settings, this additional expenditure is utilised for Anti-Retroviral Therapy (ART) to minimise the transmission of HIV from mother to child, linked to a considerable reduction in child deaths. The negative association between CHE and U5MR highlights the importance of strengthening the health system by funding the health sector to reduce U5MR. In line with the study results, existing knowledge has also demonstrated a negative correlation between child and maternal mortality and health expenditure [25]. Further, in accordance with [41], health expenditure is a vital determinant in the improvement of health status in a country. In compliance with the same trend, Rwanda and Algeria reveal significant negative coefficients, reflecting a high associative return.

The access to basic sanitation services (ln BSS) exhibits a significant negative association with child mortality (coefficient of -19.02, *p* = 0.008). Analytically, a 1% increase in the proportion of the population with BSS access is associated with a decrease of 19 deaths per 1000 live births. However, this coefficient loses significance when year fixed-effects are included, suggesting that the association may be partially captured by broader temporal trends in regional infrastructure development. The profound association in Burundi, reducing approximately 11 child deaths to a 1% increase in BSS in the proportion of the population, might be due to the massive health gain resulting from improving basic sanitary requirements from low coverage. Similarly, almost all countries exhibit this negative trend of BSS on U5MR, emphasising its universal importance. Diarrhoeal diseases, which are due to pathogens like *cholera* and *E. coli*, are fatal for children under 5, as they might link to severe conditions such as dehydration and malnutrition. With the accessibility of BSS, the contamination of food and water by faecal materials is significantly reduced, disrupting the primary transmission routes for pathogens and contributing to a healthier nation. In accordance, prior knowledge of [35] reveals that the disrupting of transmission of pathogens is crucial, the same as medical interventions. Moreover, the existing literature has also confirmed the negative association of BSS with U5MR [3, 9]. Hence, it has been proven that the importance of budget allocation in improving the country’s infrastructure for child survival is evident. It is important to note that the statistical significance of sanitation access is sensitive to model specification, indicating that its association is likely intertwined with broader infrastructure trends over time. However, the association of DTP, CHE and BSS collectively indicates the association of the public health intervention model on U5MR.

### Disease burden and child survival

The IOM is positively associated with U5MR (coefficient of 0.052, *p* = 0.043). Specifically, every one-unit increase in malaria cases per 1000 people at risk is associated with an increase of 0.05 child deaths. This result is particularly pronounced in country-specific descriptive models, such as in Namibia (coefficient of 0.14, *p* < 0.001), where the magnitude is nearly triple the regional average. These findings highlight the importance of intervening in community health control programmes. Country-specific results suggest variation in the relationship between malaria and U5MR across countries. The positive association gained from the study findings is aligned with the framework which is discussed in prior literature of [11]. Malaria may be fatal for children due to cerebral malaria, severe anaemic conditions, respiratory distress, as a direct link and the probability of weakening the immunity system of children and making them more susceptible to pneumonia too, as indirect associations. Low birth weights are also expected for mothers who had to suffer from malaria during their pregnancy. Since these conditions ultimately contribute to higher U5MR in a country, investing in malaria control programmes is important for high-endemic countries. Consistent with the statistical analysis, it has been identified that the elimination of malaria as a prominent factor for child mortality has been identified through previous studies as well [3, 22]. Further, in line with [42], access to improved sanitation and water has been identified as significantly associated with U5MR and complies with the study findings. Therefore, controlling the spread of endemic diseases needs to be addressed by susceptible countries to reduce child deaths and critical complications in childhood as well. Even though IOM and BSS exhibit statistically significant associations in the baseline fixed-effect model, these relationships are not robust to the inclusion of a year fixed effect, indicating sensitivity to time-specific dynamics. Further, the sensitivity of malaria incidence to year fixed effects highlights that its association with child mortality is context-dependent and subject to common time-specific health shocks across the continent.

### Demographic factors

Demographic factors exhibit the highest magnitudes of association; TFR shows a highly significant positive association (coefficient of 29.76, *p* < 0.001). This indicates that one additional birth per woman is associated with approximately 30 additional child deaths per 1000 live births. Similarly, the non-linear biological risks of early childbearing are captured by the AFR squared term (coefficient of 0.0022, *p* = 0.001), showing that the mortality risk intensifies as adolescent fertility rates increase. These findings propose the importance of family planning and the link between female education and awareness. Further, the association between AFR and child mortality is level-dependent. The quadratic specification of AFR suggests that the increasing of child mortality intensifies at higher levels of childhood pregnancies. As a key factor in the model, the fertility rate has a highly positive magnitude and a statistically significant association on U5MR. Sudan exhibits a descriptive relationship of TFR towards U5MR. Since Morocco has already realised a significant reduction in U5MR by controlling primary risk factors, this demographic factor has become more prominent and can be identified as the final barrier for a healthy nation. In accordance with the study results, existing literature of [13] teenage mothers bear higher risk of U5MR.

When birth spaces are too short, it affects underweight child births, as there is no adequate time for restoring essential nutrients like iron and folate. The highly detrimental positive association of AFR with U5MR is evident in Mozambique, where an increase of one birth per 1000 adolescent women is associated with an increase of16 deaths in the U5MR. This significant positive relationship corresponded to both biological and social threats of having early-age births, making the children more vulnerable to childhood deaths. Since the bodies of adolescent mothers are not fully developed biologically for childbirth, it might lead to several complications in the child, like low birth weight and pre-term births. Low awareness of prenatal and postnatal care also significantly affects this positive trend. Similarly, previous studies have revealed multiparity and short birth intervals are high-risk factors [31] and proved the risk of adolescent pregnancy towards U5MR [27]. However, it is required to isolate the association of fertility from the cultural and social norms of a country and analyse its association to avoid bias.

## Conclusions

### Summary of the key findings

This study examines the measures for U5MR on the African continent and their country-specific associations, based on 45 nations over a two-decade period. The study identifies DTP immunisation and TFR as the most robust regional factors associated with under-five mortality across African countries. While lag GDP, access to sanitation (ln BSS), and malaria incidence (IOM) exhibit significant associations in baseline specifications, these findings are specification-sensitive. This indicates that their role as child mortality determinants is context-dependent and influenced by broader temporal dynamics, requiring policy interventions that account for both regional trends and nation-specific structural diversity. Therefore, findings should be interpreted as context-dependent rather than universally stable across specifications.

TFR has demonstrated its core association with child survival, highlighting the importance of birth order, family planning, and overcoming the unique challenges faced by multiparous mothers in a nationwide context. These findings disclose that the sustained economic growth of a country affects child survival in terms of increasing expenses on health settings through uplifting quality community health, like immunisation programmes, investing in public health infrastructure and improving access to sanitation and controlling endemic diseases such as malaria in high-susceptible countries, which can be detrimental to children directly as well as incidentally. On the other hand, addressing demographic challenges like fertility rates, child marriages, and early pregnancies is vital for a notable reduction in child mortality rates across the African region.

Country-specific MLR results generally align with the average regional trend for most countries, while some countries exhibit results that differ. Hence, policy implementation required focusing on each country specifically rather than on a continental basis. However, these country-specific results are primarily illustrative and descriptive. These models are intended to showcase potential national deviations rather than to serve as standalone inferential proofs.

### Policy and practical implications

While the following recommendations incorporate insights from both regional and national models, it is important to emphasise that country-specific findings remain descriptive in nature. These results are provided to illustrate heterogeneity across the panel and should be used to supplement, rather than replace, regional inferential evidence when formulating localised health strategies. Priority is given to interventions supported by robust regional coefficients, while national-level suggestions serve as descriptive guides for potential localised health strategies.

As indicated by the regional analysis, DTP immunisation reflects a considerable association with child survival. A 1% point increase in coverage is associated with a reduction of approximately 0.45 child deaths per 1,000 live births (coefficient of -0.45, *p* < 0.01) and therefore, improving the vaccine coverage in high-risk countries is suggested by the study results as a regional priority. At the national level, the ministries of health in countries with persistently low means of immunisation coverage, such as Chad, the Central African Republic and Nigeria, are suggested to prioritise launching national immunisation programmes routinely to ensure completion of the three doses of the DTP vaccine schedule. For nations like Rwanda, where the descriptive country-specific coefficient indicates an even more pronounced association (coefficient of -0.9, *p* < 0.05), a targeted 5% annual increase in coverage is proposed to facilitate rapid progress toward SDG 3.2. Given that a unit increase in malaria incidence is associated with a 0.0518-point rise in U5MR regionally and is particularly high in endemic nations like Namibia with country-specific coefficient of 0.159, it shows that every unit increase in malaria incidence contributes for a considerable threat to child survival and therefore, preventative measures are suggested to control the malaria burden. In addition to that, for countries like Burkina Faso, Benin and Sierra Leone, where malaria continues, precautionary steps can be taken by implementing malaria control programmes, distributing insecticide-treated bed nets for pregnant women and children and facilitating public hospital settings with rapid diagnostic tests and artemisinin-based combination therapy. Malaria control remains an important policy area. However, given that its statistical significance is sensitive to model specification, policy recommendations need to be considered alongside country-specific conditions and temporal dynamics.

Empowering community health settings and health workers, including training and ensuring the availability of medicines in rural areas, as well as conducting awareness programmes for reproductive health, would be beneficial for South Africa as a middle-income country that has significant capacity but struggles due to inequalities. Increasing health expenditure and investing in building quality community health would be worthwhile for countries like Equatorial Guinea and the Republic of Congo, which are having low expenses on health, in reaching their SDG 3.2.

Furthermore, ministries of education need to implement policies to improve girls’ education by enhancing enrolment in secondary education, including lessons on reproductive health in the basic education curriculum, and enforcing laws against child marriage as long-term precautions. This would be important in most susceptible countries such as Niger, Equatorial Guinea and Chad, which have high mean rates of adolescent pregnancies. The regional results indicate that a one-unit increase in the total fertility rate is associated with an increase of approximately 30 additional child deaths per 1,000 live births (coefficient of 29.7570, *p* < 0.01). This underscores the suggested expansion of modern family planning services, particularly in nations like Morocco, where the descriptive national association is comparably higher (coefficient of 65.0), to mitigate biological and socioeconomic risks to child survival. Given the link between fertility rates of a country and child mortality, Niger and Chad exhibit high fertility rates and child deaths. Therefore, the ministries of health in those countries are suggested to facilitate widespread modern family planning services, ensuring accessibility for all. Study results suggest that there is a significant association with sanitation services and a reduction of child deaths in the African continent, with a regional coefficient of -19.02. Hence, investing in new infrastructure projects to improve sanitation services, particularly where there is low coverage of BSS, would be beneficial for those nations that are susceptible for a high rate of child deaths. In nations showing a strong descriptive association, such as Burundi (national coefficient of -11.0), prioritising investment in new sanitation infrastructure is proposed. Furthermore, budget allocation for the health sector, which minimises the heterogeneity of health access within the country, is also necessary for highly susceptible countries such as Chad and Niger. In addition to that, on the international level, the WHO and UNICEF may contribute by tailoring funding for child-saving programmes in the required countries, including Burundi, the Democratic Republic of Congo, the Central African Republic, Ethiopia, and Niger, which have the lowest GDPs. It would be beneficial for eliminating preventable child deaths by 2030.

### Theoretical and academic contribution

This study contributes to the existing knowledge of literature by identifying regional factors associated with child survival. Methodologically, statistical results are based on a fixed-effects panel model for 45 countries, which isolates the considered determinants from unobserved, country-specific factors that are not feasible to control through a single-country or cross-sectional country analysis. Furthermore, this study employs a multidimensional, holistic framework over 22 years. Moreover, the non-linear nature of the Preston curve and the diminishing returns of income have been empirically supported through the significant quadratic terms in the lagged economic model.

### Limitations and further research directions

Limitations of the study include the availability of secondary data only for some independent variables and countries, as well as the inability to eliminate potential sources of endogeneity, such as time-varying omitted variables. Although AFR is not stationary in levels, it is retained based on theoretical considerations. This may introduce some estimation sensitivity and results need to be interpreted with caution. Although a balanced panel improves comparability over time, it may exclude countries with incomplete data, which would affect representativeness. Further, the multiple testing of 45 nations and short time dimension (T = 22) limit the inferential strength of individual country results of multiple linear regression.

Furthermore, the sensitivity of several key variables like malaria, sanitation, and GDP to model specification considering the year fixed effects is a notable limitation. This indicates that the regional panel results for these determinants may be partially confounded by unobserved common temporal trends. Hence, these findings need to be interpreted as suggestive components.

As U5MR is a non-negative outcome variable, a purely linear specification may theoretically yield negative fitted values outside the observed covariant range. To address this concern, the study additionally estimated the log-linear estimator (ln U5MR) as a robustness check. Future research may further explore alternative bounded outcome models such as Poisson pseudo-maximum likelihood with fixed effect. Future research needs to explore the association of country-specific cultural practices in African regional nations with child mortality over time. Quantitative analysis would effectively examine the relationship between maternal health, immunisation, and other sociodemographic factors and child mortality. Thereby, responsible authorities can focus more on policy implications with respect to the different regions in a country.

## Supplementary Information

Supplementary Material 1: Appendix 1: Balanced Panel dataset.

Supplementary Material 2: Appendix 2: Comparative analysis of sample selection (Descriptive summary statistics and model sensitivity for primary balanced panel and full dataset).

Supplementary Material 3: Appendix 3: Complete 54-Country Dataset (Including excluded nations).

Supplementary Material 4: Appendix 4: World Development Indicators codes for each variable.

Supplementary Material 5: Appendix 5: Descriptive summary statistics.

Supplementary Material 6: Appendix 6: Assessment of stationarity properties for under-five mortality and its determinants across 45 African nations using Levin-Lin-Chu unit root tests (2000–2021).

Supplementary Material 7: Appendix 7: Diagnostics for multicollinearity among determinants of under-five mortality across 45 African nations: Pairwise correlation matrix and Variance Inflation Factor results (2000–2021).

Supplementary Material 8: Appendix 8: Regional panel regression results for the full candidate-set of potential determinants of under-five mortality across 45 African nations: Stepwise backward elimination process and model specification diagnostics (2000–2021).

Supplementary Material 9: Appendix 9: Robustness and sensitivity analysis for regional determinants of under-five mortality across 45 African nations: Fixed-effects models with integrated year effects and Driscoll-Kraay robust standard errors (2000–2021).

Supplementary Material 10: Appendix 10: Country-specific analysis (Descriptive illustrations).

## Data Availability

All data generated or analysed during this study are publicly available and included in this published article and its supplementary information files.

## References

[1] Avelino IC, Van-Dúnem J, Varandas L. Under-five mortality and social determinants in africa: a systematic review. Eur J Pediatr. 2025;184(2):150. 39849277 10.1007/s00431-024-05966-w PMC11759474

[2] Child mortality and causes of death. [https://www.who.int/data/gho/data/themes/topics/topic-details/GHO/child-mortality-and-causes-of-death].

[3] Adedini SA, Anjorin SS, Mobolaji JW, Okolie EA, Yaya S. Assessing Africa’s child survival gains and prospects for attaining SDG target on child mortality. PLOS Glob Public Health. 2024;4(7):e0003022. 38985728 10.1371/journal.pgph.0003022 PMC11236144

[4] Sharrow D, Hug L, You D, Alkema L, Black R, Cousens S, Croft T, Gaigbe-Togbe V, Gerland P, Guillot M, et al. Global, regional, and national trends in under-5 mortality between 1990 and 2019 with scenario-based projections until 2030: a systematic analysis by the UN Inter-agency Group for Child Mortality Estimation. Lancet Glob Health. 2022;10(2):e195–206. 35063111 10.1016/S2214-109X(21)00515-5 PMC8789561

[5] Verhulst A, Prieto JR, Alam N, Eilerts-Spinelli H, Erchick DJ, Gerland P, Katz J, Lankoande B, Liu L, Pison G, et al. Divergent age patterns of under-5 mortality in south Asia and sub-Saharan Africa: a modelling study. Lancet Glob Health. 2022;10(11):e1566–74. 36088913 10.1016/S2214-109X(22)00337-0 PMC9588693

[6] Geographies. in Depth Thought leadership, analysis and solutions on the world’s biggest challenges. [https://www.weforum.org/stories/geographies-in-depth/].

[7] Research Highlights. in 2024. [https://www.nature.com/natafrica/articles?type=research-highlight&year=2024].

[8] Child. mortality rate, ages 0–4. [https://ourworldindata.org/grapher/child-mortality-around-the-world].

[9] Ester PV, Torres A, Freire JM, Hernández V, Gil Á. Factors associated to infant mortality in Sub-Saharan Africa. J Public Health Afr. 2011;2(2):e27. 28299068 10.4081/jphia.2011.e27 PMC5345500

[10] Pelenguei E, Pilo M. Effect of wealth inequality on child and infant mortality in Togo. BMC Health Serv Res. 2022;22(1):1499. 36482465 10.1186/s12913-022-08912-4 PMC9733086

[11] Asante KP, Bozonnat MC, Savic M, Owusu-Agyei S, Kaali S, Otieno W, Boahen O, Tivura M, Otieno L, Agyapong PD, et al. Incidence rates of malaria, meningitis, and mortality in children younger than 5 years: a prospective cohort study in Ghana and Kenya before the roll-out of the RTS,S/AS01(E) malaria vaccine from 2016 to 2022. Lancet Glob Health. 2025;13(5):e859–69. 40288396 10.1016/S2214-109X(25)00022-1

[12] Geronimus AT, Korenman S. The Socioeconomic Consequences of Teen Childbearing Reconsidered*. Q J Econ. 1992;107(4):1187–214.

[13] Andegiorgish AK, Woldu HG, Elhoumed M, Zhu Z, Zeng L. Trends of under-five mortality and associated risk factors in Zambia: a multi survey analysis between 2007 and 2018. BMC Pediatr. 2022;22(1):341. 35698091 10.1186/s12887-022-03362-7 PMC9190164

[14] Selebano KM, Ataguba JE. Decomposing socio-economic inequalities in antenatal care utilisation in 12 Southern African Development Community countries. SSM Popul Health. 2022;17:101004. 34988282 10.1016/j.ssmph.2021.101004 PMC8703074

[15] Bobo FT, Asante A, Woldie M, Dawson A, Hayen A. Child vaccination in sub-Saharan Africa: Increasing coverage addresses inequalities. Vaccine. 2022;40(1):141–50. 34794824 10.1016/j.vaccine.2021.11.005

[16] Carter A, Msemburi W, Sim SY, Gaythorpe KAM, Lambach P, Lindstrand A, Hutubessy R. Modeling the impact of vaccination for the immunization Agenda 2030: Deaths averted due to vaccination against 14 pathogens in 194 countries from 2021 to 2030. Vaccine. 2024;42(Suppl 1):S28–37. 37537094 10.1016/j.vaccine.2023.07.033

[17] Myemba DT, Smets L, Sunguya BF, Vandaele N, Decouttere C. Challenges and strategies for sustainable and resilient immunization systems in sub-Saharan Africa: A comprehensive scoping review. Vaccine. 2025;45:126639. 39719771 10.1016/j.vaccine.2024.126639

[18] Bovu A, Yirga A, Melesse S, Ayele D. The Impact of Socio-Economic, Demographic, and Geographic Factors on the Mortality of Children Under the Age of Five in Kenya, 2022. Iran J Public Health. 2024;53(11):2462–72. 39619917 10.18502/ijph.v53i11.16949 PMC11607168

[19] Jakubowski A, Stearns SC, Kruk ME, Angeles G, Thirumurthy H. The US President’s Malaria Initiative and under-5 child mortality in sub-Saharan Africa: A difference-in-differences analysis. PLoS Med. 2017;14(6):e1002319. 28609442 10.1371/journal.pmed.1002319 PMC5469567

[20] Phiri KS, Khairallah C, Kwambai TK, Bojang K, Dhabangi A, Opoka R, Idro R, Stepniewska K, van Hensbroek MB, John CC, et al. Post-discharge malaria chemoprevention in children admitted with severe anaemia in malaria-endemic settings in Africa: a systematic review and individual patient data meta-analysis of randomised controlled trials. Lancet Glob Health. 2024;12(1):e33–44. 38097295 10.1016/S2214-109X(23)00492-8 PMC10733130

[21] Shirima G, Masserey T, Gervas H, Chitnis N, Kiware S, Mirau S. Assessing the role of community involvement and capacity building in larviciding applications for malaria control in Africa: A scoping review. Curr Res Parasitol Vector Borne Dis. 2025;8:100307. 40893609 10.1016/j.crpvbd.2025.100307 PMC12396400

[22] Dasgupta RR, Mao W, Ogbuoji O. Addressing child health inequity through case management of under-five malaria in Nigeria: an extended cost-effectiveness analysis. Malar J. 2022;21(1):81. 35264153 10.1186/s12936-022-04113-w PMC8905868

[23] Rahman MM, Alam K, Khanam R. Socio-economic factors affecting high infant and child mortality rates in selected African countries: does globalisation play any role? Globalization Health. 2022;18(1):69. 35799303 10.1186/s12992-022-00855-z PMC9261177

[24] Abukari Z, Kuyini AB, Kuyini Mohammed A. Education and Health Care Policies in Ghana: Examining the Prospects and Challenges of Recent Provisions. SAGE Open. 2015:5 (4):1–11.

[25] Adegoke YO, Mbonigaba J, George G. Macro-economic determinants, maternal and infant SDG targets in Nigeria: Correlation and predictive modeling. Front Public Health. 2022;10:999514. 36579062 10.3389/fpubh.2022.999514 PMC9791089

[26] Amegbor PM. Early-life environmental exposures and anaemia among children under age five in Sub-Saharan Africa: An insight from the Demographic & Health Surveys. Sci Total Environ. 2022;832:154957. 35367541 10.1016/j.scitotenv.2022.154957

[27] Mbunge E, Chemhaka G, Dzinamarira T, Moyo E, Fashoto S, Muchemwa B, Buwerimwe J, Petrus EJW. Determinants of under-five mortality in Zimbabwe: Evidence from the 2015–2016 Zimbabwe demographic Health Survey data. Women and Children Nursing. 2024;2(1):1–8.

[28] Simmons RA, Anthopolos R, O’Meara WP. Effect of health systems context on infant and child mortality in sub-Saharan Africa from 1995 to 2015, a longitudinal cohort analysis. Sci Rep. 2021;11(1):16263. 34381150 10.1038/s41598-021-95886-8 PMC8357794

[29] Liyew B, Tesfa K, Altaye KD, Gelaw AD, Bicha AT, Mamo AG, Adane KC. Parametric modeling of under-5 children survival among 30 African countries: Lognormal accelerated failure time gamma shared frailty model. PLoS ONE. 2025;20(1):e0314955. 39854334 10.1371/journal.pone.0314955 PMC11759998

[30] Yahaya H, Adeyemo QE, Kumah A. Adverse perinatal outcomes and their associated determinants in Sub-Saharan Africa. J Med Surg Public Health. 2024;3:100124.

[31] Quentin W, Abosede O, Aka J, Akweongo P, Dinard K, Ezeh A, Hamed R, Kayembe PK, Mitike G, Mtei G, et al. Inequalities in child mortality in ten major African cities. BMC Med. 2014;12:95. 24906463 10.1186/1741-7015-12-95 PMC4066831

[32] Osborne A, Bai-Sesay AU, Bangura C, Rogers H, Ahinkorah BO. Socio-economic and geographical inequalities in neonatal mortality rates in Sierra Leone, 2008–2019. BMC Pediatr. 2024;24(1):761. 39578763 10.1186/s12887-024-05189-w PMC11585221

[33] Rahman MM, Rouyard T, Khan ST, Nakamura R, Islam MR, Hossain MS, Akter S, Lohan M, Ali M, Sato M. Reproductive, maternal, newborn, and child health intervention coverage in 70 low-income and middle-income countries, 2000-30: trends, projections, and inequities. Lancet Glob Health. 2023;11(10):e1531–43. 37678321 10.1016/S2214-109X(23)00358-3 PMC10509036

[34] Worku MG, Teshale AB, Tesema GA. Determinants of under-five mortality in the high mortality regions of Ethiopia: mixed-effect logistic regression analysis. Archives Public Health. 2021;79(1):55. 10.1186/s13690-021-00578-4 PMC8063408 33892785

[35] Gaffan N, Kpozehouen A, Degbey C, Ahanhanzo YG, Paraïso MN. Effects of household access to water, sanitation, and hygiene services on under-five mortality in Sub-Saharan Africa. Front Public Health. 2023;11:1136299. 37181724 10.3389/fpubh.2023.1136299 PMC10173862

[36] Bagamian KH, Anderson JDt, Muhib F, Cumming O, Laytner LA, Wierzba TF, Rheingans R. Heterogeneity in enterotoxigenic Escherichia coli and shigella infections in children under 5 years of age from 11 African countries: a subnational approach quantifying risk, mortality, morbidity, and stunting. Lancet Glob Health. 2020;8(1):e101–12. 31734154 10.1016/S2214-109X(19)30456-5 PMC7024994

[37] Hsiao C. Panel data analysis—advantages and challenges. TEST: Official J Span Soc Stat Oper Res. 2007;16:1–22.

[38] Wooldridge JrM. Econometric Analysis of Cross Section and Panel Data. Cambridge, Massachusetts, London, England: The MIT Press; 2010.

[39] Zakaria M, Tariq S, Ul Husnain MI. Socio-economic, macroeconomic, demographic, and environmental variables as determinants of child mortality in South Asia. Environ Sci Pollut Res Int. 2020;27(1):954–64. 31820247 10.1007/s11356-019-06988-w

[40] Sibanda K, Qoko A, Gonese D. Health Expenditure, Institutional Quality, and Under-Five Mortality in Sub-Saharan African Countries. Int J Environ Res Public Health. 2024;21(3):333. 10.3390/ijerph21030333 PMC10970304 38541331

[41] Novignon J, Olakojo SA, Nonvignon J. The effects of public and private health care expenditure on health status in sub-Saharan Africa: new evidence from panel data analysis. Health Econ Rev. 2012;2(1):22. 23232089 10.1186/2191-1991-2-22 PMC3533939

[42] Mhlanga M. The Impact of Human Capital Development on the Under-5 Mortality Rate in South Africa. J Econ Social Dynamics. 2025;1(3):1–12.

