# Supplementary material for: Determinants of under-five mortality in Africa: evidence from a two-decade panel analysis for public health policy
Source: Arch Public Health. 2026 Jun 17;84:193. doi: 10.1186/s13690-026-01994-0 (PMC13520361; doi:10.1186/s13690-026-01994-0)
Supplement: Supplementary file 1 [file 13690_2026_1994_MOESM10_ESM.docx]

**Appendix 10: Country-specific analysis (Descriptive illustrations)**

**Table 10.A: Descriptive country-specific multiple linear regression results identifying associations between selected determinants and under-five mortality in 45 individual African nations (2000–2021)**

**MLR Results for Country ID: 1 - Algeria**

| mortality_u5 | Coef. | | St.Err. | t-value | | p-value | [95% Conf | | Interval] | | Sig |
| --- | --- | --- | --- | --- | --- | --- | --- | --- | --- | --- | --- |
| imm_dtp | .277 | | .12 | 2.30 | | .042 | .012 | | .541 | | ** |
| malaria_inc | -1.665 | | 39.131 | -0.04 | | .967 | -87.791 | | 84.461 | |  |
| adolescent_fert | -5.133 | | 6.485 | -0.79 | | .445 | -19.406 | | 9.14 | |  |
| fert_rate | -2.766 | | 4.656 | -0.59 | | .564 | -13.014 | | 7.481 | |  |
| ln_basic_sanitation | 66.374 | | 89.921 | 0.74 | | .476 | -131.54 | | 264.288 | |  |
| adolescent_fert_sq~d | .338 | | .338 | 1.00 | | .339 | -.405 | | 1.081 | |  |
| lag_gdp_per_capita | -.011 | | .002 | -4.78 | | .001 | -.016 | | -.006 | | *** |
| lag_gdp_squared | 0 | | 0 | 4.51 | | .001 | 0 | | 0 | | *** |
| lag_health_expendi~e | -1.833 | | .579 | -3.17 | | .009 | -3.108 | | -.559 | | *** |
| Constant | -231.145 | | 390.861 | -0.59 | | .566 | -1091.424 | | 629.133 | |  |
|  | | | | | | | | | | | |
| Mean dependent var | | 28.919 | | | SD dependent var | | | 5.454 | |  |  |
| Number of obs | | 21 | | | F-test | | | 121.944 | |  |  |
| **** p<.01, ** p<.05, * p<.1* | | | | | | | | | | | |
|  | | | | | | | | | | | |

**MLR Results for Country ID: 2 - Angola**

| mortality_u5 | Coef. | | St.Err. | t-value | | p-value | [95% Conf | | Interval] | | Sig |
| --- | --- | --- | --- | --- | --- | --- | --- | --- | --- | --- | --- |
| imm_dtp | .37 | | .061 | 6.06 | | 0 | .236 | | .505 | | *** |
| malaria_inc | .073 | | .004 | 20.75 | | 0 | .066 | | .081 | | *** |
| adolescent_fert | 13.037 | | 4.452 | 2.93 | | .014 | 3.239 | | 22.835 | | ** |
| fert_rate | -21.63 | | 12.637 | -1.71 | | .115 | -49.444 | | 6.183 | |  |
| ln_basic_sanitation | -244.862 | | 24.189 | -10.12 | | 0 | -298.102 | | -191.623 | | *** |
| adolescent_fert_sq~d | -.039 | | .014 | -2.89 | | .015 | -.069 | | -.009 | | ** |
| lag_gdp_per_capita | -.003 | | .001 | -3.07 | | .011 | -.004 | | -.001 | | ** |
| lag_gdp_squared | 0 | | 0 | 2.44 | | .033 | 0 | | 0 | | ** |
| lag_health_expendi~e | -.25 | | .378 | -0.66 | | .522 | -1.081 | | .582 | |  |
| Constant | 59.112 | | 209.169 | 0.28 | | .783 | -401.266 | | 519.49 | |  |
|  | | | | | | | | | | | |
| Mean dependent var | | 121.305 | | | SD dependent var | | | 42.459 | |  |  |
| Number of obs | | 21 | | | F-test | | | 33719.200 | |  |  |
| **** p<.01, ** p<.05, * p<.1* | | | | | | | | | | | |
|  | | | | | | | | | | | |

**MLR Results for Country ID: 3 - Benin**

| mortality_u5 | | Coef. | |  | | St.Err. | t-value | | p-value | [95% Conf | | Interval] | | Sig |
| --- | --- | --- | --- | --- | --- | --- | --- | --- | --- | --- | --- | --- | --- | --- |
| imm_dtp | | -.014 | |  | | .019 | -0.73 | | .479 | -.054 | | .027 | |  |
| malaria_inc | | 0 | |  | | .01 | -0.00 | | .997 | -.022 | | .022 | |  |
| adolescent_fert | | -.48 | |  | | 1.037 | -0.46 | | .652 | -2.763 | | 1.803 | |  |
| fert_rate | | 11.914 | |  | | 9.936 | 1.20 | | .256 | -9.955 | | 33.783 | |  |
| ln_basic_sanitation | | -49.179 | |  | | 7.118 | -6.91 | | 0 | -64.846 | | -33.512 | | *** |
| adolescent_fert_sq~d | | .002 | |  | | .004 | 0.39 | | .702 | -.008 | | .011 | |  |
| lag_gdp_per_capita | | -.01 | |  | | .01 | -1.07 | | .308 | -.032 | | .011 | |  |
| lag_gdp_squared | | 0 | |  | | 0 | 0.68 | | .513 | 0 | | 0 | |  |
| lag_health_expendi~e | | -1.54 | |  | | .724 | -2.13 | | .057 | -3.133 | | .054 | | * |
| Constant | | 213.682 | |  | | 15.745 | 13.57 | | 0 | 179.028 | | 248.336 | | *** |
|  |  | | | | | | | | | | | | | |
| Mean dependent var | | |  | | 106.724 | | | SD dependent var | | | 14.941 | |  |  |
| Number of obs | | |  | | 21 | | | F-test | | | 10879.768 | |  |  |
|  | **** p<.01, ** p<.05, * p<.1* | | | | | | | | | | | | | |
|  |  | | | | | | | | | | | | | |

**MLR Results for Country ID: 4 - Botswana**

| mortality_u5 | Coef. | | St.Err. | t-value | | p-value | [95% Conf | | Interval] | | Sig |
| --- | --- | --- | --- | --- | --- | --- | --- | --- | --- | --- | --- |
| imm_dtp | .225 | | 1.079 | 0.21 | | .838 | -2.149 | | 2.6 | |  |
| malaria_inc | -1.018 | | .439 | -2.32 | | .041 | -1.984 | | -.052 | | ** |
| adolescent_fert | -6.417 | | 1.878 | -3.42 | | .006 | -10.55 | | -2.284 | | *** |
| fert_rate | -1.476 | | 9.842 | -0.15 | | .884 | -23.138 | | 20.187 | |  |
| ln_basic_sanitation | -79.419 | | 17.702 | -4.49 | | .001 | -118.382 | | -40.456 | | *** |
| adolescent_fert_sq~d | .057 | | .018 | 3.24 | | .008 | .018 | | .095 | | *** |
| lag_gdp_per_capita | -.001 | | .003 | -0.35 | | .734 | -.008 | | .006 | |  |
| lag_gdp_squared | 0 | | 0 | 0.47 | | .651 | 0 | | 0 | |  |
| lag_health_expendi~e | 2.325 | | .799 | 2.91 | | .014 | .566 | | 4.084 | | ** |
| Constant | 543.567 | | 92.583 | 5.87 | | 0 | 339.793 | | 747.341 | | *** |
|  | | | | | | | | | | | |
| Mean dependent var | | 58.119 | | | SD dependent var | | | 12.572 | |  |  |
| Number of obs | | 21 | | | F-test | | | 948.510 | |  |  |
| **** p<.01, ** p<.05, * p<.1* | | | | | | | | | | | |
|  | | | | | | | | | | | |

**MLR Results for Country ID: 5 - Burkina Faso**

| mortality_u5 | Coef. | | St.Err. | t-value | | p-value | [95% Conf | | Interval] | | Sig |
| --- | --- | --- | --- | --- | --- | --- | --- | --- | --- | --- | --- |
| imm_dtp | .454 | | .157 | 2.90 | | .015 | .109 | | .799 | | ** |
| malaria_inc | -.021 | | .008 | -2.74 | | .019 | -.038 | | -.004 | | ** |
| adolescent_fert | -3.182 | | 1.655 | -1.92 | | .081 | -6.825 | | .461 | | * |
| fert_rate | 28.847 | | 11.245 | 2.57 | | .026 | 4.097 | | 53.596 | | ** |
| ln_basic_sanitation | -91.109 | | 28.056 | -3.25 | | .008 | -152.86 | | -29.358 | | *** |
| adolescent_fert_sq~d | .011 | | .007 | 1.63 | | .131 | -.004 | | .026 | |  |
| lag_gdp_per_capita | -.035 | | .044 | -0.79 | | .444 | -.132 | | .062 | |  |
| lag_gdp_squared | 0 | | 0 | 0.88 | | .397 | 0 | | 0 | |  |
| lag_health_expendi~e | -1.059 | | .922 | -1.15 | | .275 | -3.088 | | .97 | |  |
| Constant | 427.104 | | 47.688 | 8.96 | | 0 | 322.143 | | 532.066 | | *** |
|  | | | | | | | | | | | |
| Mean dependent var | | 122.248 | | | SD dependent var | | | 29.464 | |  |  |
| Number of obs | | 21 | | | F-test | | | 1750.860 | |  |  |
| **** p<.01, ** p<.05, * p<.1* | | | | | | | | | | | |
|  | | | | | | | | | | | |

**MLR Results for Country ID: 6 - Burundi**

| mortality_u5 | Coef. | | St.Err. | t-value | | p-value | [95% Conf | | Interval] | | Sig |
| --- | --- | --- | --- | --- | --- | --- | --- | --- | --- | --- | --- |
| imm_dtp | -.221 | | .183 | -1.21 | | .252 | -.624 | | .182 | |  |
| malaria_inc | .071 | | .026 | 2.76 | | .018 | .014 | | .127 | | ** |
| adolescent_fert | 2.563 | | 3.97 | 0.65 | | .532 | -6.176 | | 11.302 | |  |
| fert_rate | 32.633 | | 6.929 | 4.71 | | .001 | 17.382 | | 47.884 | | *** |
| ln_basic_sanitation | -1139.742 | | 409.494 | -2.78 | | .018 | -2041.033 | | -238.451 | | ** |
| adolescent_fert_sq~d | -.014 | | .027 | -0.50 | | .624 | -.074 | | .046 | |  |
| lag_gdp_per_capita | -.337 | | .144 | -2.34 | | .039 | -.653 | | -.02 | | ** |
| lag_gdp_squared | .001 | | 0 | 2.50 | | .029 | 0 | | .001 | | ** |
| lag_health_expendi~e | .439 | | .371 | 1.18 | | .262 | -.378 | | 1.256 | |  |
| Constant | 4176.08 | | 1609.015 | 2.60 | | .025 | 634.662 | | 7717.498 | | ** |
|  | | | | | | | | | | | |
| Mean dependent var | | 91.871 | | | SD dependent var | | | 31.561 | |  |  |
| Number of obs | | 21 | | | F-test | | | 3525.792 | |  |  |
| **** p<.01, ** p<.05, * p<.1* | | | | | | | | | | | |
|  | | | | | | | | | | | |

**MLR Results for Country ID: 7 - Cabo Verde**

| mortality_u5 | Coef. | | St.Err. | t-value | | p-value | [95% Conf | | Interval] | | Sig |
| --- | --- | --- | --- | --- | --- | --- | --- | --- | --- | --- | --- |
| imm_dtp | -.119 | | .066 | -1.82 | | .097 | -.264 | | .025 | | * |
| malaria_inc | -.238 | | .189 | -1.26 | | .234 | -.653 | | .178 | |  |
| adolescent_fert | .035 | | .186 | 0.19 | | .856 | -.374 | | .444 | |  |
| fert_rate | -1.468 | | 3.782 | -0.39 | | .705 | -9.792 | | 6.856 | |  |
| ln_basic_sanitation | -51.795 | | 10.672 | -4.85 | | .001 | -75.284 | | -28.306 | | *** |
| adolescent_fert_sq~d | -.001 | | .002 | -0.56 | | .586 | -.005 | | .003 | |  |
| lag_gdp_per_capita | .013 | | .003 | 4.38 | | .001 | .007 | | .02 | | *** |
| lag_gdp_squared | 0 | | 0 | -4.19 | | .002 | 0 | | 0 | | *** |
| lag_health_expendi~e | -.616 | | .312 | -1.97 | | .074 | -1.303 | | .071 | | * |
| Constant | 234.684 | | 54.357 | 4.32 | | .001 | 115.045 | | 354.324 | | *** |
|  | | | | | | | | | | | |
| Mean dependent var | | 23.238 | | | SD dependent var | | | 6.294 | |  |  |
| Number of obs | | 21 | | | F-test | | | 374.958 | |  |  |
| **** p<.01, ** p<.05, * p<.1* | | | | | | | | | | | |
|  | | | | | | | | | | | |

**MLR Results for Country ID: 8 - Cameroon**

| mortality_u5 | Coef. | | St.Err. | t-value | | p-value | [95% Conf | | Interval] | | Sig |
| --- | --- | --- | --- | --- | --- | --- | --- | --- | --- | --- | --- |
| imm_dtp | .014 | | .09 | 0.15 | | .881 | -.185 | | .212 | |  |
| malaria_inc | -.039 | | .017 | -2.23 | | .048 | -.077 | | 0 | | ** |
| adolescent_fert | -11.242 | | 2.001 | -5.62 | | 0 | -15.646 | | -6.838 | | *** |
| fert_rate | 20.791 | | 8.431 | 2.47 | | .031 | 2.235 | | 39.348 | | ** |
| ln_basic_sanitation | -653.681 | | 30.85 | -21.19 | | 0 | -721.582 | | -585.78 | | *** |
| adolescent_fert_sq~d | .043 | | .007 | 5.82 | | 0 | .027 | | .06 | | *** |
| lag_gdp_per_capita | .02 | | .019 | 1.09 | | .301 | -.021 | | .062 | |  |
| lag_gdp_squared | 0 | | 0 | -1.01 | | .335 | 0 | | 0 | |  |
| lag_health_expendi~e | -.57 | | .849 | -0.67 | | .515 | -2.439 | | 1.298 | |  |
| Constant | 3145.429 | | 204.331 | 15.39 | | 0 | 2695.7 | | 3595.158 | | *** |
|  | | | | | | | | | | | |
| Mean dependent var | | 105.719 | | | SD dependent var | | | 22.595 | |  |  |
| Number of obs | | 21 | | | F-test | | | 5284.618 | |  |  |
| **** p<.01, ** p<.05, * p<.1* | | | | | | | | | | | |
|  | | | | | | | | | | | |

**MLR Results for Country ID: 9 - Central African Republic**

| mortality_u5 | Coef. | | St.Err. | t-value | | p-value | [95% Conf | | Interval] | | Sig |
| --- | --- | --- | --- | --- | --- | --- | --- | --- | --- | --- | --- |
| imm_dtp | -5.266 | | 5.282 | -1.00 | | .34 | -16.892 | | 6.36 | |  |
| malaria_inc | -.101 | | 3.599 | -0.03 | | .978 | -8.022 | | 7.82 | |  |
| adolescent_fert | -96.705 | | 250.706 | -0.39 | | .707 | -648.505 | | 455.096 | |  |
| fert_rate | -4.258 | | 450.839 | -0.01 | | .993 | -996.548 | | 988.033 | |  |
| ln_basic_sanitation | 663.874 | | 1536.746 | 0.43 | | .674 | -2718.48 | | 4046.228 | |  |
| adolescent_fert_sq~d | .296 | | .786 | 0.38 | | .714 | -1.434 | | 2.026 | |  |
| lag_gdp_per_capita | 6.999 | | 9.483 | 0.74 | | .476 | -13.871 | | 27.87 | |  |
| lag_gdp_squared | -.008 | | .01 | -0.77 | | .455 | -.031 | | .015 | |  |
| lag_health_expendi~e | 19.637 | | 13.906 | 1.41 | | .186 | -10.97 | | 50.244 | |  |
| Constant | 4901.163 | | 20659.488 | 0.24 | | .817 | -40570.062 | | 50372.389 | |  |
|  | | | | | | | | | | | |
| Mean dependent var | | 163.767 | | | SD dependent var | | | 77.525 | |  |  |
| Number of obs | | 21 | | | F-test | | | 2.305 | |  |  |
| **** p<.01, ** p<.05, * p<.1* | | | | | | | | | | | |
|  | | | | | | | | | | | |

**MLR Results for Country ID: 10 - Chad**

| mortality_u5 | Coef. | | St.Err. | t-value | | p-value | [95% Conf | | Interval] | | Sig |
| --- | --- | --- | --- | --- | --- | --- | --- | --- | --- | --- | --- |
| imm_dtp | .067 | | .024 | 2.82 | | .017 | .015 | | .119 | | ** |
| malaria_inc | -.013 | | .036 | -0.35 | | .732 | -.092 | | .067 | |  |
| adolescent_fert | -4.087 | | .908 | -4.50 | | .001 | -6.085 | | -2.089 | | *** |
| fert_rate | 62.31 | | 31.798 | 1.96 | | .076 | -7.678 | | 132.297 | | * |
| ln_basic_sanitation | -140.132 | | 119.729 | -1.17 | | .267 | -403.654 | | 123.389 | |  |
| adolescent_fert_sq~d | .011 | | .003 | 3.93 | | .002 | .005 | | .017 | | *** |
| lag_gdp_per_capita | -.016 | | .005 | -3.41 | | .006 | -.027 | | -.006 | | *** |
| lag_gdp_squared | 0 | | 0 | 5.02 | | 0 | 0 | | 0 | | *** |
| lag_health_expendi~e | .645 | | .802 | 0.80 | | .439 | -1.121 | | 2.41 | |  |
| Constant | 436.539 | | 553.856 | 0.79 | | .447 | -782.49 | | 1655.568 | |  |
|  | | | | | | | | | | | |
| Mean dependent var | | 144.076 | | | SD dependent var | | | 22.789 | |  |  |
| Number of obs | | 21 | | | F-test | | | 3760.785 | |  |  |
| **** p<.01, ** p<.05, * p<.1* | | | | | | | | | | | |
|  | | | | | | | | | | | |

**MLR Results for Country ID: 11 - Comoros**

| mortality_u5 | Coef. | | St.Err. | t-value | | p-value | [95% Conf | | Interval] | | Sig |
| --- | --- | --- | --- | --- | --- | --- | --- | --- | --- | --- | --- |
| imm_dtp | -.017 | | .038 | -0.46 | | .655 | -.101 | | .066 | |  |
| malaria_inc | -.007 | | .008 | -0.92 | | .377 | -.024 | | .01 | |  |
| adolescent_fert | -.568 | | .777 | -0.73 | | .48 | -2.279 | | 1.142 | |  |
| fert_rate | 8.472 | | 4.572 | 1.85 | | .091 | -1.591 | | 18.534 | | * |
| ln_basic_sanitation | -27.195 | | 41.62 | -0.65 | | .527 | -118.8 | | 64.41 | |  |
| adolescent_fert_sq~d | .006 | | .005 | 1.30 | | .219 | -.004 | | .017 | |  |
| lag_gdp_per_capita | -.027 | | .013 | -2.02 | | .068 | -.057 | | .002 | | * |
| lag_gdp_squared | 0 | | 0 | 1.81 | | .098 | 0 | | 0 | | * |
| lag_health_expendi~e | -.472 | | .392 | -1.20 | | .254 | -1.336 | | .392 | |  |
| Constant | 142.117 | | 141.519 | 1.00 | | .337 | -169.364 | | 453.598 | |  |
|  | | | | | | | | | | | |
| Mean dependent var | | 55.914 | | | SD dependent var | | | 10.350 | |  |  |
| Number of obs | | 21 | | | F-test | | | 1678.784 | |  |  |
| **** p<.01, ** p<.05, * p<.1* | | | | | | | | | | | |
|  | | | | | | | | | | | |

**MLR Results for Country ID: 12 - Congo, Dem. Rep.**

| mortality_u5 | Coef. | | St.Err. | t-value | | p-value | [95% Conf | | Interval] | | Sig |
| --- | --- | --- | --- | --- | --- | --- | --- | --- | --- | --- | --- |
| imm_dtp | -.026 | | .035 | -0.74 | | .474 | -.102 | | .051 | |  |
| malaria_inc | .023 | | .015 | 1.52 | | .157 | -.01 | | .055 | |  |
| adolescent_fert | 4.225 | | 4.654 | 0.91 | | .383 | -6.018 | | 14.468 | |  |
| fert_rate | -1.04 | | 6.928 | -0.15 | | .883 | -16.289 | | 14.209 | |  |
| ln_basic_sanitation | 195.979 | | 14.855 | 13.19 | | 0 | 163.284 | | 228.675 | | *** |
| adolescent_fert_sq~d | -.019 | | .02 | -0.97 | | .354 | -.062 | | .024 | |  |
| lag_gdp_per_capita | -.024 | | .018 | -1.34 | | .208 | -.064 | | .015 | |  |
| lag_gdp_squared | 0 | | 0 | 1.00 | | .338 | 0 | | 0 | |  |
| lag_health_expendi~e | -1.78 | | .722 | -2.46 | | .031 | -3.369 | | -.191 | | ** |
| Constant | -688.898 | | 259.462 | -2.66 | | .022 | -1259.97 | | -117.825 | | ** |
|  | | | | | | | | | | | |
| Mean dependent var | | 112.405 | | | SD dependent var | | | 24.068 | |  |  |
| Number of obs | | 21 | | | F-test | | | 12859.227 | |  |  |
| **** p<.01, ** p<.05, * p<.1* | | | | | | | | | | | |
|  | | | | | | | | | | | |

**MLR Results for Country ID: 13 - Congo Rep.**

| mortality_u5 | Coef. | | St.Err. | t-value | | p-value | [95% Conf | | Interval] | | Sig |
| --- | --- | --- | --- | --- | --- | --- | --- | --- | --- | --- | --- |
| imm_dtp | -.196 | | .05 | -3.94 | | .002 | -.306 | | -.087 | | *** |
| malaria_inc | .01 | | .051 | 0.19 | | .854 | -.103 | | .123 | |  |
| adolescent_fert | -6.294 | | 6.581 | -0.96 | | .359 | -20.779 | | 8.191 | |  |
| fert_rate | 12.627 | | 11.657 | 1.08 | | .302 | -13.031 | | 38.285 | |  |
| ln_basic_sanitation | -94.23 | | 16.977 | -5.55 | | 0 | -131.597 | | -56.864 | | *** |
| adolescent_fert_sq~d | .022 | | .025 | 0.91 | | .383 | -.032 | | .077 | |  |
| lag_gdp_per_capita | -.009 | | .005 | -1.85 | | .092 | -.02 | | .002 | | * |
| lag_gdp_squared | 0 | | 0 | 1.81 | | .098 | 0 | | 0 | | * |
| lag_health_expendi~e | -3.355 | | 2.02 | -1.66 | | .125 | -7.8 | | 1.09 | |  |
| Constant | 736.121 | | 453.774 | 1.62 | | .133 | -262.627 | | 1734.87 | |  |
|  | | | | | | | | | | | |
| Mean dependent var | | 67.581 | | | SD dependent var | | | 20.828 | |  |  |
| Number of obs | | 21 | | | F-test | | | 1360.317 | |  |  |
| **** p<.01, ** p<.05, * p<.1* | | | | | | | | | | | |
|  | | | | | | | | | | | |

**MLR Results for Country ID: 14 - Cotr d’Ivore**

| mortality_u5 | Coef. | | St.Err. | t-value | | p-value | [95% Conf | | Interval] | | Sig |
| --- | --- | --- | --- | --- | --- | --- | --- | --- | --- | --- | --- |
| imm_dtp | .016 | | .009 | 1.89 | | .085 | -.003 | | .035 | | * |
| malaria_inc | .003 | | .001 | 2.02 | | .068 | 0 | | .005 | | * |
| adolescent_fert | -.807 | | .272 | -2.97 | | .013 | -1.405 | | -.209 | | ** |
| fert_rate | 5.215 | | 2.121 | 2.46 | | .032 | .546 | | 9.883 | | ** |
| ln_basic_sanitation | -115.69 | | 4.543 | -25.47 | | 0 | -125.689 | | -105.691 | | *** |
| adolescent_fert_sq~d | .003 | | .001 | 2.55 | | .027 | 0 | | .005 | | ** |
| lag_gdp_per_capita | -.014 | | .003 | -3.94 | | .002 | -.021 | | -.006 | | *** |
| lag_gdp_squared | 0 | | 0 | 3.34 | | .007 | 0 | | 0 | | *** |
| lag_health_expendi~e | -.602 | | .244 | -2.46 | | .032 | -1.14 | | -.064 | | ** |
| Constant | 528.253 | | 15.799 | 33.44 | | 0 | 493.48 | | 563.026 | | *** |
|  | | | | | | | | | | | |
| Mean dependent var | | 101.138 | | | SD dependent var | | | 20.815 | |  |  |
| Number of obs | | 21 | | | F-test | | | 128787.990 | |  |  |
| **** p<.01, ** p<.05, * p<.1* | | | | | | | | | | | |
|  | | | | | | | | | | | |

**MLR Results for Country ID: 15 - Djibouti**

| mortality_u5 | Coef. | | St.Err. | t-value | | p-value | [95% Conf | | Interval] | | Sig |
| --- | --- | --- | --- | --- | --- | --- | --- | --- | --- | --- | --- |
| imm_dtp | .022 | | .016 | 1.40 | | .19 | -.013 | | .057 | |  |
| malaria_inc | .008 | | .009 | 0.94 | | .367 | -.011 | | .028 | |  |
| adolescent_fert | -4.691 | | 1.256 | -3.73 | | .003 | -7.456 | | -1.926 | | *** |
| fert_rate | 26.88 | | 4.771 | 5.63 | | 0 | 16.378 | | 37.381 | | *** |
| ln_basic_sanitation | -8.153 | | 19.835 | -0.41 | | .689 | -51.809 | | 35.504 | |  |
| adolescent_fert_sq~d | .071 | | .024 | 2.95 | | .013 | .018 | | .124 | | ** |
| lag_gdp_per_capita | .011 | | .002 | 5.07 | | 0 | .006 | | .015 | | *** |
| lag_gdp_squared | 0 | | 0 | -6.45 | | 0 | 0 | | 0 | | *** |
| lag_health_expendi~e | .3 | | .252 | 1.19 | | .258 | -.254 | | .855 | |  |
| Constant | 76.306 | | 103.318 | 0.74 | | .476 | -151.095 | | 303.707 | |  |
|  | | | | | | | | | | | |
| Mean dependent var | | 74.381 | | | SD dependent var | | | 13.560 | |  |  |
| Number of obs | | 21 | | | F-test | | | 26900.256 | |  |  |
| **** p<.01, ** p<.05, * p<.1* | | | | | | | | | | | |
|  | | | | | | | | | | | |

**MLR Results for Country ID: 16 - Egypt, Arab Rep.**

| mortality_u5 | Coef. | | St.Err. | t-value | | p-value | [95% Conf | | Interval] | | Sig |
| --- | --- | --- | --- | --- | --- | --- | --- | --- | --- | --- | --- |
| imm_dtp | .092 | | .155 | 0.60 | | .563 | -.246 | | .43 | |  |
| o | 0 | | . | . | | . | . | | . | |  |
| adolescent_fert | -.601 | | 1.279 | -0.47 | | .647 | -3.387 | | 2.184 | |  |
| fert_rate | 3.479 | | 3.027 | 1.15 | | .273 | -3.116 | | 10.074 | |  |
| ln_basic_sanitation | -433.092 | | 23.846 | -18.16 | | 0 | -485.048 | | -381.136 | | *** |
| adolescent_fert_sq~d | .002 | | .013 | 0.15 | | .883 | -.027 | | .03 | |  |
| lag_gdp_per_capita | .004 | | .001 | 2.67 | | .02 | .001 | | .006 | | ** |
| lag_gdp_squared | 0 | | 0 | -2.10 | | .058 | 0 | | 0 | | * |
| lag_health_expendi~e | .763 | | .804 | 0.95 | | .362 | -.989 | | 2.515 | |  |
| Constant | 1997.024 | | 116.443 | 17.15 | | 0 | 1743.316 | | 2250.733 | | *** |
|  | | | | | | | | | | | |
| Mean dependent var | | 28.748 | | | SD dependent var | | | 7.655 | |  |  |
| Number of obs | | 21 | | | F-test | | | 698.273 | |  |  |
| **** p<.01, ** p<.05, * p<.1* | | | | | | | | | | | |
|  | | | | | | | | | | | |

**MLR Results for Country ID: 17 - Equatorial Guinea**

| mortality_u5 | Coef. | | St.Err. | t-value | | p-value | [95% Conf | | Interval] | | Sig |
| --- | --- | --- | --- | --- | --- | --- | --- | --- | --- | --- | --- |
| imm_dtp | -.015 | | .015 | -1.01 | | .334 | -.047 | | .017 | |  |
| malaria_inc | .015 | | .003 | 5.21 | | 0 | .009 | | .022 | | *** |
| adolescent_fert | -1.584 | | .671 | -2.36 | | .038 | -3.06 | | -.107 | | ** |
| fert_rate | 51.215 | | 2.94 | 17.42 | | 0 | 44.744 | | 57.686 | | *** |
| ln_basic_sanitation | -76.444 | | 14.67 | -5.21 | | 0 | -108.732 | | -44.156 | | *** |
| adolescent_fert_sq~d | .003 | | .002 | 1.59 | | .141 | -.001 | | .007 | |  |
| lag_gdp_per_capita | 0 | | 0 | -3.18 | | .009 | -.001 | | 0 | | *** |
| lag_gdp_squared | 0 | | 0 | 3.40 | | .006 | 0 | | 0 | | *** |
| lag_health_expendi~e | .013 | | .093 | 0.14 | | .892 | -.193 | | .219 | |  |
| Constant | 354.838 | | 27.834 | 12.75 | | 0 | 293.576 | | 416.099 | | *** |
|  | | | | | | | | | | | |
| Mean dependent var | | 109.919 | | | SD dependent var | | | 23.719 | |  |  |
| Number of obs | | 21 | | | F-test | | | 43735.139 | |  |  |
| **** p<.01, ** p<.05, * p<.1* | | | | | | | | | | | |
|  | | | | | | | | | | | |

**MLR Results for Country ID: 18 - Eswatini**

| mortality_u5 | Coef. | | St.Err. | t-value | | p-value | [95% Conf | | Interval] | | Sig |
| --- | --- | --- | --- | --- | --- | --- | --- | --- | --- | --- | --- |
| imm_dtp | -.407 | | .19 | -2.14 | | .055 | -.825 | | .011 | | * |
| malaria_inc | -.976 | | .561 | -1.74 | | .11 | -2.211 | | .259 | |  |
| adolescent_fert | 11.981 | | 2.091 | 5.73 | | 0 | 7.378 | | 16.584 | | *** |
| fert_rate | -5.926 | | 18.897 | -0.31 | | .76 | -47.518 | | 35.665 | |  |
| ln_basic_sanitation | 174.189 | | 54.216 | 3.21 | | .008 | 54.86 | | 293.518 | | *** |
| adolescent_fert_sq~d | -.048 | | .011 | -4.21 | | .001 | -.073 | | -.023 | | *** |
| lag_gdp_per_capita | .007 | | .003 | 2.66 | | .022 | .001 | | .013 | | ** |
| lag_gdp_squared | 0 | | 0 | -3.47 | | .005 | 0 | | 0 | | *** |
| lag_health_expendi~e | 2.098 | | .955 | 2.20 | | .05 | -.003 | | 4.199 | | * |
| Constant | -1253.776 | | 267.186 | -4.69 | | .001 | -1841.85 | | -665.703 | | *** |
|  | | | | | | | | | | | |
| Mean dependent var | | 79.786 | | | SD dependent var | | | 26.486 | |  |  |
| Number of obs | | 21 | | | F-test | | | 9027.768 | |  |  |
| **** p<.01, ** p<.05, * p<.1* | | | | | | | | | | | |
|  | | | | | | | | | | | |

**MLR Results for Country ID: 19 - Ethiopia**

| mortality_u5 | Coef. | | St.Err. | t-value | | p-value | [95% Conf | | Interval] | | Sig |
| --- | --- | --- | --- | --- | --- | --- | --- | --- | --- | --- | --- |
| imm_dtp | .021 | | .026 | 0.81 | | .436 | -.037 | | .08 | |  |
| malaria_inc | .009 | | .002 | 3.99 | | .002 | .004 | | .014 | | *** |
| adolescent_fert | -.2 | | .123 | -1.63 | | .132 | -.47 | | .07 | |  |
| fert_rate | 3.53 | | 1.637 | 2.16 | | .054 | -.073 | | 7.132 | | * |
| ln_basic_sanitation | -70.964 | | 3.142 | -22.59 | | 0 | -77.878 | | -64.049 | | *** |
| adolescent_fert_sq~d | .001 | | 0 | 1.70 | | .118 | 0 | | .002 | |  |
| lag_gdp_per_capita | -.013 | | .004 | -3.33 | | .007 | -.022 | | -.005 | | *** |
| lag_gdp_squared | 0 | | 0 | 4.41 | | .001 | 0 | | 0 | | *** |
| lag_health_expendi~e | .243 | | .235 | 1.03 | | .323 | -.274 | | .76 | |  |
| Constant | 202.309 | | 10.113 | 20.00 | | 0 | 180.05 | | 224.568 | | *** |
|  | | | | | | | | | | | |
| Mean dependent var | | 83.933 | | | SD dependent var | | | 26.310 | |  |  |
| Number of obs | | 21 | | | F-test | | | 62449.028 | |  |  |
| **** p<.01, ** p<.05, * p<.1* | | | | | | | | | | | |
|  | | | | | | | | | | | |

**MLR Results for Country ID: 20 - Gabon**

| mortality_u5 | Coef. | | St.Err. | t-value | | p-value | [95% Conf | | Interval] | | Sig |
| --- | --- | --- | --- | --- | --- | --- | --- | --- | --- | --- | --- |
| imm_dtp | -.026 | | .017 | -1.56 | | .148 | -.063 | | .011 | |  |
| malaria_inc | .009 | | .011 | 0.83 | | .422 | -.015 | | .033 | |  |
| adolescent_fert | 7.46 | | 1.723 | 4.33 | | .001 | 3.668 | | 11.251 | | *** |
| fert_rate | 20.594 | | 5.919 | 3.48 | | .005 | 7.566 | | 33.621 | | *** |
| ln_basic_sanitation | -122.348 | | 21.87 | -5.59 | | 0 | -170.483 | | -74.213 | | *** |
| adolescent_fert_sq~d | -.036 | | .009 | -4.25 | | .001 | -.055 | | -.018 | | *** |
| lag_gdp_per_capita | .001 | | .002 | 0.50 | | .628 | -.003 | | .005 | |  |
| lag_gdp_squared | 0 | | 0 | -0.31 | | .765 | 0 | | 0 | |  |
| lag_health_expendi~e | 1.33 | | .655 | 2.03 | | .067 | -.112 | | 2.772 | | * |
| Constant | 45.826 | | 44.652 | 1.03 | | .327 | -52.453 | | 144.105 | |  |
|  | | | | | | | | | | | |
| Mean dependent var | | 52.348 | | | SD dependent var | | | 11.737 | |  |  |
| Number of obs | | 21 | | | F-test | | | 2281.462 | |  |  |
| **** p<.01, ** p<.05, * p<.1* | | | | | | | | | | | |
|  | | | | | | | | | | | |

**MLR Results for Country ID: 21 - Gambia, The**

| mortality_u5 | Coef. | | St.Err. | t-value | | p-value | [95% Conf | | Interval] | | Sig |
| --- | --- | --- | --- | --- | --- | --- | --- | --- | --- | --- | --- |
| imm_dtp | .115 | | .204 | 0.56 | | .584 | -.334 | | .565 | |  |
| malaria_inc | -.038 | | .021 | -1.85 | | .092 | -.084 | | .007 | | * |
| adolescent_fert | -6.165 | | 2.661 | -2.32 | | .041 | -12.022 | | -.309 | | ** |
| fert_rate | 19.686 | | 20.565 | 0.96 | | .359 | -25.577 | | 64.949 | |  |
| ln_basic_sanitation | 193.069 | | 108.065 | 1.79 | | .102 | -44.781 | | 430.918 | |  |
| adolescent_fert_sq~d | .043 | | .012 | 3.50 | | .005 | .016 | | .071 | | *** |
| lag_gdp_per_capita | -.018 | | .049 | -0.37 | | .72 | -.125 | | .089 | |  |
| lag_gdp_squared | 0 | | 0 | -0.03 | | .974 | 0 | | 0 | |  |
| lag_health_expendi~e | -6.635 | | 2.948 | -2.25 | | .046 | -13.123 | | -.146 | | ** |
| Constant | -535.336 | | 426.637 | -1.25 | | .236 | -1474.358 | | 403.686 | |  |
|  | | | | | | | | | | | |
| Mean dependent var | | 72.957 | | | SD dependent var | | | 19.070 | |  |  |
| Number of obs | | 21 | | | F-test | | | 760.999 | |  |  |
| **** p<.01, ** p<.05, * p<.1* | | | | | | | | | | | |
|  | | | | | | | | | | | |

**MLR Results for Country ID: 22 - Ghana**

| mortality_u5 | Coef. | | St.Err. | t-value | | p-value | [95% Conf | | Interval] | | Sig |
| --- | --- | --- | --- | --- | --- | --- | --- | --- | --- | --- | --- |
| imm_dtp | .034 | | .051 | 0.67 | | .517 | -.078 | | .147 | |  |
| malaria_inc | -.017 | | .006 | -2.67 | | .022 | -.03 | | -.003 | | ** |
| adolescent_fert | 2.362 | | .83 | 2.85 | | .016 | .535 | | 4.19 | | ** |
| fert_rate | 5.157 | | 5.641 | 0.91 | | .38 | -7.259 | | 17.574 | |  |
| ln_basic_sanitation | -47.888 | | 3.269 | -14.65 | | 0 | -55.083 | | -40.694 | | *** |
| adolescent_fert_sq~d | -.02 | | .006 | -3.55 | | .005 | -.032 | | -.008 | | *** |
| lag_gdp_per_capita | .007 | | .004 | 1.86 | | .09 | -.001 | | .015 | | * |
| lag_gdp_squared | 0 | | 0 | -2.31 | | .041 | 0 | | 0 | | ** |
| lag_health_expendi~e | .562 | | .361 | 1.56 | | .148 | -.232 | | 1.356 | |  |
| Constant | 102.009 | | 10.18 | 10.02 | | 0 | 79.604 | | 124.415 | | *** |
|  | | | | | | | | | | | |
| Mean dependent var | | 65.133 | | | SD dependent var | | | 17.579 | |  |  |
| Number of obs | | 21 | | | F-test | | | 7114.462 | |  |  |
| **** p<.01, ** p<.05, * p<.1* | | | | | | | | | | | |
|  | | | | | | | | | | | |

**MLR Results for Country ID: 23 - Guinea**

| mortality_u5 | Coef. | | St.Err. | t-value | | p-value | [95% Conf | | Interval] | | Sig |
| --- | --- | --- | --- | --- | --- | --- | --- | --- | --- | --- | --- |
| imm_dtp | -.192 | | .041 | -4.70 | | .001 | -.281 | | -.102 | | *** |
| malaria_inc | .031 | | .022 | 1.40 | | .19 | -.018 | | .081 | |  |
| adolescent_fert | -3.817 | | 3.963 | -0.96 | | .356 | -12.539 | | 4.906 | |  |
| fert_rate | -8.946 | | 6.985 | -1.28 | | .227 | -24.32 | | 6.428 | |  |
| ln_basic_sanitation | -61.107 | | 3.689 | -16.56 | | 0 | -69.227 | | -52.988 | | *** |
| adolescent_fert_sq~d | .013 | | .013 | 1.00 | | .34 | -.016 | | .043 | |  |
| lag_gdp_per_capita | .01 | | .009 | 1.06 | | .31 | -.011 | | .031 | |  |
| lag_gdp_squared | 0 | | 0 | -1.48 | | .167 | 0 | | 0 | |  |
| lag_health_expendi~e | -.103 | | .629 | -0.16 | | .873 | -1.488 | | 1.282 | |  |
| Constant | 614.454 | | 245.551 | 2.50 | | .029 | 74.001 | | 1154.907 | | ** |
|  | | | | | | | | | | | |
| Mean dependent var | | 123.510 | | | SD dependent var | | | 16.207 | |  |  |
| Number of obs | | 21 | | | F-test | | | 893.728 | |  |  |
| **** p<.01, ** p<.05, * p<.1* | | | | | | | | | | | |
|  | | | | | | | | | | | |

**MLR Results for Country ID: 24 - Guinea - Bissay**

| mortality_u5 | Coef. | | St.Err. | t-value | | p-value | [95% Conf | | Interval] | | Sig |
| --- | --- | --- | --- | --- | --- | --- | --- | --- | --- | --- | --- |
| imm_dtp | -.164 | | .05 | -3.31 | | .007 | -.273 | | -.055 | | *** |
| malaria_inc | -.007 | | .008 | -0.83 | | .423 | -.025 | | .011 | |  |
| adolescent_fert | -6.976 | | 1.971 | -3.54 | | .005 | -11.315 | | -2.638 | | *** |
| fert_rate | 46.044 | | 13.713 | 3.36 | | .006 | 15.863 | | 76.225 | | *** |
| ln_basic_sanitation | -29.939 | | 5.247 | -5.71 | | 0 | -41.487 | | -18.391 | | *** |
| adolescent_fert_sq~d | .029 | | .008 | 3.60 | | .004 | .011 | | .047 | | *** |
| lag_gdp_per_capita | .073 | | .047 | 1.57 | | .146 | -.03 | | .175 | |  |
| lag_gdp_squared | 0 | | 0 | -1.39 | | .191 | 0 | | 0 | |  |
| lag_health_expendi~e | -.104 | | .244 | -0.43 | | .679 | -.64 | | .433 | |  |
| Constant | 358.169 | | 56.645 | 6.32 | | 0 | 233.493 | | 482.845 | | *** |
|  | | | | | | | | | | | |
| Mean dependent var | | 114.271 | | | SD dependent var | | | 30.444 | |  |  |
| Number of obs | | 21 | | | F-test | | | 14488.287 | |  |  |
| **** p<.01, ** p<.05, * p<.1* | | | | | | | | | | | |
|  | | | | | | | | | | | |

**MLR Results for Country ID: 25 - Kenya**

| mortality_u5 | Coef. | | St.Err. | t-value | | p-value | [95% Conf | | Interval] | | Sig |
| --- | --- | --- | --- | --- | --- | --- | --- | --- | --- | --- | --- |
| imm_dtp | .106 | | .044 | 2.39 | | .036 | .008 | | .203 | | ** |
| malaria_inc | .026 | | .021 | 1.26 | | .233 | -.019 | | .071 | |  |
| adolescent_fert | -1.708 | | .419 | -4.08 | | .002 | -2.63 | | -.786 | | *** |
| fert_rate | -25.86 | | 6.358 | -4.07 | | .002 | -39.852 | | -11.867 | | *** |
| ln_basic_sanitation | -398.717 | | 76.588 | -5.21 | | 0 | -567.287 | | -230.147 | | *** |
| adolescent_fert_sq~d | .009 | | .003 | 3.57 | | .004 | .003 | | .014 | | *** |
| lag_gdp_per_capita | -.002 | | .004 | -0.42 | | .686 | -.011 | | .008 | |  |
| lag_gdp_squared | 0 | | 0 | 0.11 | | .911 | 0 | | 0 | |  |
| lag_health_expendi~e | .21 | | .577 | 0.36 | | .723 | -1.06 | | 1.479 | |  |
| Constant | 1616.056 | | 291.909 | 5.54 | | 0 | 973.569 | | 2258.544 | | *** |
|  | | | | | | | | | | | |
| Mean dependent var | | 58.024 | | | SD dependent var | | | 15.345 | |  |  |
| Number of obs | | 21 | | | F-test | | | 3870.521 | |  |  |
| **** p<.01, ** p<.05, * p<.1* | | | | | | | | | | | |
|  | | | | | | | | | | | |

**MLR Results for Country ID: 26 - Liberia**

| mortality_u5 | Coef. | | St.Err. | t-value | | p-value | [95% Conf | | Interval] | | Sig |
| --- | --- | --- | --- | --- | --- | --- | --- | --- | --- | --- | --- |
| imm_dtp | -.327 | | .078 | -4.20 | | .001 | -.499 | | -.156 | | *** |
| malaria_inc | .009 | | .016 | 0.54 | | .603 | -.027 | | .045 | |  |
| adolescent_fert | -26.499 | | 8.727 | -3.04 | | .011 | -45.706 | | -7.291 | | ** |
| fert_rate | 55.992 | | 27.696 | 2.02 | | .068 | -4.967 | | 116.952 | | * |
| ln_basic_sanitation | -142.979 | | 66.94 | -2.14 | | .056 | -290.313 | | 4.355 | | * |
| adolescent_fert_sq~d | .085 | | .031 | 2.77 | | .018 | .018 | | .153 | | ** |
| lag_gdp_per_capita | .218 | | .052 | 4.21 | | .001 | .104 | | .332 | | *** |
| lag_gdp_squared | 0 | | 0 | -3.01 | | .012 | 0 | | 0 | | ** |
| lag_health_expendi~e | .555 | | .325 | 1.71 | | .115 | -.16 | | 1.27 | |  |
| Constant | 2208.08 | | 800.176 | 2.76 | | .019 | 446.904 | | 3969.257 | | ** |
|  | | | | | | | | | | | |
| Mean dependent var | | 110.586 | | | SD dependent var | | | 29.540 | |  |  |
| Number of obs | | 21 | | | F-test | | | 490.693 | |  |  |
| **** p<.01, ** p<.05, * p<.1* | | | | | | | | | | | |
|  | | | | | | | | | | | |

**MLR Results for Country ID: 27 - Madagascar**

| mortality_u5 | Coef. | | St.Err. | t-value | | p-value | [95% Conf | | Interval] | | Sig |
| --- | --- | --- | --- | --- | --- | --- | --- | --- | --- | --- | --- |
| imm_dtp | .201 | | .067 | 2.98 | | .012 | .053 | | .349 | | ** |
| malaria_inc | .055 | | .016 | 3.50 | | .005 | .02 | | .09 | | *** |
| adolescent_fert | -3.973 | | 8.793 | -0.45 | | .66 | -23.325 | | 15.379 | |  |
| fert_rate | -36.632 | | 8.346 | -4.39 | | .001 | -55.001 | | -18.263 | | *** |
| ln_basic_sanitation | -52.34 | | 7.733 | -6.77 | | 0 | -69.36 | | -35.321 | | *** |
| adolescent_fert_sq~d | .019 | | .03 | 0.64 | | .538 | -.048 | | .086 | |  |
| lag_gdp_per_capita | .031 | | .052 | 0.60 | | .558 | -.083 | | .146 | |  |
| lag_gdp_squared | 0 | | 0 | -1.23 | | .243 | 0 | | 0 | |  |
| lag_health_expendi~e | -1.499 | | .707 | -2.12 | | .057 | -3.054 | | .056 | | * |
| Constant | 527.372 | | 605.041 | 0.87 | | .402 | -804.314 | | 1859.059 | |  |
|  | | | | | | | | | | | |
| Mean dependent var | | 74.795 | | | SD dependent var | | | 11.223 | |  |  |
| Number of obs | | 21 | | | F-test | | | 758.156 | |  |  |
| **** p<.01, ** p<.05, * p<.1* | | | | | | | | | | | |
|  | | | | | | | | | | | |

**MLR Results for Country ID: 28 - Malawi**

| mortality_u5 | Coef. | | St.Err. | t-value | | p-value | [95% Conf | | Interval] | | Sig |
| --- | --- | --- | --- | --- | --- | --- | --- | --- | --- | --- | --- |
| imm_dtp | .401 | | .188 | 2.14 | | .056 | -.012 | | .814 | | * |
| malaria_inc | .079 | | .042 | 1.90 | | .083 | -.012 | | .171 | | * |
| adolescent_fert | -.201 | | 1.405 | -0.14 | | .889 | -3.294 | | 2.892 | |  |
| fert_rate | 40.068 | | 15.509 | 2.58 | | .025 | 5.933 | | 74.202 | | ** |
| ln_basic_sanitation | -38.157 | | 10.367 | -3.68 | | .004 | -60.975 | | -15.339 | | *** |
| adolescent_fert_sq~d | -.008 | | .005 | -1.53 | | .154 | -.019 | | .003 | |  |
| lag_gdp_per_capita | -.14 | | .044 | -3.16 | | .009 | -.238 | | -.043 | | *** |
| lag_gdp_squared | 0 | | 0 | 2.00 | | .071 | 0 | | 0 | | * |
| lag_health_expendi~e | -1.848 | | 1.124 | -1.64 | | .128 | -4.322 | | .626 | |  |
| Constant | 175.571 | | 87.376 | 2.01 | | .07 | -16.742 | | 367.884 | | * |
|  | | | | | | | | | | | |
| Mean dependent var | | 82.910 | | | SD dependent var | | | 35.127 | |  |  |
| Number of obs | | 21 | | | F-test | | | 1458.581 | |  |  |
| **** p<.01, ** p<.05, * p<.1* | | | | | | | | | | | |
|  | | | | | | | | | | | |

**MLR Results for Country ID: 29 - Mali**

| mortality_u5 | Coef. | | St.Err. | t-value | | p-value | [95% Conf | | Interval] | | Sig |
| --- | --- | --- | --- | --- | --- | --- | --- | --- | --- | --- | --- |
| imm_dtp | .015 | | .013 | 1.12 | | .285 | -.014 | | .044 | |  |
| malaria_inc | 0 | | .003 | -0.10 | | .926 | -.007 | | .007 | |  |
| adolescent_fert | -.79 | | 1.589 | -0.50 | | .629 | -4.286 | | 2.706 | |  |
| fert_rate | 4.427 | | 6.228 | 0.71 | | .492 | -9.28 | | 18.135 | |  |
| ln_basic_sanitation | -75.23 | | 1.953 | -38.53 | | 0 | -79.528 | | -70.932 | | *** |
| adolescent_fert_sq~d | .002 | | .005 | 0.47 | | .65 | -.008 | | .012 | |  |
| lag_gdp_per_capita | -.015 | | .004 | -3.85 | | .003 | -.024 | | -.007 | | *** |
| lag_gdp_squared | 0 | | 0 | 3.80 | | .003 | 0 | | 0 | | *** |
| lag_health_expendi~e | -.012 | | .191 | -0.06 | | .952 | -.432 | | .409 | |  |
| Constant | 437.432 | | 88.97 | 4.92 | | 0 | 241.611 | | 633.253 | | *** |
|  | | | | | | | | | | | |
| Mean dependent var | | 133.395 | | | SD dependent var | | | 25.769 | |  |  |
| Number of obs | | 21 | | | F-test | | | 292521.668 | |  |  |
| **** p<.01, ** p<.05, * p<.1* | | | | | | | | | | | |
|  | | | | | | | | | | | |

**MLR Results for Country ID: 30 - Mauritania**

| mortality_u5 | Coef. | | St.Err. | t-value | | p-value | [95% Conf | | Interval] | | Sig |
| --- | --- | --- | --- | --- | --- | --- | --- | --- | --- | --- | --- |
| imm_dtp | .001 | | .053 | 0.02 | | .982 | -.116 | | .118 | |  |
| malaria_inc | .034 | | .015 | 2.21 | | .049 | 0 | | .068 | | ** |
| adolescent_fert | 19.211 | | 7.861 | 2.44 | | .033 | 1.909 | | 36.513 | | ** |
| fert_rate | 11.86 | | 9.025 | 1.31 | | .216 | -8.004 | | 31.724 | |  |
| ln_basic_sanitation | -54.637 | | 12.384 | -4.41 | | .001 | -81.894 | | -27.379 | | *** |
| adolescent_fert_sq~d | -.107 | | .041 | -2.62 | | .024 | -.198 | | -.017 | | ** |
| lag_gdp_per_capita | .027 | | .019 | 1.40 | | .19 | -.016 | | .07 | |  |
| lag_gdp_squared | 0 | | 0 | -1.32 | | .215 | 0 | | 0 | |  |
| lag_health_expendi~e | -2.227 | | 1.427 | -1.56 | | .147 | -5.369 | | .914 | |  |
| Constant | -679.033 | | 326.311 | -2.08 | | .062 | -1397.238 | | 39.172 | | * |
|  | | | | | | | | | | | |
| Mean dependent var | | 59.024 | | | SD dependent var | | | 15.626 | |  |  |
| Number of obs | | 21 | | | F-test | | | 2581.601 | |  |  |
| **** p<.01, ** p<.05, * p<.1* | | | | | | | | | | | |
|  | | | | | | | | | | | |

**MLR Results for Country ID: 31 - Morocco**

| mortality_u5 | Coef. | | St.Err. | t-value | | p-value | [95% Conf | | Interval] | | Sig |
| --- | --- | --- | --- | --- | --- | --- | --- | --- | --- | --- | --- |
| imm_dtp | .107 | | .031 | 3.40 | | .005 | .038 | | .175 | | *** |
| o | 0 | | . | . | | . | . | | . | |  |
| adolescent_fert | -3.819 | | .533 | -7.17 | | 0 | -4.98 | | -2.658 | | *** |
| fert_rate | 52.143 | | 5.356 | 9.73 | | 0 | 40.473 | | 63.813 | | *** |
| ln_basic_sanitation | -122.638 | | 3.987 | -30.76 | | 0 | -131.326 | | -113.951 | | *** |
| adolescent_fert_sq~d | .014 | | .011 | 1.30 | | .219 | -.009 | | .037 | |  |
| lag_gdp_per_capita | -.01 | | .002 | -6.55 | | 0 | -.014 | | -.007 | | *** |
| lag_gdp_squared | 0 | | 0 | 6.28 | | 0 | 0 | | 0 | | *** |
| lag_health_expendi~e | -.743 | | .119 | -6.25 | | 0 | -1.002 | | -.484 | | *** |
| Constant | 547.476 | | 28.78 | 19.02 | | 0 | 484.769 | | 610.182 | | *** |
|  | | | | | | | | | | | |
| Mean dependent var | | 31.157 | | | SD dependent var | | | 10.338 | |  |  |
| Number of obs | | 21 | | | F-test | | | 271141.306 | |  |  |
| **** p<.01, ** p<.05, * p<.1* | | | | | | | | | | | |
|  | | | | | | | | | | | |

**MLR Results for Country ID: 32 - Mozambique**

| mortality_u5 | Coef. | | St.Err. | t-value | | p-value | [95% Conf | | Interval] | | Sig |
| --- | --- | --- | --- | --- | --- | --- | --- | --- | --- | --- | --- |
| imm_dtp | -.27 | | .055 | -4.90 | | 0 | -.392 | | -.149 | | *** |
| malaria_inc | .062 | | .031 | 2.04 | | .067 | -.005 | | .13 | | * |
| adolescent_fert | 16.335 | | 12.49 | 1.31 | | .218 | -11.156 | | 43.826 | |  |
| fert_rate | -19.713 | | 7.271 | -2.71 | | .02 | -35.716 | | -3.71 | | ** |
| ln_basic_sanitation | -84.813 | | 11.636 | -7.29 | | 0 | -110.424 | | -59.201 | | *** |
| adolescent_fert_sq~d | -.05 | | .038 | -1.30 | | .219 | -.134 | | .034 | |  |
| lag_gdp_per_capita | -.018 | | .05 | -0.35 | | .734 | -.129 | | .094 | |  |
| lag_gdp_squared | 0 | | 0 | 0.31 | | .765 | 0 | | 0 | |  |
| lag_health_expendi~e | 1.243 | | 1.337 | 0.93 | | .373 | -1.7 | | 4.185 | |  |
| Constant | -877.854 | | 981.19 | -0.89 | | .39 | -3037.439 | | 1281.731 | |  |
|  | | | | | | | | | | | |
| Mean dependent var | | 97.500 | | | SD dependent var | | | 28.219 | |  |  |
| Number of obs | | 21 | | | F-test | | | 1293.378 | |  |  |
| **** p<.01, ** p<.05, * p<.1* | | | | | | | | | | | |
|  | | | | | | | | | | | |

**MLR Results for Country ID: 33 - Namibia**

| mortality_u5 | Coef. | | St.Err. | t-value | | p-value | [95% Conf | | Interval] | | Sig |
| --- | --- | --- | --- | --- | --- | --- | --- | --- | --- | --- | --- |
| imm_dtp | .195 | | .184 | 1.06 | | .312 | -.21 | | .599 | |  |
| malaria_inc | .14 | | .022 | 6.41 | | 0 | .092 | | .188 | | *** |
| adolescent_fert | 7.104 | | 4.444 | 1.60 | | .138 | -2.678 | | 16.885 | |  |
| fert_rate | 22.022 | | 17.851 | 1.23 | | .243 | -17.267 | | 61.311 | |  |
| ln_basic_sanitation | -118.374 | | 22.471 | -5.27 | | 0 | -167.831 | | -68.916 | | *** |
| adolescent_fert_sq~d | -.056 | | .028 | -1.98 | | .073 | -.118 | | .006 | | * |
| lag_gdp_per_capita | -.012 | | .002 | -5.26 | | 0 | -.017 | | -.007 | | *** |
| lag_gdp_squared | 0 | | 0 | 6.14 | | 0 | 0 | | 0 | | *** |
| lag_health_expendi~e | .905 | | .715 | 1.27 | | .232 | -.669 | | 2.479 | |  |
| Constant | 164.458 | | 201.639 | 0.82 | | .432 | -279.347 | | 608.263 | |  |
|  | | | | | | | | | | | |
| Mean dependent var | | 58.133 | | | SD dependent var | | | 10.498 | |  |  |
| Number of obs | | 21 | | | F-test | | | 549.427 | |  |  |
| **** p<.01, ** p<.05, * p<.1* | | | | | | | | | | | |
|  | | | | | | | | | | | |

**MLR Results for Country ID: 34 - Niger**

| mortality_u5 | Coef. | | St.Err. | t-value | | p-value | [95% Conf | | Interval] | | Sig |
| --- | --- | --- | --- | --- | --- | --- | --- | --- | --- | --- | --- |
| imm_dtp | -.007 | | .057 | -0.11 | | .911 | -.133 | | .12 | |  |
| malaria_inc | .029 | | .007 | 3.95 | | .002 | .013 | | .045 | | *** |
| adolescent_fert | 3.265 | | .513 | 6.37 | | 0 | 2.136 | | 4.393 | | *** |
| fert_rate | -36.812 | | 3.064 | -12.01 | | 0 | -43.556 | | -30.069 | | *** |
| ln_basic_sanitation | -135.542 | | 6.083 | -22.28 | | 0 | -148.93 | | -122.153 | | *** |
| adolescent_fert_sq~d | -.009 | | .001 | -6.67 | | 0 | -.011 | | -.006 | | *** |
| lag_gdp_per_capita | -.2 | | .015 | -13.26 | | 0 | -.233 | | -.167 | | *** |
| lag_gdp_squared | 0 | | 0 | 13.62 | | 0 | 0 | | 0 | | *** |
| lag_health_expendi~e | -.25 | | .656 | -0.38 | | .71 | -1.693 | | 1.193 | |  |
| Constant | 462.824 | | 27.664 | 16.73 | | 0 | 401.936 | | 523.712 | | *** |
|  | | | | | | | | | | | |
| Mean dependent var | | 147.243 | | | SD dependent var | | | 30.288 | |  |  |
| Number of obs | | 21 | | | F-test | | | 71507.866 | |  |  |
| **** p<.01, ** p<.05, * p<.1* | | | | | | | | | | | |
|  | | | | | | | | | | | |

**MLR Results for Country ID: 35 - Nigeria**

| mortality_u5 | Coef. | | St.Err. | t-value | | p-value | [95% Conf | | Interval] | | Sig |
| --- | --- | --- | --- | --- | --- | --- | --- | --- | --- | --- | --- |
| imm_dtp | .08 | | .041 | 1.95 | | .077 | -.01 | | .17 | | * |
| malaria_inc | -.062 | | .036 | -1.72 | | .114 | -.142 | | .017 | |  |
| adolescent_fert | 3.076 | | 1.963 | 1.57 | | .145 | -1.244 | | 7.396 | |  |
| fert_rate | -35.186 | | 11.499 | -3.06 | | .011 | -60.495 | | -9.878 | | ** |
| ln_basic_sanitation | -237.555 | | 26.705 | -8.90 | | 0 | -296.333 | | -178.777 | | *** |
| adolescent_fert_sq~d | -.013 | | .008 | -1.69 | | .119 | -.03 | | .004 | |  |
| lag_gdp_per_capita | -.003 | | .005 | -0.66 | | .522 | -.013 | | .007 | |  |
| lag_gdp_squared | 0 | | 0 | 0.78 | | .454 | 0 | | 0 | |  |
| lag_health_expendi~e | -1.02 | | .492 | -2.07 | | .062 | -2.103 | | .063 | | * |
| Constant | 1030.11 | | 141.11 | 7.30 | | 0 | 719.53 | | 1340.691 | | *** |
|  | | | | | | | | | | | |
| Mean dependent var | | 137.867 | | | SD dependent var | | | 18.849 | |  |  |
| Number of obs | | 21 | | | F-test | | | 5136.808 | |  |  |
| **** p<.01, ** p<.05, * p<.1* | | | | | | | | | | | |
|  | | | | | | | | | | | |

**MLR Results for Country ID: 36 - Rwanda**

| mortality_u5 | Coef. | | St.Err. | t-value | | p-value | [95% Conf | | Interval] | | Sig |
| --- | --- | --- | --- | --- | --- | --- | --- | --- | --- | --- | --- |
| imm_dtp | -.854 | | .286 | -2.99 | | .012 | -1.483 | | -.225 | | ** |
| malaria_inc | .01 | | .005 | 2.14 | | .056 | 0 | | .021 | | * |
| adolescent_fert | -12.574 | | 5.59 | -2.25 | | .046 | -24.878 | | -.27 | | ** |
| fert_rate | -9.965 | | 12.702 | -0.78 | | .449 | -37.921 | | 17.992 | |  |
| ln_basic_sanitation | -253.55 | | 84.926 | -2.99 | | .012 | -440.47 | | -66.629 | | ** |
| adolescent_fert_sq~d | .158 | | .082 | 1.93 | | .08 | -.022 | | .339 | | * |
| lag_gdp_per_capita | -.192 | | .064 | -3.01 | | .012 | -.333 | | -.052 | | ** |
| lag_gdp_squared | 0 | | 0 | 5.02 | | 0 | 0 | | 0 | | *** |
| lag_health_expendi~e | -3.197 | | .629 | -5.08 | | 0 | -4.581 | | -1.813 | | *** |
| Constant | 1556.135 | | 354.604 | 4.39 | | .001 | 775.657 | | 2336.614 | | *** |
|  | | | | | | | | | | | |
| Mean dependent var | | 75.448 | | | SD dependent var | | | 38.196 | |  |  |
| Number of obs | | 21 | | | F-test | | | 1713.673 | |  |  |
| **** p<.01, ** p<.05, * p<.1* | | | | | | | | | | | |
|  | | | | | | | | | | | |

**MLR Results for Country ID: 37 - Sao Tome And principe**

| mortality_u5 | Coef. | | St.Err. | t-value | | p-value | [95% Conf | | Interval] | | Sig |
| --- | --- | --- | --- | --- | --- | --- | --- | --- | --- | --- | --- |
| imm_dtp | -.261 | | .057 | -4.54 | | .001 | -.387 | | -.135 | | *** |
| malaria_inc | .001 | | .002 | 0.59 | | .57 | -.003 | | .006 | |  |
| adolescent_fert | -.258 | | .34 | -0.76 | | .465 | -1.007 | | .491 | |  |
| fert_rate | -5.204 | | 3.139 | -1.66 | | .126 | -12.113 | | 1.704 | |  |
| ln_basic_sanitation | -94.763 | | 7.361 | -12.87 | | 0 | -110.966 | | -78.56 | | *** |
| adolescent_fert_sq~d | .001 | | .001 | 0.60 | | .563 | -.002 | | .004 | |  |
| lag_gdp_per_capita | -.008 | | .003 | -2.61 | | .024 | -.015 | | -.001 | | ** |
| lag_gdp_squared | 0 | | 0 | 5.32 | | 0 | 0 | | 0 | | *** |
| lag_health_expendi~e | -.218 | | .131 | -1.66 | | .124 | -.507 | | .07 | |  |
| Constant | 441.863 | | 23.05 | 19.17 | | 0 | 391.131 | | 492.595 | | *** |
|  | | | | | | | | | | | |
| Mean dependent var | | 37.762 | | | SD dependent var | | | 19.328 | |  |  |
| Number of obs | | 21 | | | F-test | | | 11624.182 | |  |  |
| **** p<.01, ** p<.05, * p<.1* | | | | | | | | | | | |
|  | | | | | | | | | | | |

**MLR Results for Country ID: 38 - Senegal**

| mortality_u5 | Coef. | | St.Err. | t-value | | p-value | [95% Conf | | Interval] | | Sig |
| --- | --- | --- | --- | --- | --- | --- | --- | --- | --- | --- | --- |
| imm_dtp | -.117 | | .081 | -1.44 | | .178 | -.297 | | .062 | |  |
| malaria_inc | .008 | | .017 | 0.49 | | .634 | -.029 | | .045 | |  |
| adolescent_fert | -3.645 | | 3.133 | -1.16 | | .269 | -10.54 | | 3.25 | |  |
| fert_rate | 5.555 | | 7.957 | 0.70 | | .5 | -11.959 | | 23.068 | |  |
| ln_basic_sanitation | -162.948 | | 29.385 | -5.55 | | 0 | -227.624 | | -98.271 | | *** |
| adolescent_fert_sq~d | .017 | | .017 | 1.01 | | .333 | -.02 | | .055 | |  |
| lag_gdp_per_capita | -.079 | | .034 | -2.35 | | .039 | -.153 | | -.005 | | ** |
| lag_gdp_squared | 0 | | 0 | 2.10 | | .059 | 0 | | 0 | | * |
| lag_health_expendi~e | -.499 | | .94 | -0.53 | | .606 | -2.567 | | 1.569 | |  |
| Constant | 914.662 | | 179.764 | 5.09 | | 0 | 519.004 | | 1310.32 | | *** |
|  | | | | | | | | | | | |
| Mean dependent var | | 68.681 | | | SD dependent var | | | 23.653 | |  |  |
| Number of obs | | 21 | | | F-test | | | 2306.452 | |  |  |
| **** p<.01, ** p<.05, * p<.1* | | | | | | | | | | | |
|  | | | | | | | | | | | |

**MLR Results for Country ID: 39 - Sierra leone**

| mortality_u5 | Coef. | | St.Err. | t-value | | p-value | [95% Conf | | Interval] | | Sig |
| --- | --- | --- | --- | --- | --- | --- | --- | --- | --- | --- | --- |
| imm_dtp | .183 | | .244 | 0.75 | | .468 | -.353 | | .719 | |  |
| malaria_inc | -.191 | | .119 | -1.60 | | .137 | -.454 | | .072 | |  |
| adolescent_fert | -.685 | | .61 | -1.12 | | .285 | -2.027 | | .657 | |  |
| fert_rate | 72.793 | | 45.176 | 1.61 | | .135 | -26.639 | | 172.225 | |  |
| ln_basic_sanitation | -18.467 | | 56.673 | -0.33 | | .751 | -143.203 | | 106.268 | |  |
| adolescent_fert_sq~d | 0 | | .002 | 0.19 | | .85 | -.004 | | .005 | |  |
| lag_gdp_per_capita | -.04 | | .031 | -1.28 | | .228 | -.109 | | .029 | |  |
| lag_gdp_squared | 0 | | 0 | 1.52 | | .158 | 0 | | 0 | |  |
| lag_health_expendi~e | .242 | | .319 | 0.76 | | .464 | -.46 | | .943 | |  |
| Constant | -20.759 | | 302.59 | -0.07 | | .947 | -686.755 | | 645.237 | |  |
|  | | | | | | | | | | | |
| Mean dependent var | | 156.105 | | | SD dependent var | | | 37.686 | |  |  |
| Number of obs | | 21 | | | F-test | | | 2459.471 | |  |  |
| **** p<.01, ** p<.05, * p<.1* | | | | | | | | | | | |
|  | | | | | | | | | | | |

**MLR Results for Country ID: 40 - South Africa**

| mortality_u5 | Coef. | | St.Err. | t-value | | p-value | [95% Conf | | Interval] | | Sig |
| --- | --- | --- | --- | --- | --- | --- | --- | --- | --- | --- | --- |
| imm_dtp | .457 | | .556 | 0.82 | | .429 | -.767 | | 1.681 | |  |
| malaria_inc | -1.27 | | .554 | -2.29 | | .043 | -2.489 | | -.051 | | ** |
| adolescent_fert | -2.577 | | 1.597 | -1.61 | | .135 | -6.091 | | .937 | |  |
| fert_rate | 23.13 | | 8.297 | 2.79 | | .018 | 4.869 | | 41.391 | | ** |
| ln_basic_sanitation | -185.219 | | 29.28 | -6.33 | | 0 | -249.665 | | -120.773 | | *** |
| adolescent_fert_sq~d | .018 | | .014 | 1.31 | | .217 | -.012 | | .048 | |  |
| lag_gdp_per_capita | -.01 | | .011 | -0.97 | | .353 | -.034 | | .013 | |  |
| lag_gdp_squared | 0 | | 0 | 0.66 | | .522 | 0 | | 0 | |  |
| lag_health_expendi~e | -1.599 | | 1.45 | -1.10 | | .294 | -4.791 | | 1.593 | |  |
| Constant | 886.628 | | 119.216 | 7.44 | | 0 | 624.236 | | 1149.02 | | *** |
|  | | | | | | | | | | | |
| Mean dependent var | | 53.757 | | | SD dependent var | | | 18.710 | |  |  |
| Number of obs | | 21 | | | F-test | | | 124.549 | |  |  |
| **** p<.01, ** p<.05, * p<.1* | | | | | | | | | | | |
|  | | | | | | | | | | | |

**MLR Results for Country ID: 41 - Sudan**

| mortality_u5 | Coef. | | St.Err. | t-value | | p-value | [95% Conf | | Interval] | | Sig |
| --- | --- | --- | --- | --- | --- | --- | --- | --- | --- | --- | --- |
| imm_dtp | .567 | | .361 | 1.57 | | .144 | -.227 | | 1.362 | |  |
| malaria_inc | -.07 | | .116 | -0.61 | | .556 | -.325 | | .184 | |  |
| adolescent_fert | -13.465 | | 7.139 | -1.89 | | .086 | -29.178 | | 2.248 | | * |
| fert_rate | 78.483 | | 30.381 | 2.58 | | .025 | 11.616 | | 145.35 | | ** |
| ln_basic_sanitation | -78.547 | | 38.636 | -2.03 | | .067 | -163.585 | | 6.49 | | * |
| adolescent_fert_sq~d | .066 | | .036 | 1.83 | | .094 | -.013 | | .144 | | * |
| lag_gdp_per_capita | .006 | | .026 | 0.22 | | .828 | -.052 | | .064 | |  |
| lag_gdp_squared | 0 | | 0 | -0.20 | | .846 | 0 | | 0 | |  |
| lag_health_expendi~e | .498 | | 1.05 | 0.47 | | .645 | -1.813 | | 2.809 | |  |
| Constant | 557.47 | | 315.642 | 1.77 | | .105 | -137.253 | | 1252.193 | |  |
|  | | | | | | | | | | | |
| Mean dependent var | | 75.081 | | | SD dependent var | | | 15.785 | |  |  |
| Number of obs | | 21 | | | F-test | | | 244.740 | |  |  |
| **** p<.01, ** p<.05, * p<.1* | | | | | | | | | | | |
|  | | | | | | | | | | | |

**MLR Results for Country ID: 42 - Tanzania**

| mortality_u5 | Coef. | | St.Err. | t-value | | p-value | [95% Conf | | Interval] | | Sig |
| --- | --- | --- | --- | --- | --- | --- | --- | --- | --- | --- | --- |
| imm_dtp | .059 | | .06 | 0.98 | | .347 | -.073 | | .191 | |  |
| malaria_inc | -.018 | | .03 | -0.61 | | .553 | -.084 | | .048 | |  |
| adolescent_fert | .267 | | 1.164 | 0.23 | | .823 | -2.296 | | 2.829 | |  |
| fert_rate | -15.274 | | 9.4 | -1.62 | | .132 | -35.963 | | 5.415 | |  |
| ln_basic_sanitation | -58.955 | | 2.496 | -23.62 | | 0 | -64.45 | | -53.461 | | *** |
| adolescent_fert_sq~d | 0 | | .005 | 0.07 | | .949 | -.011 | | .011 | |  |
| lag_gdp_per_capita | -.018 | | .026 | -0.70 | | .496 | -.076 | | .039 | |  |
| lag_gdp_squared | 0 | | 0 | 0.30 | | .768 | 0 | | 0 | |  |
| lag_health_expendi~e | -.163 | | .563 | -0.29 | | .777 | -1.402 | | 1.076 | |  |
| Constant | 293.224 | | 91.968 | 3.19 | | .009 | 90.803 | | 495.644 | | *** |
|  | | | | | | | | | | | |
| Mean dependent var | | 71.305 | | | SD dependent var | | | 23.666 | |  |  |
| Number of obs | | 21 | | | F-test | | | 28659.969 | |  |  |
| **** p<.01, ** p<.05, * p<.1* | | | | | | | | | | | |
|  | | | | | | | | | | | |

**MLR Results for Country ID: 43 - Togo**

| mortality_u5 | Coef. | | St.Err. | t-value | | p-value | [95% Conf | | Interval] | | Sig |
| --- | --- | --- | --- | --- | --- | --- | --- | --- | --- | --- | --- |
| imm_dtp | -.059 | | .054 | -1.10 | | .295 | -.178 | | .059 | |  |
| malaria_inc | -.001 | | .003 | -0.18 | | .862 | -.008 | | .007 | |  |
| adolescent_fert | -1.382 | | 3.578 | -0.39 | | .707 | -9.257 | | 6.492 | |  |
| fert_rate | .725 | | 1.158 | 0.63 | | .544 | -1.823 | | 3.274 | |  |
| ln_basic_sanitation | -70.253 | | 6.891 | -10.20 | | 0 | -85.42 | | -55.087 | | *** |
| adolescent_fert_sq~d | .009 | | .02 | 0.43 | | .674 | -.036 | | .054 | |  |
| lag_gdp_per_capita | -.003 | | .011 | -0.31 | | .766 | -.027 | | .021 | |  |
| lag_gdp_squared | 0 | | 0 | 0.09 | | .931 | 0 | | 0 | |  |
| lag_health_expendi~e | -.063 | | .555 | -0.11 | | .912 | -1.285 | | 1.159 | |  |
| Constant | 328.904 | | 163.189 | 2.02 | | .069 | -30.274 | | 688.081 | | * |
|  | | | | | | | | | | | |
| Mean dependent var | | 86.957 | | | SD dependent var | | | 16.622 | |  |  |
| Number of obs | | 21 | | | F-test | | | 16101.897 | |  |  |
| **** p<.01, ** p<.05, * p<.1* | | | | | | | | | | | |
|  | | | | | | | | | | | |

**MLR Results for Country ID: 44 - Uganda**

| mortality_u5 | Coef. | | St.Err. | t-value | | p-value | [95% Conf | | Interval] | | Sig |
| --- | --- | --- | --- | --- | --- | --- | --- | --- | --- | --- | --- |
| imm_dtp | -.139 | | .086 | -1.61 | | .136 | -.328 | | .051 | |  |
| malaria_inc | .016 | | .014 | 1.18 | | .264 | -.014 | | .046 | |  |
| adolescent_fert | -3.888 | | 1.054 | -3.69 | | .004 | -6.207 | | -1.569 | | *** |
| fert_rate | -7.131 | | 9.393 | -0.76 | | .464 | -27.803 | | 13.542 | |  |
| ln_basic_sanitation | -474.63 | | 120.483 | -3.94 | | .002 | -739.811 | | -209.45 | | *** |
| adolescent_fert_sq~d | .014 | | .004 | 3.42 | | .006 | .005 | | .022 | | *** |
| lag_gdp_per_capita | -.056 | | .012 | -4.59 | | .001 | -.083 | | -.029 | | *** |
| lag_gdp_squared | 0 | | 0 | 4.47 | | .001 | 0 | | 0 | | *** |
| lag_health_expendi~e | -.349 | | .441 | -0.79 | | .445 | -1.32 | | .621 | |  |
| Constant | 1805.402 | | 375.189 | 4.81 | | .001 | 979.616 | | 2631.188 | | *** |
|  | | | | | | | | | | | |
| Mean dependent var | | 78.448 | | | SD dependent var | | | 30.636 | |  |  |
| Number of obs | | 21 | | | F-test | | | 8659.720 | |  |  |
| **** p<.01, ** p<.05, * p<.1* | | | | | | | | | | | |
|  | | | | | | | | | | | |

**MLR Results for Country ID: 45 - Zambia**

| mortality_u5 | Coef. | | St.Err. | t-value | | p-value | [95% Conf | | Interval] | | Sig |
| --- | --- | --- | --- | --- | --- | --- | --- | --- | --- | --- | --- |
| imm_dtp | -.192 | | .071 | -2.71 | | .02 | -.349 | | -.036 | | ** |
| malaria_inc | -.042 | | .028 | -1.51 | | .158 | -.103 | | .019 | |  |
| adolescent_fert | -1.412 | | 2.254 | -0.63 | | .544 | -6.375 | | 3.55 | |  |
| fert_rate | -52.262 | | 12.361 | -4.23 | | .001 | -79.468 | | -25.055 | | *** |
| ln_basic_sanitation | -303.891 | | 42.572 | -7.14 | | 0 | -397.592 | | -210.19 | | *** |
| adolescent_fert_sq~d | .008 | | .008 | 0.97 | | .352 | -.01 | | .025 | |  |
| lag_gdp_per_capita | .016 | | .013 | 1.19 | | .26 | -.013 | | .045 | |  |
| lag_gdp_squared | 0 | | 0 | -0.96 | | .358 | 0 | | 0 | |  |
| lag_health_expendi~e | .296 | | .622 | 0.48 | | .644 | -1.073 | | 1.664 | |  |
| Constant | 1436.631 | | 130.538 | 11.01 | | 0 | 1149.319 | | 1723.943 | | *** |
|  | | | | | | | | | | | |
| Mean dependent var | | 81.524 | | | SD dependent var | | | 28.243 | |  |  |
| Number of obs | | 21 | | | F-test | | | 8838.823 | |  |  |
| Source: Authors’ compilation | | | | | | | | | | | |
| **_Note:_** _*** p<0.01, ** p<0.05, * p<0.1. mortality_u5: Under-five mortality rate (per 1,000 live births); imm_dtp: Diphtheria-Tetanus-Pertussis immunization coverage (% of children ages 12–23 months); malaria_inc: Incidence of malaria (new cases per 1,000 population at risk); adolescent_fert: Adolescent fertility rate (births per 1,000 women ages 15–19); fert_rate: Total fertility rate (births per woman); ln_basic_sanitation: Natural log of the percentage of the population using at least basic sanitation services; lag_gdp_per_capita: One-year lagged Gross Domestic Product per capita (current US$); lag_gdp_squared: Square of lagged GDP per capita (to capture diminishing marginal returns); lag_health_expenditure: One-year lagged current health expenditure (% of GDP); adolescent_fert_sq: Square of the adolescent fertility rate (to capture non-linear demographic risks). Results are descriptive illustrations of national heterogeneity across the 2000–2021 study period. These results are provided to illustrate heterogeneity across the 45-country panel. Due to multiple testing and a limited time dimension for individual units, these coefficients are descriptive in nature and should not be used for inferential country-level rankings._ | | | | | | | | | | | |
