# Supplementary material for: Determinants of under-five mortality in Africa: evidence from a two-decade panel analysis for public health policy
Source: Arch Public Health. 2026 Jun 17;84:193. doi: 10.1186/s13690-026-01994-0 (PMC13520361; doi:10.1186/s13690-026-01994-0)
Supplement: Supplementary file 2 [file 13690_2026_1994_MOESM2_ESM.docx]

**Appendix 2: Comparative analysis of sample selection (Descriptive summary statistics and model sensitivity for primary balanced panel and full dataset)**

**Comparison of balanced sample and full sample (Descriptive statistics)**

**Table 2.A: Comparison of excluded and included countries (Means of the considered variables) for the period 2000-2021**

| Variable | Included Countries (Mean) | Excluded Countries (Mean) |
| --- | --- | --- |
| U5MR | 88.33 | 84.80 |
| GDP | 1808.85 | 2229.17 |
| DTP | 76.15 | 77.51 |
| IOM | 212.63 | 205.06 |
| CHE | 5.00 | 5.08 |
| AFR | 106.41 | 100.07 |
| TFR | 4.85 | 4.65 |
| BSS | 35.00 | 38.60 |

Source: Authors’ compilation

**Note:** GDP: Gross Domestic Product per capita (lagged); DTP: Diphtheria-Tetanus-Pertussis immunisation; IOM: Incidence of Malaria; CHE: Current Health Expenditure (lagged); AFR: Adolescent Fertility Rate; TFR: Total Fertility Rate; ln BSS: Natural log of Basic Sanitation Services access*.* U5MR is measured per 1,000 live births; GDP per capita is in current US$, DTP is measured in Share of one-year-olds vaccinated against diphtheria, tetanus and pertussis, IOM is measured in number of new cases of malaria in a year per 1,000 population at risk, CHE is measured in Current health expenditure % of GDP, AFR is measured in number of births per 1,000 women ages 15-19, TFR is measured in total births per woman, BSS is measured in people using at least basic sanitation services as a % of population. This table presents the mean values derived using descriptive statistical analysis of the comprehensive dataset for 45 African nations vs all 54 African nations sourced from the WDI for the period 2000–2021.

**Balanced sample analysis (N=45)**

**Table 2.B: Final regional panel regression results (Fixed Effects Model) identifying associations with under-five mortality across the 45-African countries balanced panel (2000–2021) (N=45)**

| mortality_u5 | Coef. | | St.Err. | t-value | | p-value | [95% Conf | | Interval] | | Sig |
| --- | --- | --- | --- | --- | --- | --- | --- | --- | --- | --- | --- |
| imm_dtp | -.454 | | .109 | -4.18 | | 0 | -.673 | | -.235 | | *** |
| malaria_inc | .052 | | .025 | 2.09 | | .043 | .002 | | .102 | | ** |
| adolescent_fert | -.752 | | .338 | -2.23 | | .031 | -1.433 | | -.072 | | ** |
| fert_rate | 29.757 | | 6.82 | 4.36 | | 0 | 16.012 | | 43.502 | | *** |
| ln_basic_sanitation | -19.02 | | 6.8 | -2.80 | | .008 | -32.724 | | -5.315 | | *** |
| adolescent_fert_sq~d | .002 | | .001 | 1.74 | | .089 | 0 | | .005 | | * |
| lag_gdp_per_capita | -.007 | | .002 | -3.93 | | 0 | -.011 | | -.003 | | *** |
| lag_gdp_squared | 0 | | 0 | 3.40 | | .001 | 0 | | 0 | | *** |
| lag_health_expendi~e | -.774 | | .599 | -1.29 | | .203 | -1.982 | | .434 | |  |
| Constant | 95.858 | | 39.722 | 2.41 | | .02 | 15.803 | | 175.912 | | ** |
|  | | | | | | | | | | | |
| Mean dependent var | | 86.305 | | | SD dependent var | | | 42.984 | |  |  |
| R-squared | | 0.667 | | | Number of obs | | | 945 | |  |  |
| F-test | | 97.312 | | | Prob > F | | | 0.000 | |  |  |
| Akaike crit. (AIC) | | 7745.074 | | | Bayesian crit. (BIC) | | | 7788.735 | |  |  |
| Source: Authors’ compilation  **Note:** **** p<.01, ** p<.05, * p<.1*. mortality_u5: Under-five mortality rate (per 1,000 live births); imm_dtp: Diphtheria-Tetanus-Pertussis immunisation coverage (% of children ages 12–23 months); malaria_inc: Incidence of malaria (new cases per 1,000 population at risk); adolescent_fert: Adolescent fertility rate (births per 1,000 women ages 15–19); fert_rate: Total fertility rate (births per woman); ln_basic_sanitation: Natural log of the percentage of the population using at least basic sanitation services; lag_gdp_per_capita: One-year lagged Gross Domestic Product per capita (current US$); lag_gdp_squared: Square of lagged GDP per capita (to capture diminishing marginal returns); lag_health_expenditure: One-year lagged current health expenditure (% of GDP); adolescent_fert_sq: Square of the adolescent fertility rate (to capture non-linear demographic risks). This table presents the final results of the preferred specification for the balanced panel dataset within the period 2000–2021. | | | | | | | | | | | |

**Full sample analysis (N=54)**

**Table 2.C: Final regional panel regression results (Fixed Effects Model) identifying associations with under-five mortality across the 54-African countries unbalanced panel (2000–2021) (N=54)**

| mortality_u5 | Coef. | | St.Err. | t-value | | p-value | [95% Conf | | Interval] | | Sig |
| --- | --- | --- | --- | --- | --- | --- | --- | --- | --- | --- | --- |
| imm_dtp | -.45 | | .108 | -4.16 | | 0 | -.668 | | -.233 | | *** |
| malaria_inc | .052 | | .025 | 2.10 | | .041 | .002 | | .101 | | ** |
| adolescent_fert | -.756 | | .337 | -2.24 | | .03 | -1.434 | | -.077 | | ** |
| fert_rate | 29.923 | | 6.729 | 4.45 | | 0 | 16.386 | | 43.459 | | *** |
| ln_basic_sanitation | -18.8 | | 6.776 | -2.77 | | .008 | -32.431 | | -5.169 | | *** |
| adolescent_fert_sq~d | .002 | | .001 | 1.76 | | .085 | 0 | | .005 | | * |
| lag_gdp_per_capita | -.007 | | .002 | -4.03 | | 0 | -.011 | | -.004 | | *** |
| lag_gdp_squared | 0 | | 0 | 3.49 | | .001 | 0 | | 0 | | *** |
| lag_health_expendi~e | -.68 | | .579 | -1.17 | | .246 | -1.845 | | .485 | |  |
| Constant | 93.607 | | 39.381 | 2.38 | | .022 | 14.383 | | 172.832 | | ** |
|  | | | | | | | | | | | |
| Mean dependent var | | 86.082 | | | SD dependent var | | | 42.684 | |  |  |
| R-squared | | 0.667 | | | Number of obs | | | 976 | |  |  |
| F-test | | 95.798 | | | Prob > F | | | 0.000 | |  |  |
| Akaike crit. (AIC) | | 7974.195 | | | Bayesian crit. (BIC) | | | 8018.147 | |  |  |
| Source: Authors’ compilation  **Note:** **** p<.01, ** p<.05, * p<.1*. mortality_u5: Under-five mortality rate (per 1,000 live births); imm_dtp: Diphtheria-Tetanus-Pertussis immunisation coverage (% of children ages 12–23 months); malaria_inc: Incidence of malaria (new cases per 1,000 population at risk); adolescent_fert: Adolescent fertility rate (births per 1,000 women ages 15–19); fert_rate: Total fertility rate (births per woman); ln_basic_sanitation: Natural log of the percentage of the population using at least basic sanitation services; lag_gdp_per_capita: One-year lagged Gross Domestic Product per capita (current US$); lag_gdp_squared: Square of lagged GDP per capita (to capture diminishing marginal returns); lag_health_expenditure: One-year lagged current health expenditure (% of GDP); adolescent_fert_sq: Square of the adolescent fertility rate (to capture non-linear demographic risks). . This table presents the regression estimates utilising the complete dataset, which incorporates all African countries within the period 2000-2021 regardless of missing data points. | | | | | | | | | | | |
|  | | | | | | | | | | | |
