# Supplementary material for: Determinants of under-five mortality in Africa: evidence from a two-decade panel analysis for public health policy
Source: Arch Public Health. 2026 Jun 17;84:193. doi: 10.1186/s13690-026-01994-0 (PMC13520361; doi:10.1186/s13690-026-01994-0)
Supplement: Supplementary file 3 [file 13690_2026_1994_MOESM4_ESM.docx]

| **Code** | **Indicator Name** | **Unit of measure** |
| --- | --- | --- |
| SH.DYN.MORT | Mortality rate, under-5 (per 1,000 live births) | Per 1000 live births |
| NY.GDP.PCAP.CD | GDP per capita (current US$) | current US$ |
| CC.PER.RNK | Control of Corruption: Percentile Rank | Percentile |
| SH.IMM.IDPT | Immunisation, DPT (% of children ages 12-23 months) | Percentage |
| SH.IMM.MEAS | Immunisation, measles (% of children ages 12-23 months) | Percentage |
| SH.MLR.INCD.P3 | Incidence of malaria (per 1,000 population at risk) | Per 1000 population at risk |
| SH.XPD.CHEX.GD.ZS | Current health expenditure (% of GDP) | Percentage |
| SP.ADO.TFRT | Adolescent fertility rate (births per 1,000 women ages 15-19) | Per 1000 women |
| SP.DYN.TFRT.IN | Fertility rate, total (births per woman) | Births per woman |
| EN.ATM.PM25.MC.M3 | PM2.5 air pollution, mean annual exposure (micrograms per cubic meter) | microgram per cubic meter |
| EG.CFT.ACCS.ZS | Access to clean fuels and technologies for cooking (% of population) | % (share) of population |
| SH.STA.BASS.ZS | People using at least basic sanitation services (% of population) | % of population |

**Appendix 4: World Development Indicators codes for each variable**

**Table 4.A: World Development Indicators codes for each considered variable for the study**

Source: Authors’ compilation

**Note:** The variables utilised in this analysis are sourced from the World Bank World Development Indicators (WDI) database. The corresponding series codes are presented in the table.
