# Supplementary material for: Determinants of under-five mortality in Africa: evidence from a two-decade panel analysis for public health policy
Source: Arch Public Health. 2026 Jun 17;84:193. doi: 10.1186/s13690-026-01994-0 (PMC13520361; doi:10.1186/s13690-026-01994-0)
Supplement: Supplementary file 4 [file 13690_2026_1994_MOESM5_ESM.docx]

**Appendix 5: Descriptive summary statistics**

**Table 5.A: Descriptive summary statistics (Mean, SD, Min, Max) for under-five mortality and its determinants in 45 African nations (2000–2021)**

**Country: Algeria**

|  | **Mean** | **SD** | **N** | **Min** | **Max** |
| --- | --- | --- | --- | --- | --- |
| U5MR | 29.495 | 5.97 | 22 | 22.6 | 41.6 |
| GDP | 4124.39 | 1351.802 | 22 | 1772.929 | 6094.694 |
| IOM | .003 | .006 | 22 | 0 | .02 |
| CHE | 4.666 | 1.154 | 22 | 3.119 | 6.334 |
| AFR | 9.213 | .933 | 22 | 7.727 | 10.5 |
| TFR | 2.816 | .229 | 22 | 2.429 | 3.091 |
| lnBSS | 4.456 | .009 | 22 | 4.437 | 4.468 |
| GDP^2^ | 18754897 | 10783373 | 22 | 3143276.3 | 37145296 |
| AFR^2^ | 85.714 | 16.881 | 22 | 59.707 | 110.25 |
| DTP | 90.727 | 4.41 | 22 | 81 | 95 |
|  | **Mean** | **SD** | **N** | **Min** | **Max** |

**Country: Angola**

| U5MR | 125.018 | 44.948 | 22 | 69 | 203 |
| --- | --- | --- | --- | --- | --- |
| GDP | 2639.957 | 1464.668 | 22 | 533.586 | 5086.027 |
| IOM | 239.777 | 62.919 | 22 | 153.96 | 329.32 |
| CHE | 2.971 | .595 | 22 | 1.909 | 4.484 |
| AFR | 152.511 | 5.878 | 22 | 143.383 | 162.773 |
| TFR | 6.07 | .438 | 22 | 5.304 | 6.639 |
| lnBSS | 3.702 | .202 | 22 | 3.316 | 3.95 |
| GDP^2^ | 9017110.6 | 8623972 | 22 | 284714.25 | 25867674 |
| AFR^2^ | 23292.641 | 1797.146 | 22 | 20558.686 | 26495.049 |
| DTP | 50.273 | 6.916 | 22 | 31 | 63 |
|  | **Mean** | **SD** | **N** | **Min** | **Max** |

**Country: Benin**

| U5MR | 108.059 | 15.869 | 22 | 83.1 | 136.1 |
| --- | --- | --- | --- | --- | --- |
| GDP | 949.703 | 238.569 | 22 | 487.424 | 1318.652 |
| IOM | 424.119 | 45.063 | 22 | 361.95 | 502.44 |
| CHE | 2.919 | .234 | 22 | 2.509 | 3.304 |
| AFR | 100.383 | 12.012 | 22 | 79.804 | 119.261 |
| TFR | 5.425 | .34 | 22 | 4.707 | 5.937 |
| lnBSS | 2.596 | .258 | 22 | 2.152 | 2.964 |
| GDP^2^ | 956263.31 | 420527.91 | 22 | 237582.3 | 1738842 |
| AFR^2^ | 10214.543 | 2422.679 | 22 | 6368.678 | 14223.187 |
| DTP | 74.818 | 3.157 | 22 | 70 | 82 |
|  | **Mean** | **SD** | **N** | **Min** | **Max** |

**Country: Botswana**

| U5MR | 59.164 | 13.211 | 22 | 42 | 81.2 |
| --- | --- | --- | --- | --- | --- |
| GDP | 5834.233 | 1392.283 | 22 | 3117.251 | 7807.888 |
| IOM | 2.253 | 3.661 | 22 | .17 | 17.22 |
| CHE | 6.081 | .442 | 22 | 4.876 | 6.665 |
| AFR | 60.184 | 6.498 | 22 | 49.926 | 73.683 |
| TFR | 3.062 | .116 | 22 | 2.827 | 3.294 |
| lnBSS | 4.198 | .145 | 22 | 3.95 | 4.386 |
| GDP^2^ | 35888619 | 15197502 | 22 | 9717253 | 60963116 |
| AFR^2^ | 3662.372 | 807.991 | 22 | 2492.605 | 5429.185 |
| DTP | 95.591 | .734 | 22 | 95 | 97 |
|  | **Mean** | **SD** | **N** | **Min** | **Max** |

**Country: Burkina Faso**

| U5MR | 124.786 | 31.122 | 22 | 82.5 | 178.1 |
| --- | --- | --- | --- | --- | --- |
| GDP | 593.851 | 193.437 | 22 | 248.909 | 895.535 |
| IOM | 491.472 | 90.16 | 22 | 356.23 | 597.54 |
| CHE | 4.78 | 1.082 | 22 | 2.888 | 6.577 |
| AFR | 119.532 | 12.872 | 22 | 90.079 | 132.811 |
| TFR | 5.707 | .641 | 22 | 4.361 | 6.519 |
| lnBSS | 2.831 | .266 | 22 | 2.321 | 3.18 |
| GDP^2^ | 388376.4 | 215964.96 | 22 | 61955.453 | 801983.44 |
| AFR^2^ | 14446.08 | 2872.982 | 22 | 8114.226 | 17638.762 |
| DTP | 84.727 | 11.929 | 22 | 45 | 93 |
|  | **Mean** | **SD** | **N** | **Min** | **Max** |

**Country: Burundi**

| U5MR | 94.682 | 33.503 | 22 | 52.6 | 153.7 |
| --- | --- | --- | --- | --- | --- |
| GDP | 193.827 | 48.483 | 22 | 111.407 | 280.967 |
| IOM | 260.805 | 78.885 | 22 | 163.65 | 416.86 |
| CHE | 7.829 | 1.817 | 22 | 4.222 | 11.282 |
| AFR | 71.054 | 10.475 | 22 | 54.826 | 85.006 |
| TFR | 6.121 | .611 | 22 | 5.078 | 6.872 |
| lnBSS | 3.824 | .004 | 22 | 3.811 | 3.827 |
| GDP^2^ | 39812.644 | 18283.579 | 22 | 12411.449 | 78942.359 |
| AFR^2^ | 5153.407 | 1461.787 | 22 | 3005.89 | 7226.02 |
| DTP | 90.864 | 5.809 | 22 | 80 | 99 |
|  | **Mean** | **SD** | **N** | **Min** | **Max** |

**Country: Cabo Verde**

| U5MR | 23.818 | 6.718 | 22 | 12.7 | 36 |
| --- | --- | --- | --- | --- | --- |
| GDP | 3132.336 | 1079.653 | 22 | 1189.515 | 4380.781 |
| IOM | .459 | .686 | 22 | 0 | 3.17 |
| CHE | 4.803 | .782 | 22 | 3.925 | 7.317 |
| AFR | 70.878 | 18.674 | 22 | 38.922 | 100.642 |
| TFR | 2.35 | .567 | 22 | 1.554 | 3.545 |
| lnBSS | 4.073 | .247 | 22 | 3.606 | 4.419 |
| GDP^2^ | 10924195 | 5957426.1 | 22 | 1414945.5 | 19191244 |
| AFR^2^ | 5356.555 | 2631.236 | 22 | 1514.922 | 10128.813 |
| DTP | 94.5 | 2.972 | 22 | 90 | 99 |
|  | **Mean** | **SD** | **N** | **Min** | **Max** |

**Country: Cameroon**

| U5MR | 107.477 | 23.543 | 22 | 72.1 | 144.4 |
| --- | --- | --- | --- | --- | --- |
| GDP | 1331.012 | 298.352 | 22 | 708.566 | 1672.327 |
| IOM | 332.578 | 78.281 | 22 | 242.46 | 436.3 |
| CHE | 3.895 | .261 | 22 | 3.5 | 4.341 |
| AFR | 126.067 | 8.818 | 22 | 109.907 | 139.918 |
| TFR | 5.08 | .329 | 22 | 4.468 | 5.514 |
| lnBSS | 3.698 | .037 | 22 | 3.645 | 3.759 |
| GDP^2^ | 1856560.3 | 712476.49 | 22 | 502065.41 | 2796676.3 |
| AFR^2^ | 15967.163 | 2213.344 | 22 | 12079.549 | 19577.047 |
| DTP | 74.636 | 6.687 | 22 | 62 | 84 |
|  | **Mean** | **SD** | **N** | **Min** | **Max** |

**Country: Central African Republic**

| U5MR | 163.845 | 75.657 | 22 | 114.1 | 478.9 |
| --- | --- | --- | --- | --- | --- |
| GDP | 391.05 | 92.794 | 22 | 237.952 | 544.396 |
| IOM | 390.9 | 50.086 | 22 | 311.4 | 451.06 |
| CHE | 5.335 | 2.047 | 22 | 3.567 | 10.995 |
| AFR | 158.231 | 10.102 | 22 | 141.398 | 169.171 |
| TFR | 5.967 | .13 | 22 | 5.784 | 6.127 |
| lnBSS | 2.861 | .135 | 22 | 2.622 | 3.025 |
| GDP^2^ | 161139.14 | 71352.771 | 22 | 56621.012 | 296366.84 |
| AFR^2^ | 25134.398 | 3127.16 | 22 | 19993.395 | 28618.828 |
| DTP | 43.682 | 6.082 | 22 | 23 | 54 |
|  | **Mean** | **SD** | **N** | **Min** | **Max** |

**Country: Chad**

| U5MR | 145.873 | 23.783 | 22 | 107.6 | 183.6 |
| --- | --- | --- | --- | --- | --- |
| GDP | 863.294 | 372.321 | 22 | 163.122 | 1354.343 |
| IOM | 230.405 | 19.159 | 22 | 207.01 | 275.38 |
| CHE | 3.584 | .585 | 22 | 2.934 | 5.334 |
| AFR | 171.845 | 18.42 | 22 | 137.946 | 195.801 |
| TFR | 6.875 | .313 | 22 | 6.255 | 7.249 |
| lnBSS | 2.386 | .086 | 22 | 2.271 | 2.551 |
| GDP^2^ | 877598.89 | 562423.04 | 22 | 26608.678 | 1834246.1 |
| AFR^2^ | 29854.577 | 6122.916 | 22 | 19029.1 | 38338.031 |
| DTP | 35.636 | 10.777 | 22 | 19 | 58 |
|  | **Mean** | **SD** | **N** | **Min** | **Max** |

**Country: Comoros**

| U5MR | 56.991 | 11.292 | 22 | 42.4 | 79.6 |
| --- | --- | --- | --- | --- | --- |
| GDP | 1295.186 | 299.216 | 22 | 655.008 | 1615.717 |
| IOM | 41.994 | 26.809 | 22 | 1.98 | 76.35 |
| CHE | 5.645 | .805 | 22 | 4.601 | 7.521 |
| AFR | 74.066 | 11.925 | 22 | 57.386 | 92.011 |
| TFR | 4.673 | .385 | 22 | 3.999 | 5.227 |
| lnBSS | 3.522 | .06 | 22 | 3.374 | 3.581 |
| GDP^2^ | 1762967.5 | 694388.23 | 22 | 429035.22 | 2610542 |
| AFR^2^ | 5621.504 | 1778.52 | 22 | 3293.153 | 8466.024 |
| DTP | 77.364 | 5.661 | 22 | 68 | 89 |
|  | **Mean** | **SD** | **N** | **Min** | **Max** |

**Country: Congo Democratic Republic**

| U5MR | 114.532 | 25.519 | 22 | 78.1 | 159.2 |
| --- | --- | --- | --- | --- | --- |
| GDP | 354.059 | 135.392 | 22 | 142.678 | 558.034 |
| IOM | 385.741 | 67.153 | 22 | 295.21 | 469.27 |
| CHE | 3.593 | .695 | 22 | 1.466 | 4.511 |
| AFR | 119.64 | 5.646 | 22 | 109.234 | 125.661 |
| TFR | 6.486 | .148 | 22 | 6.156 | 6.702 |
| lnBSS | 2.963 | .125 | 22 | 2.78 | 3.167 |
| GDP^2^ | 142855.6 | 95419.774 | 22 | 20357.053 | 311402.03 |
| AFR^2^ | 14344.25 | 1329.659 | 22 | 11932.066 | 15790.687 |
| DTP | 62 | 13.009 | 22 | 30 | 73 |
|  | **Mean** | **SD** | **N** | **Min** | **Max** |

**Country: Congo Republic**

| U5MR | 69.677 | 22.579 | 22 | 43.3 | 113.7 |
| --- | --- | --- | --- | --- | --- |
| GDP | 2278.642 | 888.728 | 22 | 854.598 | 3732.327 |
| IOM | 257.525 | 51.147 | 22 | 208.16 | 352.23 |
| CHE | 2.155 | .593 | 22 | 1.505 | 4.018 |
| AFR | 123.738 | 6.387 | 22 | 112.715 | 135.039 |
| TFR | 4.633 | .158 | 22 | 4.28 | 4.851 |
| lnBSS | 2.78 | .194 | 22 | 2.478 | 3.023 |
| GDP^2^ | 5946143.5 | 4118163 | 22 | 730338.25 | 13930268 |
| AFR^2^ | 15350 | 1577.565 | 22 | 12704.671 | 18235.531 |
| DTP | 69.364 | 16.372 | 22 | 31 | 90 |
|  | **Mean** | **SD** | **N** | **Min** | **Max** |

**Country: Cote d'Ivoire**

| U5MR | 102.927 | 21.979 | 22 | 71.4 | 140.5 |
| --- | --- | --- | --- | --- | --- |
| GDP | 1594.769 | 443.746 | 22 | 924.727 | 2455.981 |
| IOM | 379.477 | 103.806 | 22 | 241.69 | 510.6 |
| CHE | 3.866 | .449 | 22 | 3.092 | 4.64 |
| AFR | 122.13 | 14.736 | 22 | 94.609 | 139.304 |
| TFR | 5.172 | .415 | 22 | 4.411 | 5.773 |
| lnBSS | 3.322 | .177 | 22 | 3.029 | 3.592 |
| GDP^2^ | 2731249 | 1447068.2 | 22 | 855120.56 | 6031844 |
| AFR^2^ | 15123.061 | 3489.662 | 22 | 8950.863 | 19405.604 |
| DTP | 74.182 | 7.449 | 22 | 61 | 87 |
|  | **Mean** | **SD** | **N** | **Min** | **Max** |

**Country: Djibouti**

| U5MR | 75.523 | 14.276 | 22 | 53.8 | 99.5 |
| --- | --- | --- | --- | --- | --- |
| GDP | 1632.642 | 848.669 | 22 | 737.612 | 3025.911 |
| IOM | 16.048 | 24.723 | 22 | 1.45 | 88.72 |
| CHE | 3.276 | .573 | 22 | 2.322 | 4.27 |
| AFR | 24.197 | 3.997 | 22 | 19.58 | 32.882 |
| TFR | 3.508 | .616 | 22 | 2.699 | 4.601 |
| lnBSS | 4.017 | .131 | 22 | 3.851 | 4.202 |
| GDP^2^ | 3353022.3 | 3075245.6 | 22 | 544071.75 | 9156139 |
| adolescent fert sq~d | 600.727 | 207.341 | 22 | 383.376 | 1081.226 |
| imm DTP | 74.727 | 12.437 | 22 | 46 | 89 |
|  | **Mean** | **SD** | **N** | **Min** | **Max** |

**Country: Egypt, Arab Rep.**

| U5MR | 29.559 | 8.385 | 22 | 18.7 | 46.6 |
| --- | --- | --- | --- | --- | --- |
| GDP | 2262.286 | 920.721 | 22 | 991.256 | 3827.354 |
| IOM | 0 | 0 | 22 | 0 | 0 |
| CHE | 4.772 | .388 | 22 | 4.153 | 5.511 |
| AFR | 53 | 4.616 | 22 | 41.901 | 57.538 |
| TFR | 3.266 | .215 | 22 | 2.75 | 3.505 |
| lnBSS | 4.549 | .021 | 22 | 4.513 | 4.58 |
| GDP^2^ | 5927132 | 4212976.8 | 22 | 982587.81 | 14648640 |
| AFR^2^ | 2829.301 | 462.635 | 22 | 1755.694 | 3310.621 |
| DTP | 96.182 | 1.79 | 22 | 93 | 99 |
|  | **Mean** | **SD** | **N** | **Min** | **Max** |

**Country: Equatorial Guinea**

| U5MR | 112.009 | 25.138 | 22 | 76.1 | 155.9 |
| --- | --- | --- | --- | --- | --- |
| GDP | 9476.275 | 5329.223 | 22 | 1487.317 | 18210.555 |
| IOM | 312.938 | 37.732 | 22 | 237.31 | 359.53 |
| CHE | 2.02 | .844 | 22 | 1.107 | 3.996 |
| AFR | 175.541 | 10.902 | 22 | 154.136 | 186.496 |
| TFR | 5.118 | .501 | 22 | 4.266 | 5.834 |
| lnBSS | 4.137 | .065 | 22 | 4.004 | 4.199 |
| GDP^2^ | 1.169e+08 | 1.086e+08 | 22 | 2212110.5 | 3.316e+08 |
| AFR^2^ | 30928.222 | 3750.654 | 22 | 23757.906 | 34780.758 |
| DTP | 50.182 | 8.797 | 22 | 35 | 67 |
|  | **Mean** | **SD** | **N** | **Min** | **Max** |

**Country: Eswatini**

| U5MR | 81.118 | 26.592 | 22 | 47.6 | 116.2 |
| --- | --- | --- | --- | --- | --- |
| GDP | 3214.965 | 896.671 | 22 | 1351.866 | 4347.996 |
| IOM | 1.367 | 1.021 | 22 | .19 | 4.74 |
| CHE | 7.26 | 1.113 | 22 | 4.61 | 8.858 |
| AFR | 87.219 | 12.915 | 22 | 70.741 | 109.963 |
| TFR | 3.39 | .366 | 22 | 2.841 | 4.03 |
| lnBSS | 4.039 | .094 | 22 | 3.886 | 4.167 |
| GDP^2^ | 11103471 | 5184318.7 | 22 | 1827540.4 | 18905066 |
| AFR^2^ | 7766.436 | 2325.052 | 22 | 5004.289 | 12091.861 |
| DTP | 88.227 | 4.8 | 22 | 77 | 98 |

|  | **Mean** | **SD** | **N** | **Min** | **Max** |
| --- | --- | --- | --- | --- | --- |

**Country: Ethiopia**

| U5MR | 86.5 | 28.358 | 22 | 50 | 140.4 |
| --- | --- | --- | --- | --- | --- |
| GDP | 431.2 | 278.265 | 22 | 109.594 | 910.948 |
| IOM | 133.048 | 59.006 | 22 | 33.61 | 219.26 |
| CHE | 4.191 | .621 | 22 | 3.208 | 5.466 |
| AFR | 107.109 | 30.269 | 22 | 72.373 | 155.407 |
| TFR | 5.313 | .879 | 22 | 4.177 | 6.654 |
| lnBSS | 1.738 | .363 | 22 | 1.027 | 2.225 |
| GDP^2^ | 259845.24 | 276170.73 | 22 | 12010.804 | 829826.06 |
| AFR^2^ | 12346.991 | 6761.096 | 22 | 5237.851 | 24151.336 |
| DTP | 54.636 | 13.088 | 22 | 30 | 71 |
|  | **Mean** | **SD** | **N** | **Min** | **Max** |

**Country: Gabon**

| U5MR | 53.318 | 12.326 | 22 | 35.2 | 73.7 |
| --- | --- | --- | --- | --- | --- |
| GDP | 7141.356 | 1869.862 | 22 | 3835.757 | 10219.114 |
| IOM | 225.374 | 53.154 | 22 | 128.69 | 329.75 |
| CHE | 2.959 | .371 | 22 | 2.421 | 3.835 |
| AFR | 112.118 | 11.433 | 22 | 94.925 | 132.158 |
| TFR | 4.095 | .193 | 22 | 3.776 | 4.469 |
| lnBSS | 3.769 | .13 | 22 | 3.526 | 3.909 |
| GDP^2^ | 54336418 | 25893709 | 22 | 14713033 | 1.044e+08 |
| AFR^2^ | 12695.113 | 2554.914 | 22 | 9010.756 | 17465.736 |
| DTP | 65.227 | 14.893 | 22 | 45 | 82 |
|  | **Mean** | **SD** | **N** | **Min** | **Max** |

**Country: Gambia, The**

| U5MR | 74.782 | 20.484 | 22 | 47.2 | 113.1 |
| --- | --- | --- | --- | --- | --- |
| GDP | 642.773 | 134.094 | 22 | 308.719 | 859.128 |
| IOM | 203.635 | 88.036 | 22 | 44.76 | 309.72 |
| CHE | 4.713 | 1.858 | 22 | 3.052 | 10.785 |
| AFR | 88.067 | 17.173 | 22 | 60.503 | 116.941 |
| TFR | 5.25 | .554 | 22 | 4.165 | 5.736 |
| lnBSS | 3.854 | .025 | 22 | 3.836 | 3.933 |
| GDP^2^ | 430320.51 | 158994.73 | 22 | 95307.5 | 738100.38 |
| AFR^2^ | 8037.377 | 2980.778 | 22 | 3660.613 | 13675.197 |
| DTP | 91.955 | 5.525 | 22 | 80 | 98 |
|  | **Mean** | **SD** | **N** | **Min** | **Max** |

**Country: Ghana**

| U5MR | 66.727 | 18.713 | 22 | 40.2 | 100.2 |
| --- | --- | --- | --- | --- | --- |
| GDP | 1339.763 | 758.202 | 22 | 253.747 | 2445.187 |
| IOM | 314.806 | 67.469 | 22 | 216.42 | 432.01 |
| CHE | 3.49 | .952 | 22 | 1.789 | 4.734 |
| AFR | 72.251 | 5.959 | 22 | 59.76 | 84.342 |
| TFR | 4.157 | .398 | 22 | 3.492 | 4.827 |
| lnBSS | 2.668 | .47 | 22 | 1.832 | 3.31 |
| GDP^2^ | 2343704.6 | 1994717 | 22 | 64387.508 | 5978937.5 |
| AFR^2^ | 5254.115 | 859.933 | 22 | 3571.258 | 7113.573 |
| DTP | 90.227 | 6.683 | 22 | 78 | 99 |
|  | **Mean** | **SD** | **N** | **Min** | **Max** |

**Country: Guinea**

| U5MR | 125.414 | 18.164 | 22 | 100.5 | 165.4 |
| --- | --- | --- | --- | --- | --- |
| GDP | 710.893 | 206.216 | 22 | 446.981 | 1244.966 |
| IOM | 377.17 | 36.032 | 22 | 326.59 | 430.03 |
| CHE | 4.184 | .746 | 22 | 3.266 | 5.809 |
| AFR | 136.537 | 8.311 | 22 | 122.721 | 151.173 |
| TFR | 5.286 | .482 | 22 | 4.399 | 5.918 |
| lnBSS | 2.87 | .366 | 22 | 2.215 | 3.402 |
| GDP^2^ | 545961.1 | 336436.54 | 22 | 199791.61 | 1549939.1 |
| AFR^2^ | 18708.188 | 2258.552 | 22 | 15060.443 | 22853.275 |
| DTP | 53.136 | 6.614 | 22 | 44 | 64 |
|  | **Mean** | **SD** | **N** | **Min** | **Max** |

**Country: Guinea-Bissau**

| U5MR | 116.977 | 32.307 | 22 | 74.1 | 173.8 |
| --- | --- | --- | --- | --- | --- |
| GDP | 602.065 | 172.552 | 22 | 316.945 | 912.484 |
| IOM | 133.104 | 87.983 | 22 | 65.1 | 409.27 |
| CHE | 6.132 | 1.816 | 22 | 3.533 | 9.969 |
| AFR | 106.897 | 13.439 | 22 | 84.568 | 124.284 |
| TFR | 4.989 | .564 | 22 | 3.998 | 5.786 |
| lnBSS | 2.525 | .605 | 22 | 1.426 | 3.323 |
| GDP^2^ | 390902.74 | 207902.47 | 22 | 100454.45 | 832627.25 |
| AFR^2^ | 11599.262 | 2808.901 | 22 | 7151.747 | 15446.513 |
| DTP | 74.227 | 11.682 | 22 | 49 | 87 |
|  | **Mean** | **SD** | **N** | **Min** | **Max** |

**Country: Kenya**

| U5MR | 59.764 | 17.054 | 22 | 41.9 | 96.3 |
| --- | --- | --- | --- | --- | --- |
| GDP | 1135.671 | 563.243 | 22 | 402.936 | 2061.356 |
| IOM | 106.998 | 66.61 | 22 | 58.68 | 245.45 |
| CHE | 4.58 | .547 | 22 | 3.624 | 5.388 |
| AFR | 97.19 | 22.602 | 22 | 59.38 | 123.253 |
| TFR | 4.269 | .621 | 22 | 3.312 | 5.136 |
| lnBSS | 3.462 | .078 | 22 | 3.334 | 3.587 |
| GDP^2^ | 1592571.9 | 1336995.7 | 22 | 162357.73 | 4249189.5 |
| AFR^2^ | 9933.625 | 4133.82 | 22 | 3525.984 | 15191.302 |
| DTP | 86.091 | 6.976 | 22 | 73 | 96 |

|  | **Mean** | **SD** | **N** | **Min** | **Max** |
| --- | --- | --- | --- | --- | --- |

**Country: Liberia**

| U5MR | 114.305 | 33.695 | 22 | 77.4 | 192.4 |
| --- | --- | --- | --- | --- | --- |
| GDP | 507.387 | 178.559 | 22 | 239.78 | 714.613 |
| IOM | 354.662 | 58.656 | 22 | 225.61 | 434.88 |
| CHE | 7.832 | 3.441 | 22 | 2.279 | 13.838 |
| AFR | 141.895 | 6.524 | 22 | 129.044 | 151.496 |
| TFR | 4.995 | .572 | 22 | 4.089 | 5.875 |
| lnBSS | 2.784 | .19 | 22 | 2.489 | 3.088 |
| GDP^2^ | 287876.19 | 176525.45 | 22 | 57494.234 | 510671.84 |
| AFR^2^ | 20174.929 | 1831.175 | 22 | 16652.354 | 22951.037 |
| DTP | 63.773 | 15.672 | 22 | 31 | 81 |
|  | **Mean** | **SD** | **N** | **Min** | **Max** |

**Country: Madagascar**

| U5MR | 76.168 | 12.705 | 22 | 65.7 | 105 |
| --- | --- | --- | --- | --- | --- |
| GDP | 424.276 | 85.998 | 22 | 272.201 | 515.302 |
| IOM | 64.411 | 34.222 | 22 | 29.9 | 166.33 |
| CHE | 4.44 | .617 | 22 | 3.101 | 5.332 |
| AFR | 140.714 | 5.112 | 22 | 133.195 | 149.931 |
| TFR | 4.794 | .496 | 22 | 4.104 | 5.665 |
| lnBSS | 2.115 | .397 | 22 | 1.341 | 2.655 |
| GDP^2^ | 187069.77 | 68664.257 | 22 | 74093.25 | 265535.72 |
| AFR^2^ | 19825.238 | 1444.656 | 22 | 17740.908 | 22479.305 |
| DTP | 70.182 | 8.325 | 22 | 57 | 85 |
|  | **Mean** | **SD** | **N** | **Min** | **Max** |

**Country: Malawi**

| U5MR | 87.018 | 39.326 | 22 | 41.5 | 173.3 |
| --- | --- | --- | --- | --- | --- |
| GDP | 500.784 | 130.58 | 22 | 215.475 | 763.061 |
| IOM | 322.734 | 85.597 | 22 | 201.69 | 463.81 |
| CHE | 5.579 | 1.812 | 22 | 2.06 | 8.224 |
| AFR | 140.079 | 14.474 | 22 | 116.517 | 158.396 |
| TFR | 5.043 | .803 | 22 | 3.791 | 6.002 |
| lnBSS | 3.064 | .591 | 22 | 2.236 | 3.852 |
| GDP^2^ | 267060.95 | 126380.58 | 22 | 46429.27 | 582262.06 |
| AFR^2^ | 19822.141 | 4002.502 | 22 | 13576.211 | 25089.293 |
| DTP | 89.318 | 7.656 | 22 | 64 | 99 |
|  | **Mean** | **SD** | **N** | **Min** | **Max** |

**Country: Mali**

| U5MR | 135.859 | 27.676 | 22 | 96.9 | 187.6 |
| --- | --- | --- | --- | --- | --- |
| GDP | 746.695 | 246.532 | 22 | 256.2 | 1027.273 |
| IOM | 388.546 | 28.973 | 22 | 325.76 | 441 |
| CHE | 4.471 | .702 | 22 | 3.466 | 5.466 |
| AFR | 163.61 | 10.647 | 22 | 141.933 | 177.855 |
| TFR | 6.477 | .324 | 22 | 5.779 | 6.886 |
| lnBSS | 3.378 | .35 | 22 | 2.735 | 3.879 |
| GDP^2^ | 615568.15 | 319530.47 | 22 | 65638.211 | 1055290.8 |
| AFR^2^ | 26876.344 | 3396.306 | 22 | 20144.977 | 31632.4 |
| DTP | 69.091 | 9.185 | 22 | 43 | 78 |
|  | **Mean** | **SD** | **N** | **Min** | **Max** |

**Country: Mauritania**

| U5MR | 60.805 | 17.387 | 22 | 40.3 | 98.2 |
| --- | --- | --- | --- | --- | --- |
| GDP | 1426.35 | 456.129 | 22 | 648.88 | 1947.789 |
| IOM | 63.786 | 24.106 | 22 | 24.34 | 94.89 |
| CHE | 3.224 | .451 | 22 | 2.474 | 4.033 |
| AFR | 97.324 | 4.472 | 22 | 89.946 | 102.904 |
| TFR | 5.272 | .183 | 22 | 4.848 | 5.456 |
| lnBSS | 3.48 | .341 | 22 | 2.89 | 3.985 |
| GDP^2^ | 2233071.2 | 1171940.3 | 22 | 421044.78 | 3793882.8 |
| AFR^2^ | 9490.996 | 863.934 | 22 | 8090.283 | 10589.233 |
| DTP | 74.136 | 7.717 | 22 | 51 | 89 |
|  | **Mean** | **SD** | **N** | **Min** | **Max** |

**Country: Morocco**

| U5MR | 32.114 | 11.042 | 22 | 17.9 | 52.2 |
| --- | --- | --- | --- | --- | --- |
| GDP | 2839.227 | 705.562 | 22 | 1499.107 | 3785.936 |
| IOM | 0 | 0 | 22 | 0 | 0 |
| CHE | 4.856 | .579 | 22 | 3.693 | 5.591 |
| AFR | 30.761 | 2.573 | 22 | 26.205 | 35.709 |
| TFR | 2.524 | .137 | 22 | 2.288 | 2.774 |
| lnBSS | 4.356 | .08 | 22 | 4.215 | 4.47 |
| GDP^2^ | 8536397.7 | 3640897.6 | 22 | 2247323 | 14333314 |
| AFR^2^ | 952.567 | 157.269 | 22 | 686.702 | 1275.133 |
| DTP | 97.682 | 2.191 | 22 | 91 | 99 |
|  | **Mean** | **SD** | **N** | **Min** | **Max** |

**Country: Mozambique**

| U5MR | 100.477 | 30.877 | 22 | 65.8 | 163 |
| --- | --- | --- | --- | --- | --- |
| GDP | 497.408 | 118.653 | 22 | 304.792 | 697.143 |
| IOM | 391.232 | 64.424 | 22 | 303.12 | 504.4 |
| CHE | 5.347 | 1.947 | 22 | 2.143 | 8.87 |
| AFR | 163.037 | 4.827 | 22 | 155.861 | 170.375 |
| TFR | 5.415 | .271 | 22 | 4.905 | 5.83 |
| lnBSS | 3.042 | .386 | 22 | 2.325 | 3.586 |
| GDP^2^ | 260853.3 | 119764.27 | 22 | 92898.25 | 486007.91 |
| AFR^2^ | 26603.228 | 1574.563 | 22 | 24292.65 | 29027.641 |
| DTP | 78.136 | 7.133 | 22 | 56 | 85 |
|  | Mean | SD | N | Min | Max |

**Country: Namibia**

| U5MR | 59.032 | 11.078 | 22 | 43.8 | 77.9 |
| --- | --- | --- | --- | --- | --- |
| GDP | 4226.034 | 1229.103 | 22 | 1772.076 | 5942.647 |
| IOM | 24.205 | 22.802 | 22 | 1.55 | 69.81 |
| CHE | 9.463 | .674 | 22 | 8.286 | 10.716 |
| AFR | 75.826 | 3.469 | 22 | 68.37 | 83.882 |
| TFR | 3.598 | .147 | 22 | 3.303 | 3.991 |
| lnBSS | 3.466 | .074 | 22 | 3.334 | 3.571 |
| GDP^2^ | 19301390 | 9538838 | 22 | 3140252.3 | 35315052 |
| AFR^2^ | 5761.097 | 527.03 | 22 | 4674.457 | 7036.19 |
| DTP | 85.091 | 4.75 | 22 | 77 | 94 |
|  | **Mean** | **SD** | **N** | **Min** | **Max** |

**Country: Niger**

| U5MR | 150.905 | 34.185 | 22 | 119.1 | 227.8 |
| --- | --- | --- | --- | --- | --- |
| GDP | 437.24 | 135.165 | 22 | 194.772 | 608.722 |
| IOM | 357.733 | 43.813 | 22 | 298.92 | 427.97 |
| CHE | 5.139 | .514 | 22 | 4.307 | 6.17 |
| AFR | 186.63 | 18.349 | 22 | 148.248 | 206.357 |
| TFR | 7.369 | .516 | 22 | 6.2 | 7.829 |
| lnBSS | 2.322 | .317 | 22 | 1.708 | 2.765 |
| GDP^2^ | 208618.14 | 108690.95 | 22 | 37936.117 | 370543 |
| AFR^2^ | 35152.134 | 6593.952 | 22 | 21977.469 | 42583.211 |
| DTP | 64.818 | 17.864 | 22 | 34 | 85 |
|  | **Mean** | **SD** | **N** | **Min** | **Max** |

**Country: Nigeria**

| U5MR | 139.886 | 20.691 | 22 | 111.3 | 182.3 |
| --- | --- | --- | --- | --- | --- |
| GDP | 1830.029 | 745.398 | 22 | 547.318 | 3088.721 |
| IOM | 344.875 | 49.194 | 22 | 283.81 | 402.01 |
| CHE | 3.624 | .589 | 22 | 2.491 | 5.054 |
| AFR | 119.716 | 15.999 | 22 | 88.457 | 135.632 |
| TFR | 5.678 | .499 | 22 | 4.636 | 6.128 |
| lnBSS | 3.562 | .156 | 22 | 3.332 | 3.817 |
| GDP^2^ | 3879370.3 | 2586606.2 | 22 | 299557.31 | 9540199 |
| AFR^2^ | 14576.147 | 3594.215 | 22 | 7824.641 | 18396.039 |
| DTP | 45.818 | 12.768 | 22 | 25 | 66 |
|  | **Mean** | **SD** | **N** | **Min** | **Max** |

**Country: Rwanda**

| U5MR | 80.423 | 43.978 | 22 | 41.6 | 184.9 |
| --- | --- | --- | --- | --- | --- |
| GDP | 556.124 | 219.011 | 22 | 234.043 | 829.545 |
| IOM | 205.801 | 161.426 | 22 | 36.94 | 711.44 |
| CHE | 6.754 | 1.49 | 22 | 3.128 | 8.102 |
| AFR | 39.077 | 4.384 | 22 | 31.158 | 47.24 |
| TFR | 4.73 | .754 | 22 | 3.841 | 5.967 |
| lnBSS | 4.077 | .152 | 22 | 3.805 | 4.302 |
| GDP^2^ | 355059.75 | 228101.17 | 22 | 54776.133 | 688144.63 |
| AFR^2^ | 1545.33 | 335.309 | 22 | 970.821 | 2231.618 |
| DTP | 94.591 | 5.342 | 22 | 77 | 99 |
|  | **Mean** | **SD** | **N** | **Min** | **Max** |

**Country: Sao Tome and Principe**

| U5MR | 39.777 | 21.098 | 22 | 15.3 | 82.1 |
| --- | --- | --- | --- | --- | --- |
| GDP | 1214.956 | 522.609 | 22 | 518.881 | 2362.588 |
| IOM | 86.961 | 113.297 | 22 | 8.89 | 337.42 |
| CHE | 7.745 | 1.786 | 22 | 5.481 | 11.465 |
| AFR | 114.732 | 17.05 | 22 | 87.903 | 136.144 |
| TFR | 4.599 | .454 | 22 | 3.759 | 5.157 |
| lnBSS | 3.493 | .26 | 22 | 3.026 | 3.866 |
| GDP^2^ | 1736824.4 | 1454633.8 | 22 | 269236.97 | 5581823.5 |
| AFR^2^ | 13440.87 | 3806.154 | 22 | 7726.938 | 18535.189 |
| DTP | 95.273 | 3.425 | 22 | 82 | 99 |
|  | **Mean** | **SD** | **N** | **Min** | **Max** |

**Country: Senegal**

| U5MR | 71.436 | 26.455 | 22 | 42.5 | 129.3 |
| --- | --- | --- | --- | --- | --- |
| GDP | 1185.438 | 293.256 | 22 | 603.233 | 1598.107 |
| IOM | 88.007 | 56.163 | 22 | 32.88 | 233.09 |
| CHE | 4.13 | .421 | 22 | 3.415 | 5.151 |
| AFR | 79.875 | 10.088 | 22 | 62.176 | 94.241 |
| TFR | 4.879 | .448 | 22 | 3.936 | 5.498 |
| lnBSS | 3.861 | .147 | 22 | 3.607 | 4.08 |
| GDP^2^ | 1487354 | 632594.03 | 22 | 363890.25 | 2553945.3 |
| AFR^2^ | 6477.101 | 1573.381 | 22 | 3865.855 | 8881.366 |
| DTP | 84.818 | 13.208 | 22 | 52 | 96 |
|  | **Mean** | **SD** | **N** | **Min** | **Max** |

**Country: Sierra Leone**

| U5MR | 159.177 | 39.501 | 22 | 101.4 | 223.7 |
| --- | --- | --- | --- | --- | --- |
| GDP | 699.312 | 255.076 | 22 | 143.744 | 1117.759 |
| IOM | 408.819 | 42.183 | 22 | 322.22 | 459.16 |
| CHE | 10.397 | 3.408 | 22 | 8.29 | 19.69 |
| AFR | 130.133 | 20.318 | 22 | 98.06 | 160.004 |
| TFR | 5.192 | .773 | 22 | 3.978 | 6.355 |
| lnBSS | 2.563 | .343 | 22 | 2.041 | 3.089 |
| GDP^2^ | 551143.28 | 345350.42 | 22 | 20662.264 | 1249384.4 |
| AFR^2^ | 17328.674 | 5249.282 | 22 | 9615.764 | 25601.279 |
| DTP | 77.227 | 16.702 | 22 | 38 | 95 |
|  | **Mean** | **SD** | **N** | **Min** | **Max** |

**Country: South Africa**

| U5MR | 54.545 | 18.63 | 22 | 34.1 | 81 |
| --- | --- | --- | --- | --- | --- |
| GDP | 6010.959 | 1600.688 | 22 | 2688.237 | 8646.056 |
| IOM | 1.991 | 1.278 | 22 | .48 | 5.57 |
| CHE | 7.655 | .64 | 22 | 6.678 | 9.013 |
| AFR | 57.415 | 6.573 | 22 | 46.465 | 70.224 |
| TFR | 2.401 | .114 | 22 | 2.248 | 2.679 |
| lnBSS | 4.223 | .079 | 22 | 4.087 | 4.342 |
| GDP^2^ | 38577361 | 17753211 | 22 | 7226617 | 74754280 |
| AFR^2^ | 3337.679 | 766.842 | 22 | 2158.996 | 4931.41 |
| DTP | 79.545 | 5.845 | 22 | 69 | 86 |
|  | **Mean** | **SD** | **N** | **Min** | **Max** |

**Country: Sudan**

| U5MR | 76.368 | 16.545 | 22 | 53.6 | 103.4 |
| --- | --- | --- | --- | --- | --- |
| GDP | 949.45 | 344.86 | 22 | 362.117 | 1527.582 |
| IOM | 50.961 | 16.447 | 22 | 31.01 | 87.71 |
| CHE | 4.821 | 1.163 | 22 | 2.831 | 6.965 |
| AFR | 88.275 | 12.652 | 22 | 69.14 | 106.954 |
| TFR | 5.088 | .347 | 22 | 4.457 | 5.596 |
| lnBSS | 3.337 | .234 | 22 | 3.025 | 3.608 |
| GDP^2^ | 1014978.2 | 656085.78 | 22 | 131128.64 | 2333508 |
| AFR^2^ | 7945.313 | 2257.201 | 22 | 4780.34 | 11439.158 |
| DTP | 83.682 | 11.201 | 22 | 60 | 95 |
|  | **Mean** | **SD** | **N** | **Min** | **Max** |

**Country: Tanzania**

| U5MR | 73.9 | 26.107 | 22 | 42.2 | 128.4 |
| --- | --- | --- | --- | --- | --- |
| GDP | 752.175 | 267.579 | 22 | 396.639 | 1159.857 |
| IOM | 184.949 | 74.708 | 22 | 110.65 | 337.7 |
| CHE | 4.79 | 1.148 | 22 | 3.052 | 7.598 |
| AFR | 123.63 | 6.336 | 22 | 115.452 | 132.824 |
| TFR | 5.238 | .292 | 22 | 4.734 | 5.67 |
| lnBSS | 2.852 | .464 | 22 | 1.865 | 3.416 |
| GDP^2^ | 634110.95 | 401945.27 | 22 | 157322.28 | 1345267.3 |
| AFR^2^ | 15322.571 | 1579.617 | 22 | 13329.164 | 17642.215 |
| DTP | 89.773 | 4.275 | 22 | 79 | 97 |
|  | **Mean** | **SD** | **N** | **Min** | **Max** |

**Country: Togo**

| U5MR | 88.441 | 17.652 | 22 | 62.2 | 119.6 |
| --- | --- | --- | --- | --- | --- |
| GDP | 691.897 | 158.264 | 22 | 396.693 | 939.613 |
| IOM | 359.65 | 80.603 | 22 | 236.78 | 466.89 |
| CHE | 4.471 | 1.356 | 22 | 2.717 | 6.556 |
| AFR | 86.129 | 4.651 | 22 | 79.696 | 96.414 |
| TFR | 4.853 | .276 | 22 | 4.324 | 5.165 |
| lnBSS | 2.615 | .225 | 22 | 2.262 | 2.948 |
| GDP^2^ | 502629.83 | 208317.08 | 22 | 157365.06 | 882873.31 |
| AFR^2^ | 7438.837 | 824.683 | 22 | 6351.453 | 9295.659 |
| DTP | 78.182 | 9.328 | 22 | 50 | 85 |
|  | **Mean** | **SD** | **N** | **Min** | **Max** |

**Country: Uganda**

| U5MR | 81.5 | 33.149 | 22.000 | 41.8 | 145.6 |
| --- | --- | --- | --- | --- | --- |
| GDP | 614.621 | 262.49 | 22 | 236.021 | 896.055 |
| IOM | 374.783 | 87.58 | 22 | 253.15 | 508.34 |
| CHE | 5.309 | .851 | 22 | 3.794 | 6.809 |
| AFR | 135.96 | 17.006 | 22 | 110.964 | 169.238 |
| TFR | 5.812 | .768 | 22 | 4.51 | 6.788 |
| lnBSS | 2.922 | .074 | 22 | 2.796 | 3.036 |
| GDP^2^ | 443528.64 | 295412.81 | 22 | 55706 | 802914.19 |
| AFR^2^ | 18761.266 | 4726.948 | 22 | 12313.01 | 28641.5 |
| DTP | 76.909 | 14.101 | 22 | 52 | 94 |
|  | **Mean** | **SD** | **N** | **Min** | **Max** |

**Country: Zambia**

| U5MR | 84.75 | 31.443 | 22 | 49.2 | 152.5 |
| --- | --- | --- | --- | --- | --- |
| GDP | 1121.47 | 464.906 | 22 | 359.43 | 1820.719 |
| IOM | 232.343 | 70.94 | 22 | 165.49 | 379.02 |
| CHE | 5.046 | .927 | 22 | 3.829 | 6.753 |
| AFR | 134.066 | 9.961 | 22 | 118.744 | 155.69 |
| TFR | 5.185 | .553 | 22 | 4.246 | 5.921 |
| lnBSS | 3.338 | .189 | 22 | 2.993 | 3.59 |
| GDP^2^ | 1464008.6 | 984149 | 22 | 129189.56 | 3315016 |
| AFR^2^ | 18068.505 | 2725.014 | 22 | 14100.138 | 24239.377 |
| DTP | 85.591 | 4.973 | 22 | 78 | 95 |

Source: Authors’ compilation

**_Note:_** _GDP: Gross Domestic Product per capita (lagged); DTP: Diphtheria-Tetanus-Pertussis immunisation; IOM: Incidence of Malaria; CHE: Current Health Expenditure (lagged); AFR: Adolescent Fertility Rate; TFR: Total Fertility Rate; ln BSS: Natural log of Basic Sanitation Services access. GDP_^2^ _and AFR_^2^ _represent quadratic terms used to model non-linear biological and economic risks. U5MR is measured per 1,000 live births; GDP per capita is in current US$, DTP is measured in Share of one-year-olds vaccinated against diphtheria, tetanus and pertussis, IOM is measured in number of new cases of malaria in a year per 1,000 population at risk, CHE is measured in Current health expenditure % of GDP, AFR is measured in number of births per 1,000 women ages 15-19, TFR is measured in total births per woman, BSS is measured in people using at least basic sanitation services (% of population)_
