# Supplementary material for: Determinants of under-five mortality in Africa: evidence from a two-decade panel analysis for public health policy
Source: Arch Public Health. 2026 Jun 17;84:193. doi: 10.1186/s13690-026-01994-0 (PMC13520361; doi:10.1186/s13690-026-01994-0)
Supplement: Supplementary file 5 [file 13690_2026_1994_MOESM6_ESM.docx]

**Appendix 6: Assessment of stationarity properties for under-five mortality and its determinants across 45 African nations using Levin-Lin-Chu unit root tests (2000–2021)**

**Levin-Lin-Chu Unit Root Test Results**

Levinâ€“Linâ€“Chu unit-root test for mortality_u5

H0: Panels contain unit roots	Number of panels =	45

Ha: Panels are stationary	Number of periods =	22

AR parameter: Common	Asymptotics: N/T -> 0

Panel means: Included

Time trend: Not included

ADF regressions: 1 lag

LR variance: Bartlett kernel, 8.00 lags average (chosen by LLC)

Statistic	p-value

Unadjusted t	-16.8060

Adjusted t*	-14.6311	0.0000

Levinâ€“Linâ€“Chu unit-root test for gdp_per_capita

H0: Panels contain unit roots	Number of panels =	45

Ha: Panels are stationary	Number of periods =	22

AR parameter: Common	Asymptotics: N/T -> 0

Panel means: Included

Time trend: Not included

ADF regressions: 1 lag

LR variance: Bartlett kernel, 8.00 lags average (chosen by LLC)

Statistic	p-value

Unadjusted t	-7.2091

Adjusted t*	-2.7761	0.0028

Levinâ€“Linâ€“Chu unit-root test for imm_dtp

H0: Panels contain unit roots	Number of panels =	45

Ha: Panels are stationary	Number of periods =	22

AR parameter: Common	Asymptotics: N/T -> 0

Panel means: Included

Time trend: Not included

ADF regressions: 1 lag

LR variance: Bartlett kernel, 8.00 lags average (chosen by LLC)

Statistic	p-value

Unadjusted t	-14.6549

Adjusted t*	-7.1442	0.0000

Levinâ€“Linâ€“Chu unit-root test for malaria_inc

H0: Panels contain unit roots	Number of panels =	45

Ha: Panels are stationary	Number of periods =	22

AR parameter: Common	Asymptotics: N/T -> 0

Panel means: Included

Time trend: Not included

ADF regressions: 1 lag

LR variance: Bartlett kernel, 8.00 lags average (chosen by LLC)

Statistic	p-value

Unadjusted t	-14.7873

Adjusted t*	-9.6652	0.0000

Levinâ€“Linâ€“Chu unit-root test for health_expenditure

H0: Panels contain unit roots	Number of panels =	45

Ha: Panels are stationary	Number of periods =	22

AR parameter: Common	Asymptotics: N/T -> 0

Panel means: Included

Time trend: Not included

ADF regressions: 1 lag

LR variance: Bartlett kernel, 8.00 lags average (chosen by LLC)

Statistic	p-value

Unadjusted t	-10.1856

Adjusted t*	-2.4674	0.0068

Levinâ€“Linâ€“Chu unit-root test for adolescent_fert

H0: Panels contain unit roots	Number of panels =	45

Ha: Panels are stationary	Number of periods =	22

AR parameter: Common	Asymptotics: N/T -> 0

Panel means: Included

Time trend: Not included

ADF regressions: 1 lag

LR variance: Bartlett kernel, 8.00 lags average (chosen by LLC)

Statistic	p-value

Unadjusted t	-4.3203

Adjusted t*	-0.7280	0.2333

Levinâ€“Linâ€“Chu unit-root test for adolescent_fert_squared

H0: Panels contain unit roots	Number of panels =	45

Ha: Panels are stationary	Number of periods =	22

AR parameter: Common	Asymptotics: N/T -> 0

Panel means: Included

Time trend: Not included

ADF regressions: 1 lag

LR variance: Bartlett kernel, 8.00 lags average (chosen by LLC)

Statistic	p-value

Unadjusted t	-6.2236

Adjusted t*	-2.5831	0.0049

Levinâ€“Linâ€“Chu unit-root test for fert_rate

H0: Panels contain unit roots	Number of panels =	45

Ha: Panels are stationary	Number of periods =	22

AR parameter: Common	Asymptotics: N/T -> 0

Panel means: Included

Time trend: Not included

ADF regressions: 1 lag

LR variance: Bartlett kernel, 8.00 lags average (chosen by LLC)

Statistic	p-value

Unadjusted t	-6.7086

Adjusted t*	-5.1244	0.0000

Levinâ€“Linâ€“Chu unit-root test for basic_sanitation

H0: Panels contain unit roots	Number of panels =	45

Ha: Panels are stationary	Number of periods =	22

AR parameter: Common	Asymptotics: N/T -> 0

Panel means: Included

Time trend: Not included

ADF regressions: 1 lag

LR variance: Bartlett kernel, 8.00 lags average (chosen by LLC)

Statistic	p-value

Unadjusted t	9.5356

Adjusted t*	10.7955	1.0000

Levinâ€“Linâ€“Chu unit-root test for ln_basic_sanitation

H0: Panels contain unit roots	Number of panels =	45

Ha: Panels are stationary	Number of periods =	22

AR parameter: Common	Asymptotics: N/T -> 0

Panel means: Included

Time trend: Not included

ADF regressions: 1 lag

LR variance: Bartlett kernel, 8.00 lags average (chosen by LLC)

Statistic	p-value

Unadjusted t	-27.5971

Adjusted t*	-28.2400	0.0000

Levinâ€“Linâ€“Chu unit-root test for corruption_rank

H0: Panels contain unit roots	Number of panels =	45

Ha: Panels are stationary	Number of periods =	22

AR parameter: Common	Asymptotics: N/T -> 0

Panel means: Included

Time trend: Not included

ADF regressions: 1 lag

LR variance: Bartlett kernel, 8.00 lags average (chosen by LLC)

Statistic	p-value

Unadjusted t	-11.4520

Adjusted t*	-2.7839	0.0027

Levinâ€“Linâ€“Chu unit-root test for imm_measles

H0: Panels contain unit roots	Number of panels =	45

Ha: Panels are stationary	Number of periods =	22

AR parameter: Common	Asymptotics: N/T -> 0

Panel means: Included

Time trend: Not included

ADF regressions: 1 lag

LR variance: Bartlett kernel, 8.00 lags average (chosen by LLC)

Statistic	p-value

Unadjusted t	-14.1330

Adjusted t*	-6.2152	0.0000

Levinâ€“Linâ€“Chu unit-root test for clean_fuel

H0: Panels contain unit roots	Number of panels =	45

Ha: Panels are stationary	Number of periods =	22

AR parameter: Common	Asymptotics: N/T -> 0

Panel means: Included

Time trend: Not included

ADF regressions: 1 lag

LR variance: Bartlett kernel, 8.00 lags average (chosen by LLC)

Statistic	p-value

Unadjusted t	-2.8255

Adjusted t*	2.4059	0.9919

Levinâ€“Linâ€“Chu unit-root test for D_clean_fuel

H0: Panels contain unit roots	Number of panels =	45

Ha: Panels are stationary	Number of periods =	21

AR parameter: Common	Asymptotics: N/T -> 0

Panel means: Included

Time trend: Not included

ADF regressions: 1 lag

LR variance: Bartlett kernel, 8.00 lags average (chosen by LLC)

Statistic	p-value

Unadjusted t	-5.5226

Adjusted t*	-0.3819	0.3513

Levinâ€“Linâ€“Chu unit-root test for D2_clean_fuel

H0: Panels contain unit roots	Number of panels =	45

Ha: Panels are stationary	Number of periods =	20

AR parameter: Common	Asymptotics: N/T -> 0

Panel means: Included

Time trend: Not included

ADF regressions: 1 lag

LR variance: Bartlett kernel, 8.00 lags average (chosen by LLC)

Statistic	p-value

Unadjusted t	-26.9494

Adjusted t*	-13.2744	0.0000

Levinâ€“Linâ€“Chu unit-root test for air_pollution

H0: Panels contain unit roots	Number of panels =	45

Ha: Panels are stationary	Number of periods =	22

AR parameter: Common	Asymptotics: N/T -> 0

Panel means: Included

Time trend: Not included

ADF regressions: 1 lag

LR variance: Bartlett kernel, 8.00 lags average (chosen by LLC)

Statistic	p-value

Unadjusted t	-15.5186

Adjusted t*	-6.3688	0.0000

Source: Authors’ compilation

**Note:** mortality_u5: Under-five mortality rate (per 1,000 live births); gdp_per_capita: Gross Domestic Product per capita (current US$); imm_dtp: Diphtheria-Tetanus-Pertussis immunization coverage (% of children ages 12–23 months); malaria_inc: Incidence of malaria (new cases per 1,000 population at risk); health_expenditure: Current health expenditure (% of GDP); adolescent_fert: Adolescent fertility rate (births per 1,000 women ages 15–19); fert_rate: Total fertility rate (births per woman); basic_sanitation: Percentage of population using at least basic sanitation services; ln_basic_sanitation: Natural log of basic sanitation access; corruption_rank: Control of Corruption percentile rank (0–100); imm_measles: Measles immunization coverage (%); clean_fuel: Access to clean fuels and technologies for cooking (% of population); air_pollution: PM2.5 mean annual exposure (micrograms per cubic meter). LLC: Levin-Lin-Chu Unit Root Test. H0: Null hypothesis (Panels contain unit roots); Ha: Alternative hypothesis (Panels are stationary). AR parameter: Autoregressive parameter; Asymptotics: Large-sample properties; ADF: Augmented Dickey-Fuller regressions (1 lag); LR variance: Long-run variance estimated using the Bartlett kernel (average lags chosen by LLC).
