# Supplementary material for: Determinants of under-five mortality in Africa: evidence from a two-decade panel analysis for public health policy
Source: Arch Public Health. 2026 Jun 17;84:193. doi: 10.1186/s13690-026-01994-0 (PMC13520361; doi:10.1186/s13690-026-01994-0)
Supplement: Supplementary file 6 [file 13690_2026_1994_MOESM7_ESM.docx]

**Appendix 7: Diagnostics for multicollinearity among determinants of under-five mortality across 45 African nations: Pairwise correlation matrix and Variance Inflation Factor results (2000–2021)**

**Table 7.A: Pairwise correlation matrix identifying associations between under-five mortality and its multi-dimensional determinants across 45 African nations (2000–2021)**

| Variables | (1) | (2) | (3) | (4) | (5) | (6) | (7) | (8) | (9) | (10) | (11) | (12) | (13) | (14) |
| --- | --- | --- | --- | --- | --- | --- | --- | --- | --- | --- | --- | --- | --- | --- |
| (1) mortality_u5 | 1.000 |  |  |  |  |  |  |  |  |  |  |  |  |  |
| (2) gdp_per_capita | -0.300 | 1.000 |  |  |  |  |  |  |  |  |  |  |  |  |
| (3) corruption_rank | -0.428 | 0.186 | 1.000 |  |  |  |  |  |  |  |  |  |  |  |
| (4) imm_dtp | -0.635 | 0.018 | 0.547 | 1.000 |  |  |  |  |  |  |  |  |  |  |
| (5) imm_measles | -0.576 | -0.002 | 0.538 | 0.937 | 1.000 |  |  |  |  |  |  |  |  |  |
| (6) malaria_inc | 0.672 | -0.304 | -0.397 | -0.403 | -0.388 | 1.000 |  |  |  |  |  |  |  |  |
| (7) health_expenditure | -0.062 | -0.104 | 0.277 | 0.324 | 0.330 | -0.133 | 1.000 |  |  |  |  |  |  |  |
| (8) adolescent_fert | 0.660 | -0.172 | -0.466 | -0.604 | -0.560 | 0.635 | -0.139 | 1.000 |  |  |  |  |  |  |
| (9) fert_rate | 0.744 | -0.466 | -0.562 | -0.535 | -0.507 | 0.681 | -0.179 | 0.763 | 1.000 |  |  |  |  |  |
| (10) air_pollution | 0.272 | -0.247 | -0.137 | -0.121 | -0.122 | 0.255 | -0.200 | 0.141 | 0.341 | 1.000 |  |  |  |  |
| (11) adolescent_fert squared | 0.670 | -0.121 | -0.456 | -0.629 | -0.569 | 0.606 | -0.156 | 0.970 | 0.741 | 0.151 | 1.000 |  |  |  |
| (12) ln_basic_sanitationn | -0.545 | 0.517 | 0.307 | 0.425 | 0.424 | -0.506 | 0.061 | -0.587 | -0.624 | -0.141 | -0.526 | 1.000 |  |  |
| (13) D2_clean_fuel | 0.058 | -0.079 | -0.028 | -0.052 | -0.055 | 0.080 | -0.035 | 0.043 | 0.077 | 0.033 | 0.031 | -0.096 | 1.000 |  |
| (14) gdp_squared | -0.105 | 0.893 | 0.024 | -0.091 | -0.109 | -0.093 | -0.136 | 0.030 | -0.207 | -0.172 | 0.076 | 0.314 | -0.059 | 1.000 |
| Source: Authors’ compilation | | | | | | | | | | | | | | |

**Note:** Under-five mortality rate (per 1,000 live births); gdp_per_capita: Gross Domestic Product per capita (current US$); corruption_rank: Control of Corruption percentile rank (0–100); imm_dtp: Diphtheria-Tetanus-Pertussis immunisation coverage (% of children ages 12–23 months); imm_measles: Measles immunisation coverage (%); malaria_inc: Incidence of malaria (new cases per 1,000 population at risk); health_expenditure: Current health expenditure (% of GDP); adolescent_fert: Adolescent fertility rate (births per 1,000 women ages 15–19); fert_rate: Total fertility rate (births per woman); air_pollution: PM2.5 mean annual exposure (micrograms per cubic meter); adolescent_fert_squared: Square of the adolescent fertility rate (used to capture non-linear risks); ln_basic_sanitation: Natural log of the percentage of the population using at least basic sanitation services; D2_clean_fuel: Second difference of access to clean fuels and technologies for cooking (% of population); gdp_squared: Square of the Gross Domestic Product per capita (used to model diminishing marginal returns).

**Table 7.B: Results of the Variance Inflation Factor analysis for independent variables used in the regional panel regression of the study (2000–2021)**

|  | VIF | 1/VIF |
| --- | --- | --- |
| adolescent fert | 21.526 | .046 |
| adolescent fert squared | 20.234 | .049 |
| gdp per capita | 10.622 | .094 |
| imm dtp | 9.614 | .104 |
| imm measles | 8.787 | .114 |
| gdp squared | 7.387 | .135 |
| fert rate | 4.959 | .202 |
| ln basic sanitation | 2.504 | .399 |
| malaria inc | 2.056 | .486 |
| corruption rank | 1.804 | .554 |
| air pollution | 1.251 | .799 |
| health expenditure | 1.23 | .813 |
| D2 clean fuel | 1.016 | .984 |
| Mean VIF | 7.153 | . |

Source: Authors’ compilation

**Note:** Under-five mortality rate (per 1,000 live births); gdp_per_capita: Gross Domestic Product per capita (current US$); corruption_rank: Control of Corruption percentile rank (0–100); imm_dtp: Diphtheria-Tetanus-Pertussis immunisation coverage (% of children ages 12–23 months); imm_measles: Measles immunisation coverage (%); malaria_inc: Incidence of malaria (new cases per 1,000 population at risk); health_expenditure: Current health expenditure (% of GDP); adolescent_fert: Adolescent fertility rate (births per 1,000 women ages 15–19); fert_rate: Total fertility rate (births per woman); air_pollution: PM2.5 mean annual exposure (micrograms per cubic meter); adolescent_fert_squared: Square of the adolescent fertility rate (used to capture non-linear risks); ln_basic_sanitation: Natural log of the percentage of the population using at least basic sanitation services; D2_clean_fuel: Second difference of access to clean fuels and technologies for cooking (% of population); gdp_squared: Square of the Gross Domestic Product per capita (used to model diminishing marginal returns). VIF: Variance Inflation Factor; 1/VIF: Tolerance level. VIF values below 10 are generally considered acceptable for social science research, though values above 5 (as seen for fertility and GDP terms) are expected in models utilizing quadratic transformations due to inherent structural correlation.
