# Supplementary material for: Determinants of under-five mortality in Africa: evidence from a two-decade panel analysis for public health policy
Source: Arch Public Health. 2026 Jun 17;84:193. doi: 10.1186/s13690-026-01994-0 (PMC13520361; doi:10.1186/s13690-026-01994-0)
Supplement: Supplementary file 7 [file 13690_2026_1994_MOESM8_ESM.docx]

**Appendix 8: Regional panel regression results for the full candidate-set of potential determinants of under-five mortality across 45 African nations: Stepwise backward elimination process and model specification diagnostics (2000–2021)**

**Table 8.A: regional panel regression estimates for the full candidate-set of determinants identifying associations with under-five mortality in African region using stepwise backward elimination (2000–2021)**

**Regression results**

| mortality_u5 | Coef. | | St.Err. | t-value | | p-value | [95% Conf | | Interval] | | Sig |
| --- | --- | --- | --- | --- | --- | --- | --- | --- | --- | --- | --- |
| gdp_per_capita | .004 | | .003 | 1.51 | | .131 | -.001 | | .009 | |  |
| corruption_rank | -.073 | | .195 | -0.37 | | .708 | -.454 | | .308 | |  |
| imm_dtp | -.529 | | .114 | -4.63 | | 0 | -.753 | | -.305 | | *** |
| imm_measles | .031 | | .11 | 0.28 | | .782 | -.185 | | .246 | |  |
| malaria_inc | .045 | | .01 | 4.68 | | 0 | .026 | | .064 | | *** |
| health_expenditure | -.179 | | .713 | -0.25 | | .801 | -1.577 | | 1.218 | |  |
| adolescent_fert | -.589 | | .148 | -3.99 | | 0 | -.879 | | -.3 | | *** |
| fert_rate | 25.026 | | 2.18 | 11.48 | | 0 | 20.753 | | 29.3 | | *** |
| air_pollution | .119 | | .357 | 0.33 | | .739 | -.58 | | .818 | |  |
| D2_clean_fuel | -1.296 | | 2.483 | -0.52 | | .602 | -6.163 | | 3.57 | |  |
| ln_basic_sanitation | -14.605 | | 2.994 | -4.88 | | 0 | -20.474 | | -8.736 | | *** |
| gdp_squared | 0 | | 0 | -0.89 | | .372 | 0 | | 0 | |  |
| air_pollution_squa~d | -.002 | | .003 | -0.66 | | .51 | -.009 | | .004 | |  |
| adolescent_fert_sq~d | .002 | | .001 | 3.41 | | .001 | .001 | | .003 | | *** |
| corruption_rank_sq~d | .003 | | .003 | 1.27 | | .205 | -.002 | | .008 | |  |
| lag_gdp_per_capita | -.008 | | .002 | -3.22 | | .001 | -.013 | | -.003 | | *** |
| lag_gdp_squared | 0 | | 0 | 2.60 | | .009 | 0 | | 0 | | *** |
| lag_health_expendi~e | -.262 | | .714 | -0.37 | | .714 | -1.663 | | 1.138 | |  |
| Constant | 81.966 | | 19.921 | 4.11 | | 0 | 42.922 | | 121.01 | | *** |
|  | | | | | | | | | | | |
| Mean dependent var | | 84.318 | | | SD dependent var | | | 41.837 | |  |  |
| Overall r-squared | | 0.640 | | | Number of obs | | | 900 | |  |  |
| Chi-square | | 1390.573 | | | Prob > chi2 | | | 0.000 | |  |  |
| R-squared within | | 0.622 | | | R-squared between | | | 0.679 | |  |  |
| **** p<.01, ** p<.05, * p<.1* | | | | | | | | | | | |
|  | | | | | | | | | | | |

**Regression results**

| mortality_u5 | Coef. | | St.Err. | t-value | | p-value | [95% Conf | | Interval] | | Sig |
| --- | --- | --- | --- | --- | --- | --- | --- | --- | --- | --- | --- |
| gdp_per_capita | .004 | | .003 | 1.51 | | .131 | -.001 | | .009 | |  |
| corruption_rank | -.074 | | .194 | -0.38 | | .704 | -.455 | | .307 | |  |
| imm_dtp | -.504 | | .075 | -6.73 | | 0 | -.651 | | -.357 | | *** |
| malaria_inc | .045 | | .01 | 4.69 | | 0 | .026 | | .064 | | *** |
| health_expenditure | -.184 | | .712 | -0.26 | | .796 | -1.58 | | 1.212 | |  |
| adolescent_fert | -.591 | | .148 | -4.01 | | 0 | -.881 | | -.302 | | *** |
| fert_rate | 25.058 | | 2.182 | 11.48 | | 0 | 20.78 | | 29.335 | | *** |
| air_pollution | .117 | | .357 | 0.33 | | .744 | -.583 | | .817 | |  |
| D2_clean_fuel | -1.291 | | 2.48 | -0.52 | | .603 | -6.152 | | 3.57 | |  |
| ln_basic_sanitation | -14.612 | | 2.995 | -4.88 | | 0 | -20.482 | | -8.741 | | *** |
| gdp_squared | 0 | | 0 | -0.90 | | .368 | 0 | | 0 | |  |
| air_pollution_squa~d | -.002 | | .003 | -0.65 | | .515 | -.009 | | .004 | |  |
| adolescent_fert_sq~d | .002 | | .001 | 3.44 | | .001 | .001 | | .003 | | *** |
| corruption_rank_sq~d | .003 | | .003 | 1.27 | | .203 | -.002 | | .008 | |  |
| lag_gdp_per_capita | -.008 | | .002 | -3.22 | | .001 | -.013 | | -.003 | | *** |
| lag_gdp_squared | 0 | | 0 | 2.60 | | .009 | 0 | | 0 | | *** |
| lag_health_expendi~e | -.266 | | .714 | -0.37 | | .71 | -1.664 | | 1.133 | |  |
| Constant | 82.41 | | 19.898 | 4.14 | | 0 | 43.411 | | 121.409 | | *** |
|  | | | | | | | | | | | |
| Mean dependent var | | 84.318 | | | SD dependent var | | | 41.837 | |  |  |
| Overall r-squared | | 0.640 | | | Number of obs | | | 900 | |  |  |
| Chi-square | | 1392.330 | | | Prob > chi2 | | | 0.000 | |  |  |
| R-squared within | | 0.622 | | | R-squared between | | | 0.679 | |  |  |
| **** p<.01, ** p<.05, * p<.1* | | | | | | | | | | | |
|  | | | | | | | | | | | |

**Regression results**

| mortality_u5 | Coef. | | St.Err. | t-value | | p-value | [95% Conf | | Interval] | | Sig |
| --- | --- | --- | --- | --- | --- | --- | --- | --- | --- | --- | --- |
| gdp_per_capita | .004 | | .003 | 1.56 | | .119 | -.001 | | .009 | |  |
| corruption_rank | -.072 | | .194 | -0.37 | | .712 | -.452 | | .309 | |  |
| imm_dtp | -.505 | | .075 | -6.75 | | 0 | -.652 | | -.359 | | *** |
| malaria_inc | .045 | | .01 | 4.70 | | 0 | .026 | | .064 | | *** |
| adolescent_fert | -.591 | | .147 | -4.02 | | 0 | -.88 | | -.303 | | *** |
| fert_rate | 25.031 | | 2.177 | 11.50 | | 0 | 20.765 | | 29.297 | | *** |
| air_pollution | .124 | | .356 | 0.35 | | .727 | -.574 | | .822 | |  |
| D2_clean_fuel | -1.301 | | 2.481 | -0.52 | | .6 | -6.163 | | 3.562 | |  |
| ln_basic_sanitation | -14.58 | | 2.988 | -4.88 | | 0 | -20.437 | | -8.724 | | *** |
| gdp_squared | 0 | | 0 | -0.93 | | .353 | 0 | | 0 | |  |
| air_pollution_squa~d | -.002 | | .003 | -0.67 | | .502 | -.009 | | .004 | |  |
| adolescent_fert_sq~d | .002 | | .001 | 3.45 | | .001 | .001 | | .003 | | *** |
| corruption_rank_sq~d | .003 | | .003 | 1.27 | | .203 | -.002 | | .008 | |  |
| lag_gdp_per_capita | -.008 | | .002 | -3.26 | | .001 | -.013 | | -.003 | | *** |
| lag_gdp_squared | 0 | | 0 | 2.62 | | .009 | 0 | | 0 | | *** |
| lag_health_expendi~e | -.404 | | .45 | -0.90 | | .369 | -1.286 | | .478 | |  |
| Constant | 81.943 | | 19.813 | 4.14 | | 0 | 43.11 | | 120.776 | | *** |
|  | | | | | | | | | | | |
| Mean dependent var | | 84.318 | | | SD dependent var | | | 41.837 | |  |  |
| Overall r-squared | | 0.641 | | | Number of obs | | | 900 | |  |  |
| Chi-square | | 1393.252 | | | Prob > chi2 | | | 0.000 | |  |  |
| R-squared within | | 0.622 | | | R-squared between | | | 0.680 | |  |  |
| **** p<.01, ** p<.05, * p<.1* | | | | | | | | | | | |
|  | | | | | | | | | | | |

**Regression results**

| mortality_u5 | Coef. | | St.Err. | t-value | | p-value | [95% Conf | | Interval] | | Sig |
| --- | --- | --- | --- | --- | --- | --- | --- | --- | --- | --- | --- |
| gdp_per_capita | .004 | | .003 | 1.56 | | .12 | -.001 | | .009 | |  |
| corruption_rank | -.074 | | .194 | -0.38 | | .704 | -.454 | | .306 | |  |
| imm_dtp | -.508 | | .075 | -6.80 | | 0 | -.654 | | -.362 | | *** |
| malaria_inc | .045 | | .01 | 4.78 | | 0 | .027 | | .064 | | *** |
| adolescent_fert | -.591 | | .147 | -4.02 | | 0 | -.879 | | -.303 | | *** |
| fert_rate | 25.034 | | 2.17 | 11.54 | | 0 | 20.782 | | 29.287 | | *** |
| D2_clean_fuel | -1.345 | | 2.479 | -0.54 | | .587 | -6.204 | | 3.514 | |  |
| ln_basic_sanitation | -14.512 | | 2.981 | -4.87 | | 0 | -20.354 | | -8.67 | | *** |
| gdp_squared | 0 | | 0 | -0.93 | | .352 | 0 | | 0 | |  |
| air_pollution_squa~d | -.001 | | .001 | -1.22 | | .224 | -.003 | | .001 | |  |
| adolescent_fert_sq~d | .002 | | .001 | 3.44 | | .001 | .001 | | .003 | | *** |
| corruption_rank_sq~d | .003 | | .003 | 1.28 | | .2 | -.002 | | .008 | |  |
| lag_gdp_per_capita | -.008 | | .002 | -3.26 | | .001 | -.013 | | -.003 | | *** |
| lag_gdp_squared | 0 | | 0 | 2.62 | | .009 | 0 | | 0 | | *** |
| lag_health_expendi~e | -.399 | | .45 | -0.89 | | .375 | -1.28 | | .483 | |  |
| Constant | 84.681 | | 18.103 | 4.68 | | 0 | 49.199 | | 120.163 | | *** |
|  | | | | | | | | | | | |
| Mean dependent var | | 84.318 | | | SD dependent var | | | 41.837 | |  |  |
| Overall r-squared | | 0.639 | | | Number of obs | | | 900 | |  |  |
| Chi-square | | 1394.071 | | | Prob > chi2 | | | 0.000 | |  |  |
| R-squared within | | 0.622 | | | R-squared between | | | 0.678 | |  |  |
| **** p<.01, ** p<.05, * p<.1* | | | | | | | | | | | |
|  | | | | | | | | | | | |

**Regression results**

| mortality_u5 | Coef. | | St.Err. | t-value | | p-value | [95% Conf | | Interval] | | Sig |
| --- | --- | --- | --- | --- | --- | --- | --- | --- | --- | --- | --- |
| gdp_per_capita | .004 | | .003 | 1.57 | | .117 | -.001 | | .009 | |  |
| imm_dtp | -.511 | | .074 | -6.91 | | 0 | -.656 | | -.366 | | *** |
| malaria_inc | .046 | | .009 | 4.83 | | 0 | .027 | | .064 | | *** |
| adolescent_fert | -.591 | | .147 | -4.02 | | 0 | -.879 | | -.303 | | *** |
| fert_rate | 24.974 | | 2.158 | 11.57 | | 0 | 20.744 | | 29.203 | | *** |
| D2_clean_fuel | -1.318 | | 2.476 | -0.53 | | .595 | -6.17 | | 3.534 | |  |
| ln_basic_sanitation | -14.528 | | 2.984 | -4.87 | | 0 | -20.377 | | -8.68 | | *** |
| gdp_squared | 0 | | 0 | -0.95 | | .343 | 0 | | 0 | |  |
| air_pollution_squa~d | -.001 | | .001 | -1.22 | | .221 | -.003 | | .001 | |  |
| adolescent_fert_sq~d | .002 | | .001 | 3.45 | | .001 | .001 | | .003 | | *** |
| corruption_rank_sq~d | .002 | | .001 | 2.59 | | .01 | .001 | | .004 | | *** |
| lag_gdp_per_capita | -.008 | | .002 | -3.26 | | .001 | -.013 | | -.003 | | *** |
| lag_gdp_squared | 0 | | 0 | 2.62 | | .009 | 0 | | 0 | | *** |
| lag_health_expendi~e | -.397 | | .449 | -0.88 | | .377 | -1.277 | | .484 | |  |
| Constant | 84.12 | | 18.049 | 4.66 | | 0 | 48.744 | | 119.496 | | *** |
|  | | | | | | | | | | | |
| Mean dependent var | | 84.318 | | | SD dependent var | | | 41.837 | |  |  |
| Overall r-squared | | 0.638 | | | Number of obs | | | 900 | |  |  |
| Chi-square | | 1395.783 | | | Prob > chi2 | | | 0.000 | |  |  |
| R-squared within | | 0.622 | | | R-squared between | | | 0.676 | |  |  |
| **** p<.01, ** p<.05, * p<.1* | | | | | | | | | | | |
|  | | | | | | | | | | | |

**Regression results**

| mortality_u5 | Coef. | | St.Err. | t-value | | p-value | [95% Conf | | Interval] | | Sig |
| --- | --- | --- | --- | --- | --- | --- | --- | --- | --- | --- | --- |
| gdp_per_capita | .003 | | .003 | 1.01 | | .313 | -.002 | | .007 | |  |
| imm_dtp | -.554 | | .068 | -8.12 | | 0 | -.688 | | -.42 | | *** |
| malaria_inc | .051 | | .009 | 5.49 | | 0 | .033 | | .069 | | *** |
| adolescent_fert | -.671 | | .144 | -4.68 | | 0 | -.952 | | -.39 | | *** |
| fert_rate | 25.502 | | 2.095 | 12.17 | | 0 | 21.396 | | 29.608 | | *** |
| ln_basic_sanitation | -14.825 | | 2.802 | -5.29 | | 0 | -20.317 | | -9.332 | | *** |
| gdp_squared | 0 | | 0 | -0.44 | | .661 | 0 | | 0 | |  |
| air_pollution_squa~d | -.001 | | .001 | -1.28 | | .201 | -.003 | | .001 | |  |
| adolescent_fert_sq~d | .002 | | .001 | 3.94 | | 0 | .001 | | .004 | | *** |
| corruption_rank_sq~d | .002 | | .001 | 2.43 | | .015 | 0 | | .004 | | ** |
| lag_gdp_per_capita | -.007 | | .002 | -2.90 | | .004 | -.012 | | -.002 | | *** |
| lag_gdp_squared | 0 | | 0 | 2.34 | | .019 | 0 | | 0 | | ** |
| lag_health_expendi~e | -.636 | | .434 | -1.47 | | .143 | -1.487 | | .215 | |  |
| Constant | 93.536 | | 17.198 | 5.44 | | 0 | 59.829 | | 127.243 | | *** |
|  | | | | | | | | | | | |
| Mean dependent var | | 86.305 | | | SD dependent var | | | 42.984 | |  |  |
| Overall r-squared | | 0.647 | | | Number of obs | | | 945 | |  |  |
| Chi-square | | 1695.598 | | | Prob > chi2 | | | 0.000 | |  |  |
| R-squared within | | 0.660 | | | R-squared between | | | 0.679 | |  |  |
| **** p<.01, ** p<.05, * p<.1* | | | | | | | | | | | |
|  | | | | | | | | | | | |

**Regression results**

| mortality_u5 | Coef. | | St.Err. | t-value | | p-value | [95% Conf | | Interval] | | Sig |
| --- | --- | --- | --- | --- | --- | --- | --- | --- | --- | --- | --- |
| gdp_per_capita | .002 | | .001 | 1.40 | | .161 | -.001 | | .004 | |  |
| imm_dtp | -.557 | | .068 | -8.16 | | 0 | -.691 | | -.423 | | *** |
| malaria_inc | .051 | | .009 | 5.49 | | 0 | .032 | | .069 | | *** |
| adolescent_fert | -.673 | | .143 | -4.72 | | 0 | -.953 | | -.393 | | *** |
| fert_rate | 25.374 | | 2.083 | 12.18 | | 0 | 21.291 | | 29.458 | | *** |
| ln_basic_sanitation | -14.653 | | 2.787 | -5.26 | | 0 | -20.115 | | -9.191 | | *** |
| air_pollution_squa~d | -.001 | | .001 | -1.34 | | .179 | -.003 | | .001 | |  |
| adolescent_fert_sq~d | .002 | | .001 | 4.00 | | 0 | .001 | | .004 | | *** |
| corruption_rank_sq~d | .002 | | .001 | 2.50 | | .013 | 0 | | .004 | | ** |
| lag_gdp_per_capita | -.006 | | .002 | -3.84 | | 0 | -.01 | | -.003 | | *** |
| lag_gdp_squared | 0 | | 0 | 3.20 | | .001 | 0 | | 0 | | *** |
| lag_health_expendi~e | -.633 | | .434 | -1.46 | | .145 | -1.483 | | .217 | |  |
| Constant | 93.941 | | 17.105 | 5.49 | | 0 | 60.416 | | 127.465 | | *** |
|  | | | | | | | | | | | |
| Mean dependent var | | 86.305 | | | SD dependent var | | | 42.984 | |  |  |
| Overall r-squared | | 0.647 | | | Number of obs | | | 945 | |  |  |
| Chi-square | | 1694.593 | | | Prob > chi2 | | | 0.000 | |  |  |
| R-squared within | | 0.659 | | | R-squared between | | | 0.680 | |  |  |
| **** p<.01, ** p<.05, * p<.1* | | | | | | | | | | | |
|  | | | | | | | | | | | |

**Regression results**

| mortality_u5 | Coef. | | St.Err. | t-value | | p-value | [95% Conf | | Interval] | | Sig |
| --- | --- | --- | --- | --- | --- | --- | --- | --- | --- | --- | --- |
| imm_dtp | -.558 | | .068 | -8.17 | | 0 | -.691 | | -.424 | | *** |
| malaria_inc | .05 | | .009 | 5.48 | | 0 | .032 | | .068 | | *** |
| adolescent_fert | -.688 | | .142 | -4.86 | | 0 | -.966 | | -.41 | | *** |
| fert_rate | 25.351 | | 2.077 | 12.21 | | 0 | 21.28 | | 29.421 | | *** |
| ln_basic_sanitation | -14.394 | | 2.777 | -5.18 | | 0 | -19.836 | | -8.952 | | *** |
| air_pollution_squa~d | -.001 | | .001 | -1.49 | | .136 | -.003 | | 0 | |  |
| adolescent_fert_sq~d | .002 | | .001 | 4.20 | | 0 | .001 | | .004 | | *** |
| corruption_rank_sq~d | .002 | | .001 | 2.60 | | .009 | .001 | | .004 | | *** |
| lag_gdp_per_capita | -.005 | | .001 | -3.97 | | 0 | -.007 | | -.002 | | *** |
| lag_gdp_squared | 0 | | 0 | 2.95 | | .003 | 0 | | 0 | | *** |
| lag_health_expendi~e | -.64 | | .434 | -1.47 | | .14 | -1.491 | | .211 | |  |
| Constant | 93.848 | | 17.066 | 5.50 | | 0 | 60.401 | | 127.296 | | *** |
|  | | | | | | | | | | | |
| Mean dependent var | | 86.305 | | | SD dependent var | | | 42.984 | |  |  |
| Overall r-squared | | 0.647 | | | Number of obs | | | 945 | |  |  |
| Chi-square | | 1688.587 | | | Prob > chi2 | | | 0.000 | |  |  |
| R-squared within | | 0.659 | | | R-squared between | | | 0.679 | |  |  |
| **** p<.01, ** p<.05, * p<.1* | | | | | | | | | | | |
|  | | | | | | | | | | | |

**Regression results**

| mortality_u5 | Coef. | | St.Err. | t-value | | p-value | [95% Conf | | Interval] | | Sig |
| --- | --- | --- | --- | --- | --- | --- | --- | --- | --- | --- | --- |
| imm_dtp | -.551 | | .068 | -8.07 | | 0 | -.685 | | -.417 | | *** |
| malaria_inc | .05 | | .009 | 5.47 | | 0 | .032 | | .069 | | *** |
| adolescent_fert | -.679 | | .143 | -4.75 | | 0 | -.958 | | -.399 | | *** |
| fert_rate | 25.26 | | 2.086 | 12.11 | | 0 | 21.173 | | 29.348 | | *** |
| ln_basic_sanitation | -14.733 | | 2.795 | -5.27 | | 0 | -20.21 | | -9.255 | | *** |
| adolescent_fert_sq~d | .002 | | .001 | 4.10 | | 0 | .001 | | .004 | | *** |
| corruption_rank_sq~d | .002 | | .001 | 2.46 | | .014 | 0 | | .004 | | ** |
| lag_gdp_per_capita | -.005 | | .001 | -3.99 | | 0 | -.007 | | -.002 | | *** |
| lag_gdp_squared | 0 | | 0 | 2.98 | | .003 | 0 | | 0 | | *** |
| lag_health_expendi~e | -.67 | | .434 | -1.54 | | .122 | -1.521 | | .18 | |  |
| Constant | 92.396 | | 17.131 | 5.39 | | 0 | 58.82 | | 125.973 | | *** |
|  | | | | | | | | | | | |
| Mean dependent var | | 86.305 | | | SD dependent var | | | 42.984 | |  |  |
| Overall r-squared | | 0.645 | | | Number of obs | | | 945 | |  |  |
| Chi-square | | 1689.010 | | | Prob > chi2 | | | 0.000 | |  |  |
| R-squared within | | 0.660 | | | R-squared between | | | 0.679 | |  |  |
| **** p<.01, ** p<.05, * p<.1* | | | | | | | | | | | |
|  | | | | | | | | | | | |

**Regression results**

| mortality_u5 | Coef. | | St.Err. | t-value | | p-value | [95% Conf | | Interval] | | Sig |
| --- | --- | --- | --- | --- | --- | --- | --- | --- | --- | --- | --- |
| imm_dtp | -.544 | | .068 | -7.95 | | 0 | -.678 | | -.41 | | *** |
| malaria_inc | .051 | | .009 | 5.56 | | 0 | .033 | | .069 | | *** |
| adolescent_fert | -.711 | | .142 | -4.99 | | 0 | -.99 | | -.432 | | *** |
| fert_rate | 24.543 | | 2.07 | 11.86 | | 0 | 20.486 | | 28.6 | | *** |
| ln_basic_sanitation | -15.544 | | 2.779 | -5.59 | | 0 | -20.99 | | -10.098 | | *** |
| adolescent_fert_sq~d | .003 | | .001 | 4.28 | | 0 | .001 | | .004 | | *** |
| lag_gdp_per_capita | -.005 | | .001 | -3.97 | | 0 | -.007 | | -.002 | | *** |
| lag_gdp_squared | 0 | | 0 | 2.95 | | .003 | 0 | | 0 | | *** |
| lag_health_expendi~e | -.586 | | .434 | -1.35 | | .177 | -1.437 | | .264 | |  |
| Constant | 102.382 | | 16.679 | 6.14 | | 0 | 69.692 | | 135.072 | | *** |
|  | | | | | | | | | | | |
| Mean dependent var | | 86.305 | | | SD dependent var | | | 42.984 | |  |  |
| Overall r-squared | | 0.639 | | | Number of obs | | | 945 | |  |  |
| Chi-square | | 1672.925 | | | Prob > chi2 | | | 0.000 | |  |  |
| R-squared within | | 0.662 | | | R-squared between | | | 0.676 | |  |  |
| **** p<.01, ** p<.05, * p<.1* | | | | | | | | | | | |
|  | | | | | | | | | | | |

**Regression results**

| mortality_u5 | Coef. | | St.Err. | t-value | | p-value | [95% Conf | | Interval] | | Sig |
| --- | --- | --- | --- | --- | --- | --- | --- | --- | --- | --- | --- |
| imm_dtp | -.454 | | .069 | -6.56 | | 0 | -.59 | | -.318 | | *** |
| malaria_inc | .052 | | .01 | 5.41 | | 0 | .033 | | .071 | | *** |
| adolescent_fert | -.752 | | .182 | -4.12 | | 0 | -1.111 | | -.394 | | *** |
| fert_rate | 29.757 | | 2.59 | 11.49 | | 0 | 24.673 | | 34.841 | | *** |
| ln_basic_sanitation | -19.02 | | 3.276 | -5.81 | | 0 | -25.449 | | -12.59 | | *** |
| adolescent_fert_sq~d | .002 | | .001 | 3.16 | | .002 | .001 | | .004 | | *** |
| lag_gdp_per_capita | -.007 | | .001 | -5.89 | | 0 | -.009 | | -.005 | | *** |
| lag_gdp_squared | 0 | | 0 | 4.46 | | 0 | 0 | | 0 | | *** |
| lag_health_expendi~e | -.774 | | .435 | -1.78 | | .075 | -1.628 | | .079 | | * |
| Constant | 95.858 | | 18.542 | 5.17 | | 0 | 59.466 | | 132.249 | | *** |
|  | | | | | | | | | | | |
| Mean dependent var | | 86.305 | | | SD dependent var | | | 42.984 | |  |  |
| R-squared | | 0.667 | | | Number of obs | | | 945 | |  |  |
| F-test | | 198.315 | | | Prob > F | | | 0.000 | |  |  |
| Akaike crit. (AIC) | | 7747.074 | | | Bayesian crit. (BIC) | | | 7795.586 | |  |  |
| **** p<.01, ** p<.05, * p<.1* | | | | | | | | | | | |
|  | | | | | | | | | | | |

**Regression results**

| mortality_u5 | Coef. | | St.Err. | t-value | | p-value | [95% Conf | | Interval] | | Sig |
| --- | --- | --- | --- | --- | --- | --- | --- | --- | --- | --- | --- |
| imm_dtp | -.544 | | .068 | -7.95 | | 0 | -.678 | | -.41 | | *** |
| malaria_inc | .051 | | .009 | 5.56 | | 0 | .033 | | .069 | | *** |
| adolescent_fert | -.711 | | .142 | -4.99 | | 0 | -.99 | | -.432 | | *** |
| fert_rate | 24.543 | | 2.07 | 11.86 | | 0 | 20.486 | | 28.6 | | *** |
| ln_basic_sanitation | -15.544 | | 2.779 | -5.59 | | 0 | -20.99 | | -10.098 | | *** |
| adolescent_fert_sq~d | .003 | | .001 | 4.28 | | 0 | .001 | | .004 | | *** |
| lag_gdp_per_capita | -.005 | | .001 | -3.97 | | 0 | -.007 | | -.002 | | *** |
| lag_gdp_squared | 0 | | 0 | 2.95 | | .003 | 0 | | 0 | | *** |
| lag_health_expendi~e | -.586 | | .434 | -1.35 | | .177 | -1.437 | | .264 | |  |
| Constant | 102.382 | | 16.679 | 6.14 | | 0 | 69.692 | | 135.072 | | *** |
|  | | | | | | | | | | | |
| Mean dependent var | | 86.305 | | | SD dependent var | | | 42.984 | |  |  |
| Overall r-squared | | 0.639 | | | Number of obs | | | 945 | |  |  |
| Chi-square | | 1672.925 | | | Prob > chi2 | | | 0.000 | |  |  |
| R-squared within | | 0.662 | | | R-squared between | | | 0.676 | |  |  |
| **** p<.01, ** p<.05, * p<.1* | | | | | | | | | | | |
|  | | | | | | | | | | | |

**Hausman Test**

|  | **Coef.** |
| --- | --- |
| **Chi-square test value** | **82.284** |
| **P-value** | **0.000** |

**Regression results**

| mortality_u5 | Coef. | | St.Err. | t-value | | p-value | [95% Conf | | Interval] | | Sig |
| --- | --- | --- | --- | --- | --- | --- | --- | --- | --- | --- | --- |
| imm_dtp | -.454 | | .109 | -4.18 | | 0 | -.673 | | -.235 | | *** |
| malaria_inc | .052 | | .025 | 2.09 | | .043 | .002 | | .102 | | ** |
| adolescent_fert | -.752 | | .338 | -2.23 | | .031 | -1.433 | | -.072 | | ** |
| fert_rate | 29.757 | | 6.82 | 4.36 | | 0 | 16.012 | | 43.502 | | *** |
| ln_basic_sanitation | -19.02 | | 6.8 | -2.80 | | .008 | -32.724 | | -5.315 | | *** |
| adolescent_fert_sq~d | .002 | | .001 | 1.74 | | .089 | 0 | | .005 | | * |
| lag_gdp_per_capita | -.007 | | .002 | -3.93 | | 0 | -.011 | | -.003 | | *** |
| lag_gdp_squared | 0 | | 0 | 3.40 | | .001 | 0 | | 0 | | *** |
| lag_health_expendi~e | -.774 | | .599 | -1.29 | | .203 | -1.982 | | .434 | |  |
| Constant | 95.858 | | 39.722 | 2.41 | | .02 | 15.803 | | 175.912 | | ** |
|  | | | | | | | | | | | |
| Mean dependent var | | 86.305 | | | SD dependent var | | | 42.984 | |  |  |
| R-squared | | 0.667 | | | Number of obs | | | 945 | |  |  |
| F-test | | 97.312 | | | Prob > F | | | 0.000 | |  |  |
| Akaike crit. (AIC) | | 7745.074 | | | Bayesian crit. (BIC) | | | 7788.735 | |  |  |
| Source: Authors’ compilation | | | | | | | | | | | |
|  | | | | | | | | | | | |

**_Note:_** **** p<.01, ** p<.05, * p<.1*_. mortality_u5: Under-five mortality rate (per 1,000 live births); gdp_per_capita: Gross Domestic Product per capita (current US$); corruption_rank: Control of Corruption percentile rank (0–100); imm_dtp: Diphtheria-Tetanus-Pertussis immunization coverage (% of children ages 12–23 months); imm_measles: Measles immunization coverage (%); malaria_inc: Incidence of malaria (new cases per 1,000 population at risk); health_expenditure: Current health expenditure (% of GDP); adolescent_fert: Adolescent fertility rate (births per 1,000 women ages 15–19); fert_rate: Total fertility rate (births per woman); air_pollution: PM2.5 mean annual exposure (micrograms per cubic meter); D2_clean_fuel: Second difference of access to clean fuels and technologies for cooking; ln_basic_sanitation: Natural log of the percentage of the population using at least basic sanitation services; gdp_squared: Square of the Gross Domestic Product per capita; air_pollution_squared: Square of the mean annual air pollution exposure; adolescent_fert_squared: Square of the adolescent fertility rate; corruption_rank_squared: Square of the corruption percentile rank; lag_gdp_per_capita: One-year lagged Gross Domestic Product per capita; lag_gdp_squared: One-year lagged square of Gross Domestic Product per capita; lag_health_expenditure: One-year lagged current health expenditure. Coef.: Regression coefficient; St.Err.: Heteroscedasticity-robust standard error clustered at the country level; t-value: Test statistic for the null hypothesis that the coefficient is zero; p-value: Probability value for statistical significance; [95% Conf Interval]: 95% confidence interval for the coefficient;_ **_Sig_**_: Significance level marker._
