# Supplementary material for: Determinants of under-five mortality in Africa: evidence from a two-decade panel analysis for public health policy
Source: Arch Public Health. 2026 Jun 17;84:193. doi: 10.1186/s13690-026-01994-0 (PMC13520361; doi:10.1186/s13690-026-01994-0)
Supplement: Supplementary file 8 [file 13690_2026_1994_MOESM9_ESM.docx]

**Appendix 9: Robustness and sensitivity analysis for regional determinants of under-five mortality across 45 African nations: Fixed-effects models with integrated year effects and Driscoll-Kraay robust standard errors (2000–2021)**

**Table 9.A: Regional panel regression estimates for under-five mortality across 45 African nations using a fixed-effects model with year fixed effects to control for common regional time-shocks (2000–2021)**

| mortality_u5 | Coef. | | St.Err. | t-value | | p-value | [95% Conf | | Interval] | | Sig |
| --- | --- | --- | --- | --- | --- | --- | --- | --- | --- | --- | --- |
| imm_dtpdtp | -.265 | | .099 | -2.67 | | .011 | -.465 | | -.065 | | ** |
| malaria_inc | .027 | | .018 | 1.45 | | .155 | -.01 | | .064 | |  |
| adolescent_fert | -.976 | | .294 | -3.32 | | .002 | -1.567 | | -.384 | | *** |
| fert_rate | 21.051 | | 5.869 | 3.59 | | .001 | 9.222 | | 32.88 | | *** |
| ln_basic_sanitation | -4.918 | | 5.785 | -0.85 | | .4 | -16.577 | | 6.741 | |  |
| adolescent_fert_sq~d | .003 | | .001 | 3.00 | | .004 | .001 | | .005 | | *** |
| lag_gdp_per_capita | .002 | | .002 | 0.98 | | .333 | -.002 | | .007 | |  |
| lag_gdp_squared | 0 | | 0 | -0.96 | | .341 | 0 | | 0 | |  |
| lag_health_expendi~e | -.32 | | .609 | -0.53 | | .602 | -1.548 | | .907 | |  |
| 2001b | 0 | | . | . | | . | . | | . | |  |
| 2002 | -3.187 | | .781 | -4.08 | | 0 | -4.761 | | -1.613 | | *** |
| 2003 | -6.43 | | 1.375 | -4.68 | | 0 | -9.201 | | -3.658 | | *** |
| 2004 | -10.052 | | 2.083 | -4.83 | | 0 | -14.25 | | -5.853 | | *** |
| 2005 | -13.832 | | 2.75 | -5.03 | | 0 | -19.374 | | -8.289 | | *** |
| 2006 | -17.885 | | 3.248 | -5.51 | | 0 | -24.432 | | -11.338 | | *** |
| 2007 | -20.945 | | 3.711 | -5.64 | | 0 | -28.424 | | -13.466 | | *** |
| 2008 | -24.182 | | 4.054 | -5.97 | | 0 | -32.351 | | -16.012 | | *** |
| 2009 | -19.572 | | 10.519 | -1.86 | | .069 | -40.772 | | 1.628 | | * |
| 2010 | -29.395 | | 4.391 | -6.69 | | 0 | -38.244 | | -20.546 | | *** |
| 2011 | -32.055 | | 4.482 | -7.15 | | 0 | -41.089 | | -23.021 | | *** |
| 2012 | -33.9 | | 4.747 | -7.14 | | 0 | -43.467 | | -24.333 | | *** |
| 2013 | -35.243 | | 4.919 | -7.16 | | 0 | -45.157 | | -25.329 | | *** |
| 2014 | -34.937 | | 5.837 | -5.98 | | 0 | -46.701 | | -23.172 | | *** |
| 2015 | -37.651 | | 5.097 | -7.39 | | 0 | -47.923 | | -27.38 | | *** |
| 2016 | -38.39 | | 5.1 | -7.53 | | 0 | -48.669 | | -28.111 | | *** |
| 2017 | -38.887 | | 5.515 | -7.05 | | 0 | -50.002 | | -27.773 | | *** |
| 2018 | -40.097 | | 5.403 | -7.42 | | 0 | -50.986 | | -29.208 | | *** |
| 2019 | -37.733 | | 7.555 | -4.99 | | 0 | -52.959 | | -22.507 | | *** |
| 2020 | -42.026 | | 5.595 | -7.51 | | 0 | -53.302 | | -30.75 | | *** |
| 2021 | -41.374 | | 6.008 | -6.89 | | 0 | -53.482 | | -29.265 | | *** |
| Constant | 104.892 | | 32.077 | 3.27 | | .002 | 40.244 | | 169.539 | | *** |
|  | | | | | | | | | | | |
| Mean dependent var | | 86.305 | | | SD dependent var | | | 42.984 | |  |  |
| R-squared | | 0.721 | | | Number of obs | | | 945 | |  |  |
| F-test | | 142.436 | | | Prob > F | | | 0.000 | |  |  |
| Akaike crit. (AIC) | | 7617.067 | | | Bayesian crit. (BIC) | | | 7757.751 | |  |  |
| Source: Authors’ compilation | | | | | | | | | | | |
|  | | | | | | | | | | | |

**Note:** **** p<.01, ** p<.05, * p<.1*. mortality_u5: Under-five mortality rate (per 1,000 live births); imm_dtpdtp: Diphtheria-Tetanus-Pertussis immunization coverage (% of children ages 12–23 months); malaria_inc: Incidence of malaria (new cases per 1,000 population at risk); adolescent_fert: Adolescent fertility rate (births per 1,000 women ages 15–19); fert_rate: Total fertility rate (births per woman); ln_basic_sanitation: Natural log of the percentage of the population using at least basic sanitation services (log-transformation used to capture diminishing marginal effects); adolescent_fert_squared: Square of the adolescent fertility rate (used to capture non-linear demographic risks); lag_gdp_per_capita: One-year lagged Gross Domestic Product per capita (current US$); lag_gdp_squared: One-year lagged square of Gross Domestic Product per capita; lag_health_expenditure: One-year lagged current health expenditure (% of GDP); Coef.: Regression coefficient; St.Err.: Standard error (clustered at country level); t-value: Test statistic; p-value: Probability value for statistical significance; [95% Conf Interval]: 95% confidence interval for the coefficient; Sig: Significance level marker. GDP and CHE are lagged by one year (t-1) to capture delayed transmission mechanisms and mitigate simultaneity bias, Basic sanitation is transformed using natural logarithms (ln) to capture diminishing marginal effects, Quadratic terms (lag GDP squared and adolescent fertility squared) are included to capture non-linear relationships. This table presents the results of the Fixed Effects (FE) estimator integrated with year fixed-effects to control for unobserved time-invariant country heterogeneity and common regional time-shocks (N=945 observations).

**Table 9.B: Regional panel regression estimates for under-five mortality across 45 African nations utilising Driscoll-Kraay standard errors to account for cross-sectional dependency and serial correlation (2000–2021)**

Regression with Driscoll-Kraay standard errors	Number of obs	=	945
Method: Fixed-effects regression	Number of groups =	45
Group variable (i): country_id	F( 9, 20)	=	2983.29
maximum lag: 2	Prob > F	=	0.0000
 within R-squared =	0.6670

|  | | | Drisc/Kraay | | | |  |  |  |  |
| --- | --- | --- | --- | --- | --- | --- | --- | --- | --- | --- |
| mortality_u5 | Coefficient | std. | | err. | t | P>t | | [95% | conf. | interval] |
| imm_dtpdtp | -0.454 | 0.056 | | -8.080 | 0.000 | -0.571 | | -0.337*** |  |  |
| malaria_inc | 0.052 | 0.016 | | 3.190 | 0.005 | 0.018 | | 0.086*** |  |  |
| adolescent_fert | -0.752 | 0.219 | | -3.440 | 0.003 | -1.209 | | -0.296*** |  |  |
| fert_rate | 29.757 | 3.325 | | 8.950 | 0.000 | 22.822 | | 36.692*** |  |  |
| ln_basic_sanitation | -19.020 | 2.878 | | -6.610 | 0.000 | -25.023 | | -13.017*** |  |  |
| adolescent_fert_squared | 0.002 | 0.001 | | 3.380 | 0.003 | 0.001 | | 0.003*** |  |  |
| lag_gdp_per_capita | -0.007 | 0.001 | | -7.950 | 0.000 | -0.009 | | -0.005*** |  |  |
| lag_gdp_squared | 0.000 | 0.000 | | 5.590 | 0.000 | 0.000 | | 0.000*** |  |  |
| lag_health_expenditure | -0.774 | 0.537 | | -1.440 | 0.165 | -1.895 | | 0.346 |  |  |
| _cons | 95.858 | 17.136 | | 5.590 | 0.000 | 60.114 | | 131.602 |  |  |
|  | | | | | | | | |  |  |

Source: Authors’ compilation

**Note:** mortality_u5: Under-five mortality rate (per 1,000 live births); imm_dtpdtp: Diphtheria-Tetanus-Pertussis immunization coverage (% of children ages 12–23 months); malaria_inc: Incidence of malaria (new cases per 1,000 population at risk); adolescent_fert: Adolescent fertility rate (births per 1,000 women ages 15–19); fert_rate: Total fertility rate (births per woman); ln_basic_sanitation: Natural log of the percentage of the population using at least basic sanitation services (log-transformation used to capture diminishing marginal effects); adolescent_fert_squared: Square of the adolescent fertility rate (used to capture non-linear demographic risks); lag_gdp_per_capita: One-year lagged Gross Domestic Product per capita (current US$); lag_gdp_squared: One-year lagged square of Gross Domestic Product per capita; lag_health_expenditure: One-year lagged current health expenditure (% of GDP); Coef.: Regression coefficient; St.Err.: Driscoll-Kraay robust; t-value: Test statistic; p-value: Probability value for statistical significance; [95% Conf Interval]: 95% confidence interval for the coefficient; Sig: Significance level marker. Estimates utilise Driscoll-Kraay robust standard errors, which are flexible to heteroskedasticity, serial correlation, and cross-sectional dependency across the 45-country panel. GDP and CHE are lagged by one year (t-1) to capture delayed transmission mechanisms and mitigate simultaneity bias, Basic sanitation is transformed using natural logarithms (ln) to capture diminishing marginal effects, Quadratic terms (lag GDP squared and adolescent fertility squared) are included to capture non-linear relationships. This table presents regional panel regression estimates utilising Driscoll-Kraay standard errors (N=945 observations).
